# Circ_0000811 acts as a miR-15b sponge and inhibits Prkar2a-mediated JAK2/STAT1 pathway to attenuate cerebral ischemic vertigo

**DOI:** 10.1038/s41420-022-01016-2

**Published:** 2022-05-04

**Authors:** Rui Huang, Weishuai Li, Dong Han, Yan Gao, Dongming Zheng, Guorong Bi

**Affiliations:** Department of Neurology, Shengjing Hospital of China Medical University, 110004 Shenyang, P.R. China

**Keywords:** Molecular biology, Diseases

## Abstract

Circular RNAs (circRNAs) have been noted to express in the brain and thus participate in various diseases related to the central nervous system. However, the potential role of circRNAs in cerebral ischemia (CI)-induced vertigo remains unknown. We initially predicted through bioinformatics analysis the poor expression of circ_0000811 related to CI. A mouse model of CI-induced vertigo was then established, which was validated by measurement of escape latency and medial vestibular nucleus (MVN) blood flow, with NeuN/Annexin counterstaining utilized to detect cell apoptosis in the MVN. An oxygen glucose deprivation (OGD)-exposed neuron-like cell model was further established for in vitro gain- and loss- of function assays, with flow cytometry performed to detect cell apoptosis. The poorly expressed circ_0000811, up-regulated miR-15b expression, and down-regulated Prkar2a expression were observed in both mice with CI-induced vertigo and OGD-exposed cells. Our data then demonstrated that circ_0000811 restoration alleviated CI-induced vertigo in mouse models, and that circ_0000811 acted as a miR-15b sponge to inhibit miR-15b expression. Prkar2a was validated as the target gene of miR-15b. Prkar2a restoration was subsequently revealed to repress OGD-induced neuronal apoptosis through JAK2/STAT1 signaling pathway inactivation. Furthermore, inactivation of the JAK2/STAT1 signaling pathway exerted an anti-apoptotic effect in OGD-induced neurons and an alleviatory effect in mice with CI-induced vertigo with Prkar2a overexpression and circ_0000811 overexpression. Taken together, our work suggests that circ_0000811 is involved in neuronal apoptosis of CI-induced vertigo and may be used as a biomarker for ameliorating CI-induced vertigo.

## Introduction

Cerebral stroke represents one of the most prevalent causes of death and disability worldwide, and almost 85% of cases are characterized as ischemia [1]. There was a high frequency of infarcts in posterior circulation related to cerebral ischemia (CI), which accounts for frequently experienced symptoms, including vertigo, headache, and nausea [2]. Further, vertigo/dizziness has been recognized as one of the commonest symptoms in patients with cerebral strokes, especially cerebellar ischemic stroke, and focal cerebellar stroke may even present with isolated vertigo without other symptoms [3, 4]. It has been suggested that cerebellar ischemic stroke, among syndromes of cerebellar stroke, ranks first among causes of isolated vascular vertigo, and that cerebellar infarction may cause spontaneous prolonged vertigo through a condition mimicking viral or post inflammation of the vestibular nerve [3–5]. To date, improvements have been made on therapeutic regimens for ischemic stroke, whereas the treatment for CI-induced vertigo has received less attention [6]. Herein, we aimed to investigate novel molecular mechanisms underlying CI-induced vertigo that may contribute to the development of effective treatment protocols.

Circular RNAs (circRNAs) refer to a series of non-coding RNAs with closed-loop structures without 5′ to 3′ polarity and are more stable than linear RNAs since they are free of exonuclease-dependent degradation [7]. It has been well-established that circRNAs can regulate gene expression through various mechanisms, one of which is based on their role of “microRNAs (miRNAs) sponges,” namely, competitively binding to miRNAs to repress their activity [8]. In recent years, circRNAs have been highlighted as a novel class of biomarkers for a variety of diseases, and, notably, the aberrant expression of several circRNAs (circ-camk4, circ_008018, HECTD1) has been implicated in CI and in neuronal injury induced by CI [9–11]. Nonetheless, the potential role of circRNAs in CI-induced vertigo remains to be established. In this study, we identified under-expression of circ_0000811 in brain tissues of mice with CI-induced vertigo through preliminary RNA sequencing analysis and then identified its binding sites with miR-15b through Starbase data analysis.

Interestingly, miR-15b has been reported for its stimulative effects on various cancers, such as lung adenocarcinoma, breast cancer, and gastric cancer, mainly by augmenting cell proliferation and migration [12–14]. Furthermore, a previous study has suggested that miR-15b expression was markedly increased after middle cerebral artery occlusion, and that the inhibition of miR-15b can stimulate the neuroprotective effect of sevoflurane treatment [15]. Additionally, down-regulated miR-15b expression by mild therapeutic hypothermia was found to protect against CI/reperfusion injury in rats [16]. Therefore, miR-15b was chosen as another focus of the study. Furthermore, through bioinformatics analysis we identified Prkar2a to be the downstream gene of miR-15b. Notably, Prkar2a has been priorly indicated to reversely regulate the JAK2/STAT1 axis [17], and the activation of the JAK2/STAT1 axis has been reported for its participation in cell apoptosis [18, 19]. Inhibition of JAK2/STAT1 by GJ-4 could alleviate memory impairment in focal CI/reperfusion in a rat model [20]. Based on the aforementioned evidence, we hypothesized in this study that circ_0000811 may affect CI-induced vertigo by modulating the miR-15b/Prkar2a/JAK2/STAT1 cascade, which may be implicated in neuronal apoptosis.

## Results

### Circ_0000811 is poorly expressed in mice of CI-induced vertigo

**Fig. 1:**
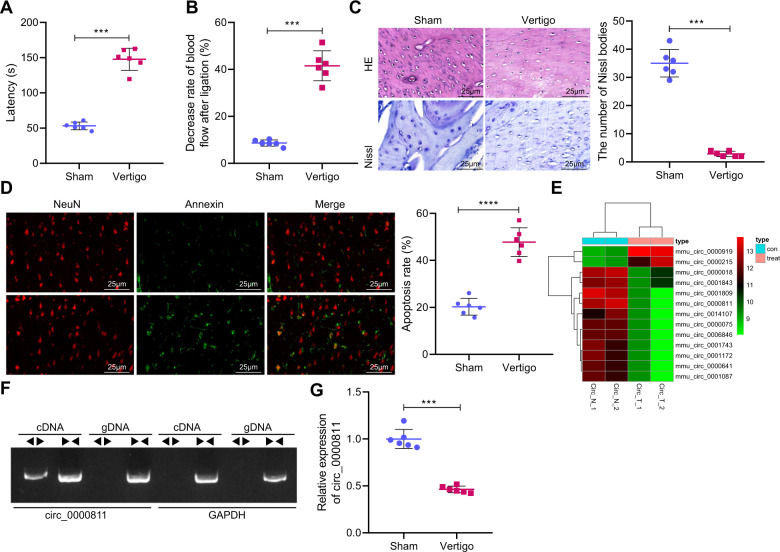
Circ_0000811 was down-regulated in brain tissues of mice with experimental CI-induced vertigo. **A** The escape latency of mice with CI-induced vertigo and sham-operated mice; **B** the decrease rate of blood flow in the MVN after ligation of CCA and SCA in mice with CI-induced vertigo and sham-operated mice; **C** H&E and Nissl staining to detect MVN histopathological changes in mice with CI-induced vertigo and sham-operated mice; **D** NeuN/Annexin counterstaining to detect the apoptotic rate of neurons in the MVN of mice with CI-induced vertigo and sham-operated mice; **E** the heat map of the expression of significantly differential circRNAs screened by circRNA sequencing for cerebral ischemic vertigo; **F** agarose gel electrophoresis to detect the amplification of circ_0000811 in cDNA and gDNA; **G** qRT-PCR to determine the level of circ_0000811 in the MVN of mice with CI-induced vertigo and sham-operated mice. ***p < 0.001 compared with the Sham group. The independent sample *t* test was applied for the comparison between two groups. CI cerebral ischemia, circRNA circular RNA, cDNA complementary DNA, gDNA genomic DNA, MVN medial vestibular nucleus, CCA common carotid artery, SCA superior cerebellar artery, H&E hematoxylin and eosin, qRT-PCR quantitative reverse-transcription polymerase chain reaction, NeuN neuronal nuclei.

For evaluating the establishment of CI-induced vertigo in mice, the escape latency and blood flow of mice were measured, and these mice exhibited prolonged escape latency and decreased blood flow rate of the MVN relative to sham-operated mice (Fig. 1A, B). Moreover, the MVN of sham-operated mice presented with clear outline, nucleus in the middle with obvious nucleolus, and evenly distributed Nissl bodies, whereas atrophic nucleus, nuclear fragmentation, and cytolysis were observed in the tissue of mice with CI-induced vertigo (Fig. 1C). Besides, the results of NeuN (a neuron marker)/Annexin (an apoptosis marker) counterstaining showed that compared with sham-operated mice, mice with CI-induced vertigo had significantly reduced neuronal apoptosis (Fig. 1D). These findings indicated that the mouse model of CI-induced vertigo was successfully established.

In order to further explore the role of circRNA in cerebral ischemic vertigo, we sequenced 2 pairs of brain tissues from normal mice and mice with CI-induced vertigo for circRNA sequencing and identification. Accordingly, two cerebral ischemic vertigo-related circRNAs with obviously up-regulated expression and 11 circRNAs with down-regulated expression were identified in brain tissues of mice with CI-induced vertigo, among which circ_0000811 was one of those exhibiting most obvious down-regulation (Fig. 1E). Further, analysis of the circular structure of circ_0000811 revealed that it can be amplified from complementary DNA (cDNA) by both the convergent and divergent primers while from gDNA by only the convergent primer (Fig. 1F). We then identified through the circBase website that the circRNA ID of circ_0000811 was mmu_circ_0000811, the genomic length was 26135, and the Position was chr17:71110415-71136549, which came from the Dlgap1 host gene (Supplementary Table 1). Moreover, the results of qRT-PCR then validated the down-regulated expression of circ_0000811 in the MVN of the vertigo mice (Fig. 1G).

Collectively, circ_0000811 was suggested to be lowly expressed in CI-induced vertigo.

### Overexpression of circ_0000811 alleviates experimental CI-induced vertigo in mice

**Fig. 2:**
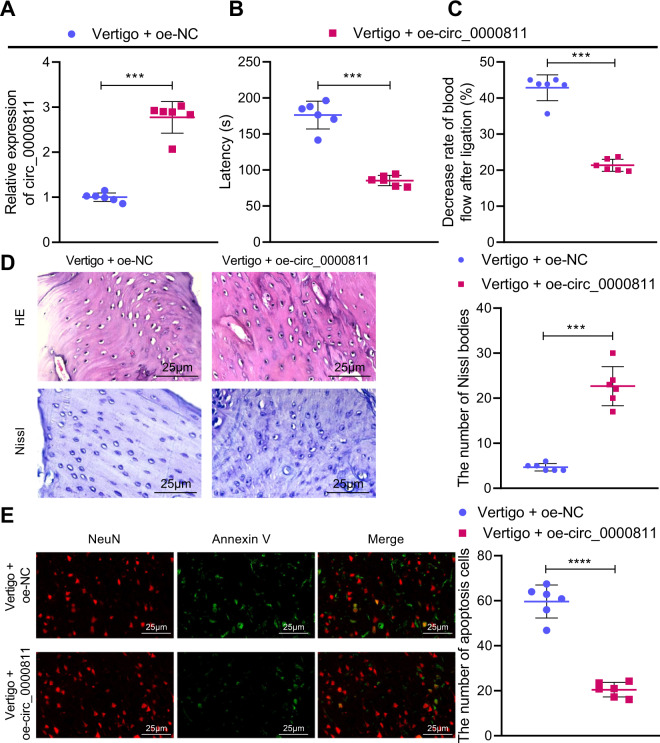
Circ_0000811 overexpression alleviated experimental CI-induced vertigo in mice. **A** qRT-PCR to determine the expression of circ_0000811 in the MVN of mice with CI-induced vertigo in response to circ_0000811 overexpression; **B** the escape latency of mice with CI-induced vertigo in response to circ_0000811 overexpression; **C** the decrease rate of blood flow in the MVN of mice with CI-induced vertigo in response to circ_0000811 overexpression; **D** H&E and Nissl staining to detect the histopathological changes of the MVN of mice with CI-induced vertigo in response to circ_0000811 overexpression; **E** NeuN/Annexin counterstaining to detect the apoptotic rate of neurons in the MVN of mice with CI-induced vertigo in response to circ_0000811 overexpression. ***p < 0.001 compared with the Vertigo + oe-NC group. The independent sample *t* test was applied for the comparison between two groups. CI cerebral ischemia, qRT-PCR quantitative reverse-transcription polymerase chain reaction, MVN medial vestibular nucleus, H&E hematoxylin and eosin; NeuN neuronal nuclei.

After identifying the aberrant expression of circ_0000811, we then explored the effects of circ_0000811 on mice with CI-induced vertigo by manipulating its expression. Mice with CI-induced vertigo overexpressing circ_0000811 were successfully established, as validated by qRT-PCR (Fig. 2A). Notably, prolonged escape latency as well as declines in MVN blood flow and in neuron survival are symbols of the successfully established mouse model of CI-induced vertigo, and the decline in neuron survival could be reflected by decreased number of Nissl bodies [21]. Our data revealed that circ_0000811 overexpression shortened the escape latency and reversed the decrease in the blood flow rate in the MVN of mice with CI-induced vertigo (Fig. 2B, C). Meanwhile, the number of visible Nissl bodies in the neurons in the MVN of mice with CI-induced vertigo was obviously increased in the presence of circ_0000811 restoration (Fig. 2D). The results of NeuN/Annexin counterstaining subsequently indicated a decreased apoptotic rate of neurons in the MVN of circ_0000811 overexpressing-mice with CI-induced vertigo versus that in untreated ones (Fig. 2E). Taken together, our data substantiated that circ_0000811 overexpression may alleviate experimental CI-induced vertigo in mice.

### Circ_0000811 acts as a miR-15b sponge to inhibit its expression

To further explore the downstream targets of circ_0000811, we predicted the downstream miRNAs of circ_0000811 using the Starbase database, and a total of 22 miRNAs (mmu-miR-471-3p, mmu-miR-488-3p, mmu-miR-326-3p, mmu-miR-330-5p, mmu-miR-1247-5p, mmu-miR-322-5p, mmu-miR-103-3p, mmu-miR-107-3p, mmu-miR-6342, mmu-miR-6419, mmu-miR-1955-3p, mmu-miR-15b-5p, mmu-miR-15a-5p, mmu-miR-497a-5p, mmu-miR-195b, mmu- miR-6353, mmu-miR-1907, mmu-miR-16-5p, mmu-miR-542-3p, mmu-miR-195a-5p, mmu-miR-140-5p, and mmu-miR-876-3p) were found to bind to circ_0000811. As shown in Supplementary Fig. 1, a regulation network of circ_0000811 was constructed through the Cytoscape software. Further, qRT-PCR analysis indicated that mmu-miR-103-3p, mmu-miR-107-3p and mmu-miR-15b-5p were highly expressed in the MVN of mice with CI-induced vertigo, among which the expression of miR-15b-5p was the highest (Supplementary Fig. 2 and Supplementary Table 2).

**Fig. 3:**
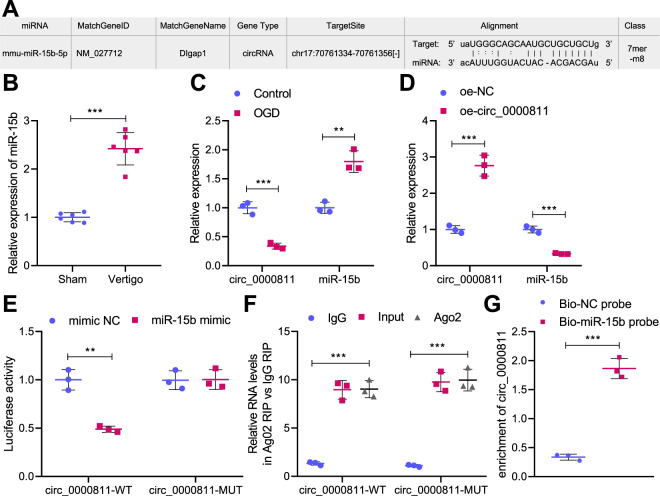
Circ_0000811 acted as a sponge for miR-15b. **A** The binding site between circ_0000811 and miR-15b sequence predicted by the Starbase database; **B** qRT-PCR to determine the expression of miR-15b in the MVN of mice with CI-induced vertigo and sham-operated mice; **C** qRT-PCR to determine the expression of circ_0000811 and miR-15b in the OGD-exposed cell model; **D** qRT-PCR to determine the expression of circ_0000811 and miR-15b in OGD-exposed cells in the presence of oe-circ_0000811; **E** dual luciferase reporter assay to validate the binding affinity between circ_0000811 and miR-15b; **F**, **G** RIP assay (**F**) and RNA pull-down assay (**G**) to verify that circ_0000811 binds to miR-15b. **p < 0.05, ***p < 0.001. Independent sample *t* test was applied for the comparison between two groups, and one-way ANOVA with Tukey’s post hoc test was applied for the comparison among multiple groups. Cell experiments were repeated three times. qRT-PCR quantitative reverse-transcription polymerase chain reaction, MVN medial vestibular nucleus, OGD oxygen glucose deprivation, RIP RNA Immunoprecipitation.

Following that, our bioinformatics analysis then identified the presence of a circ_0000811 binding site on miR-15b (Fig. 3A), and miR-15b, intriguingly, has been reported for its overexpression occurred in middle cerebral artery occlusion [15]. Herein, we speculated that circ_0000811 may affect CI-induced vertigo through miR-15b.

To examine the speculation, we first observed through qRT-PCR the up-regulated expression of miR-15b in the MVN of mice with CI-induced vertigo (Fig. 3B) as well as the OGD-induced cells (Fig. 3C). Meanwhile, the down-regulation of circ_0000811 level was also verified in OGD-exposed cells. Furthermore, the expression level of miR-15b in the cells was repressed in response to circ_0000811 restoration (Fig. 3D). The binding of circ_0000811 to miR-15b was then validated through dual luciferase reporter gene assay (Fig. 3E), and the enrichment of circ_0000811 and miR-15b by Ago2 antibody was found in RIP assay (Fig. 3F). Moreover, circ_0000811 was demonstrated to be pulled down by miR-15b (Fig. 3G).

In summary, these results uncovered that circ_0000811 bound to and reversely regulated miR-15b.

### Circ_0000811 inhibits neuronal apoptosis by regulating miR-15b/Prkar2a

**Fig. 4:**
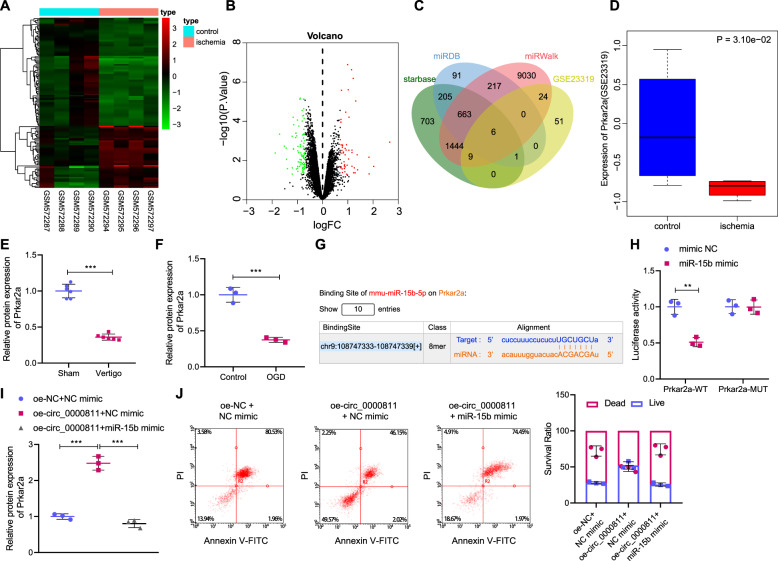
Circ_0000811 overexpression attenuated neuronal apoptosis by regulating the miR-15b/Prkar2a axis. **A**, **B** The heat map (**A**) and volcano plot (**B**) of differentially expressed mRNAs in GSE23319 CI-related dataset; **C** the Venn diagram of the intersection between downstream target genes of miR-15b predicted through Starbase, miRDB and miRWalk databases and down-regulated mRNAs in the GSE23319 dataset; **D** the expression of Prkar2a in GSE23319 dataset (the blue box indicates that in normal samples and the red box indicates that in CI samples); **E** Western blot to measure the expression of Prkar2a in the MVN of mice with CI-induced vertigo and sham-operated mice; **F** Western blot to determine Prkar2a protein level in OGD-exposed cells; **G** predicted binding site of miR-15b to Prkar2a sequence: **H** Dual luciferase reporter gene to validate the binding of miR-15b to Prkar2a; **I** Western blot to determine the Prkar2a protein level in OGD-exposed cells in response to circ_0000811 overexpression alone or in combination with miR-15b overexpression; **J** flow cytometry to detect the apoptosis in OGD-exposed cells in response to circ_0000811 overexpression alone or in combination with miR-15b overexpression. **p < 0.01, ***p < 0.001. Independent sample *t* test was applied for the comparison between two groups, and one-way ANOVA with Tukey’s post hoc test was applied for the comparison among multiple groups. Cell experiments were repeated three times. Prkar2a protein kinase, cAMP dependent regulatory, type II alpha, mRNA messenger RNA, CI cerebral ischemia, MVN medial vestibular nucleus, OGD oxygen glucose deprivation.

Following the circ_0000811/miR-15b axis, we then explored the downstream genes of miR-15b in CI-induced vertigo. The analysis based on GSE23319 CI-related dataset screened out 52 mRNAs with up-regulated expression and 91 ones with down-regulated expression (Fig. 4A, B), which were then intersected with downstream target genes of miR-15b predicted by databases (Starbase, miRDB, miRWalk) to obtain six candidate genes: Prkar2a, Pam, Rab3c, Kpna3, Trim35, and Cpne1 (Fig. 4C). Among them, Prkar2a was observed to be poorly expressed in CI samples from the box plot based on the expression data in the GSE23319 dataset (Fig. 4D). We then performed Western blot and validated the down-regulated protein level of Prkar2a in the MVN of mice with CI-induced vertigo as well as OGD-exposed cells (Fig. 4E, F and Supplementary Fig. 3A, B).

Afterwards, the binding site between miR-15b and Prkar2a sequence was predicted by the Starbase database (Fig. 4G) and the binding affinity between them was demonstrated by the dual luciferase assay (Fig. 4H). Next, we performed in vitro gain- and loss- of function assays by manipulating the expression of circ_0000811 and miR-15b in OGD-exposed cells. According to the results (Fig. 4I, J and Supplementary Fig. 3C), Prkar2a level was increased in response to circ_0000811 overexpression alone, whereas additional up-regulation of miR-15b could abrogate the increase in Prkar2a level; similarly, the suppressive effects on neuronal apoptosis of circ_0000811 overexpression alone was reversed when it was combined with miR-15b overexpression.

Collectively, our data unraveled that circ_0000811 augmented the expression of Prkar2a by sponging miR-15b, thereby attenuating neuronal apoptosis.

### Prkar2a represses neuronal apoptosis via inhibition of the JAK2/STAT1 axis

**Fig. 5:**
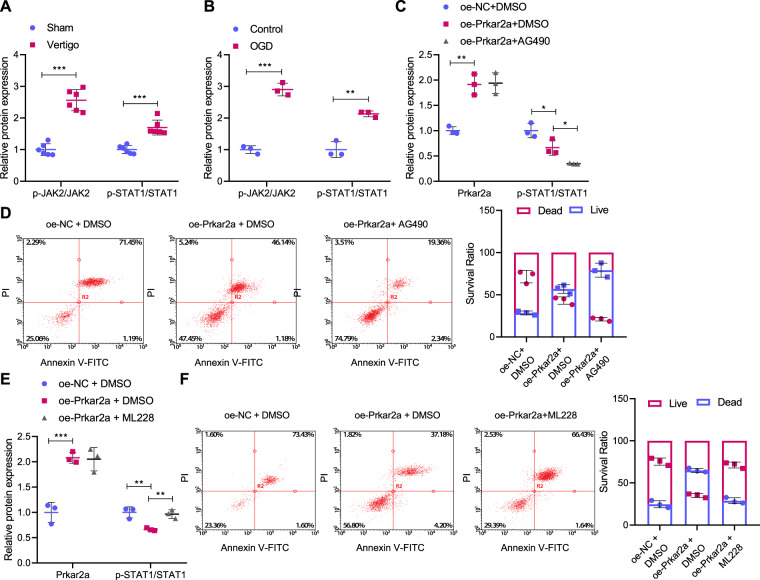
Prkar2a suppressed neuronal apoptosis by inactivating the JAK2/STAT1 axis. **A** Western blot to determine the phosphorylation level of JAK2 and STAT1 in the MVN of mice with CI-induced vertigo and sham-operated mice; **B** Western blot to determine the phosphorylation level of JAK2 and STAT1 in OGD-exposed cells; **C** Western blot to determine the expression of Prkar2a and the phosphorylation level of STAT1 in OGD-exposed cells overexpressing Prkar2a with/without AG490 treatment; **D** flow cytometry to detect the apoptosis in OGD-exposed cells overexpressing Prkar2a with/without AG490 treatment; **E** Western blot to determine the expression of Prkar2a protein and the phosphorylation level of STAT1 in OGD-exposed cells overexpressing Prkar2a with/without ML228 treatment; **F** flow cytometry to detect the apoptotic rate in OGD-exposed cells overexpressing Prkar2a with/without ML228 treatment. *p < 0.05, **p < 0.01, ***p < 0.001. Independent sample *t* test was applied for the comparison between two groups, and one-way ANOVA with Tukey’s post hoc test was applied for the comparison among multiple groups. Cell experiments were repeated three times. Prkar2a protein kinase, cAMP dependent regulatory, type II alpha, JAK2 Janus kinase 2, STAT1 signal transducer and activator of transcription 1, MVN medial vestibular nucleus, OGD oxygen glucose deprivation, AG490 JAK2/STAT1 signaling pathway inhibitor, ML228 JAK2/STAT1 signaling pathway agonist.

Since it has been suggested that Prkar2a may reversely regulate the JAK2-STAT1 axis [17], we further examined whether Prkar2a repressed neuronal apoptosis through the JAK2/STAT1 axis. First, we observed that the phosphorylation levels of JAK2 and STAT1 were increased in the MVN of mice with CI-induced vertigo (Fig. 5A, B and Supplementary Fig. 3D, E) as well as in OGD-exposed cells (Fig. 5C and Supplementary Fig. 3F). We then employed AG490, an inhibitor against JAK2-STAT1 signaling pathway, to treat the OGD-exposed cells. The results revealed that Prkar2a overexpression alone led to reduced STAT1 phosphorylation level, and additional AG490 treatment further reduced STAT1 phosphorylation level while showed no effects on Prkar2a protein level (Fig. 5C and Supplementary Fig. 3F). Similarly, the suppressive effect on the apoptotic rate in cells overexpressing Prkar2a alone was intensified when it was combined with AG490 (Fig. 5D).

In addition, Prkar2a overexpression OGD-exposed cells were treated with the JAK2-STAT1 signaling pathway activator ML228. According to Western blot results, the Prkar2a protein level was increased and the phosphorylation level of STAT1 was reduced in cells treated with oe-Prkar2a alone; and additional ML228 treatment led to up-regulated phosphorylation level of STAT1 while showed no effect on Prkar2a protein level (Fig. 5E and Supplementary Fig. 3G). Results of flow cytometry then indicated that the decline of apoptotic rate induced by oe-Prkar2a + DMSO treatment could be reversed by ML228 treatment (Fig. 5F).

Taken together, Prkar2a may attenuate neuronal apoptosis by repressing the JAK2/STAT1 axis in OGD-exposed cells.

### Circ_0000811 ameliorates CI-induced vertigo through the miR-15b/Prkar2a/JAK2/STAT1 axis

**Fig. 6:**
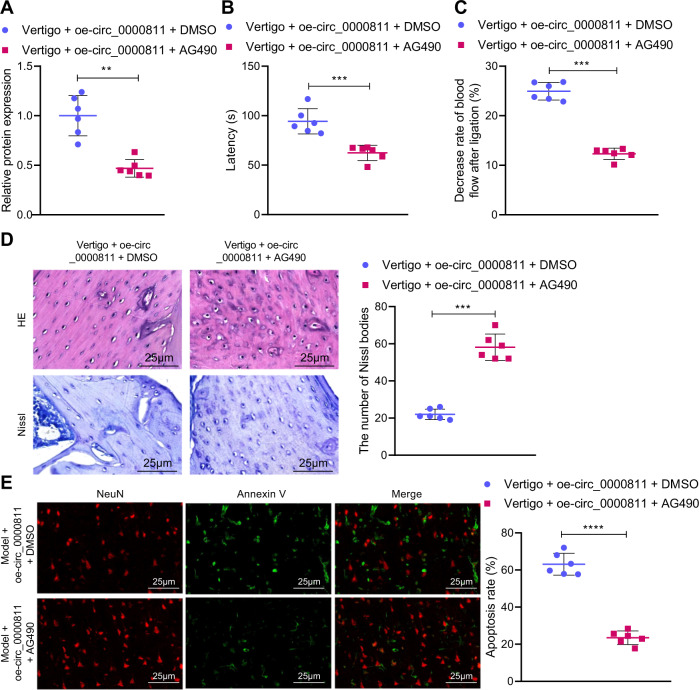
Circ_0000811 overexpression ameliorated experimental CI-induced vertigo through the miR-15b/Prkar2a/JAK2/STAT1 axis. **A** Western blot to determine the phosphorylation level of STAT1 in the MVN of mice with CI-induced vertigo overexpressing circ_0000811 with/without AG490 treatment; **B** the escape latency of mice with CI-induced vertigo overexpressing circ_0000811 with/without AG490 treatment; **C** the blood flow of the MVN after ligation of CCA and SCA in mice with CI-induced vertigo overexpressing circ_0000811 with/without AG490 treatment; **D** H&E and Nissl staining to detect the histopathological changes of the MVN in mice with CI-induced vertigo overexpressing circ_0000811 with/without AG490 treatment; **E** NeuN/Annexin counterstaining staining to detect the neuronal apoptosis in the MVN of mice with CI-induced vertigo overexpressing circ_0000811 with/without AG490 treatment. **p < 0.01, ***p < 0.001 compared with the Vertigo + oe-circ_0000811 + DMSO group. Independent sample *t* test was applied for the comparison between two groups. CI cerebral ischemia; Prkar2a, protein kinase, cAMP dependent regulatory, type II alpha, JAK2 Janus kinase 2, STAT1 signal transducer and activator of transcription 1, MVN medial vestibular nucleus, CCA common carotid artery, SCA superior cerebellar artery, H&E hematoxylin and eosin, NeuN neuronal nuclei, AG490 JAK2/STAT1 signaling pathway inhibitor.

To substantiate the circ_0000811/miR-15b/Prkar2a/JAK2/STAT1 regulatory axis in vivo, we performed a series of gain- and loss- of functions assays on mice with experimental CI-induced vertigo. Consistent with the aforementioned in vitro results, the in vivo level of STAT1 phosphorylation in the mice with CI-induced vertigo that was reduced by circ_0000811 overexpression alone was further decreased by the combination of circ_0000811 overexpression and AG490 treatment (Fig. 6A). Meanwhile, versus circ_0000811 restoration alone, its combination with AG490 resulted in shortened escape latency as well as improved blood flow, increased visible Nissl bodies in neurons, and reduced neuronal apoptosis in the MVN of the mice with CI-induced vertigo (Fig. 6B–E). In other words, additional AG490-induced inhibition of the JAK2/STAT1 axis strengthened the effects of circ_0000811 restoration alone. Combining these results with the aforementioned validation of the circ_0000811/miR-15b/Prkar2a and Prkar2a/JAK2/STAT1 mediatory axes, our data elucidated that circ_0000811 could ameliorate CI-induced vertigo through modulating the miR-15b/Prkar2a/JAK2/STAT1 axis in mice.

## Discussion

CI-caused neuron death may lead to lifelong neurological deficits and frequent symptoms such as vertigo [22]. However, there still lacks effective treatment targeting CI-induced ischemia. Accumulating evidence has recognized circRNAs as a novel class of biomarkers for a variety of diseases, including cardiovascular diseases [23, 24]. Herein, we examined the theoretical feasibility of circRNA therapeutics for the management of experimental CI-induced vertigo via affecting neuronal apoptosis.

Initially, we developed CI mice through ligation of common carotid artery (CCA) and superior cerebellar artery (SCA) and identified CI-induced vertigo through detection of escape latency and decrease rate in blood flow. Notably, escape latency and decrease rate in blood flow have been employed as critical indexes for the establishment of animal models of vertigo or vessel occlusion in a number of previous studies [25, 26]. Vertigo refers to an illusion of movement due to the asymmetric stimulation of the vestibular system [27]. Thus, we performed circRNA sequencing on mice with CI-induced vertigo, and circ_0000811, a circRNA that has been rarely reported before, stood out for its obviously down-regulated expression in the brain tissue. Furthermore, we validated the poor expression of circ_0000811 in mouse hippocampus neuron HT22 cells exposed to OGD and revealed in vivo that circ_0000811 overexpression alleviated CI-induced vertigo. Although circ_0000811 has not been noted, several other circRNAs have been reported to be correlated with ischemic injury. For instance, Chang et. al. indicated that circ-100338 induced angiogenesis following myocardial ischemia-reperfusion injury [28], and the down-regulation of circFndc3b has been found in the cardiac tissue of patients with ischemic cardiomyopathy [29].

Afterwards, we explored the downstream target of circ_0000811 and demonstrated that circ_0000811 acted as a sponge for miR-15b, and we validated both in vitro and in vivo the overexpression of miR-15b in CI-related vertigo. In agreement with our findings, a previous study indicated miR-15b to be a contributor of cerebral ischemic injury through suppressing Bcl-2 [15], and repressed miR-15b by Kaempferol has been reported for alleviating OGD-induced ischemic injury in neurons [30]. Another circRNA, Ttc3, has also been uncovered to modulate the myocardial infarction-induced injury by sponging miR-15b [31]. Moreover, accumulating evidence has indicated the regulatory role of miRNAs in neurological diseases, especially in ischemic-hypoxic encephalopathy [32, 33]. For instance, a prior report has highlighted the pathophysiological role of reperfusion-inducible miR-1264/1298/448 cluster in a mouse model of ischemic stroke [34]; another study revealed that genetic deletion of the miR-15a/16-1 cluster could stimulate angiogenesis and neurological recovery in ischemic stroke *via* the Src signaling pathway [35]. In the present study, we verified the circ_0000811/miR-15b regulatory axis in CI-induced vertigo and thus provided supplementary theoretical basis for the critical role of miRNAs in neurological diseases.

We then identified Prkar2a to be the target gene of miR-15b and substantiated through a functional assay that circ_0000811 restoration augmented the expression of Prkar2a by sponging miR-15b, thereby attenuating neuronal apoptosis. Prkar2a is a regulatory subunit of Protein Kinase A (PKA), the main modulator of cyclic adenosine mono-phosphate-dependent signaling pathways [36]. Intriguingly, PKA-related signaling pathways have been reported for neuroendocrine functions through postsynaptic mediation of PKA activity [37, 38]. Furthermore, our data revealed that Prkar2a inactivated the JAK2/STAT1 signaling pathway, thus attenuating neuronal apoptosis in CI-related vertigo. It corroborates a prior study where Prkar2a has been indicated to reversely regulate the JAK2/STAT1 signaling pathway [17]. Notably, the activation of the JAK2/STAT1 signaling pathway has been reported to be involved in cell apoptosis [19, 39]. Importantly, inactivated JAK2/STAT1 due to GJ-4 was found to improve memory impairment in focal CI/reperfusion in rats [20].

Following that, we then performed a series of in vivo gain- and loss-of function assays. The results unraveled that the AG490-induced inhibition of the JAK2/STAT1 signaling pathway alleviated CI-induced vertigo through repression of neuronal apoptosis, its combination with circ_0000811 strengthening the effects of circ_0000811 overexpression alone. Hence, circ_0000811 may mitigate CI-induced vertigo through suppressing the JAK2/STAT1 signaling pathway to attenuate neuronal apoptosis. In relation to our finding, Hydroxysafflor Yellow A has also been reported to ameliorate myocardial ischemic injury *via* inhibiting the activation of the JAK2/STAT1 signaling pathway [19]. Nonetheless, in the present study we predicted the potential miRNAs targeted by circ_0000811 only through online tools in bioinformatics analysis, which exists as one of the limitations of this study. Future investigations are merited to examine the potential correlation between those candidate mRNAs with up-regulated expression and the OGD/R injury of neuronal cells.

**Fig. 7:**
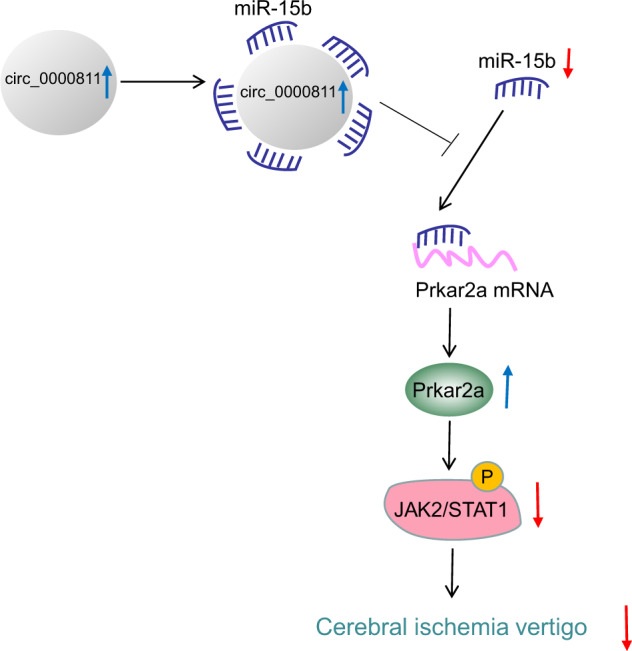
The mechanism graph of the regulatory role of circ_0000811 in cerebral ischemic vertigo. Circ_0000811 upregulates Prkar2a and inactivates the JAK2/STAT1 axis through competitively binding to miR-15b and thus ameliorates cerebral ischemic vertigo.

Taken together, the data acquired in the present study elucidated the mechanism by which circ_0000811 overexpression ameliorated CI-induced vertigo through attenuating the apoptosis of neurons *via* the modulation of the miR-15b/Prkar2a/JAK2/STAT1 cascade. Circ_0000811 sponged miR-15b to inhibit its expression and augment Prkar2a expression, whereby the JAK2/STAT1 signaling pathway was repressed, and the apoptosis of neurons was thus attenuated to mitigate the CI-induced vertigo (Fig. 7). By establishing the regulatory mechanism and molecular network underlying the mitigative effects of circ_0000811 on CI-induced vertigo, our study deepens our understanding of the etiology of CI-induced vertigo and provides potential therapeutic targets for the development of novel treatment for CI-induced vertigo. However, whether Prkar2a played a neuroprotective role in CI-induced vertigo through other downstream signal pathways remains unclear with further work required to deepen understanding. Moreover, in our study, only two pairs of brain tissue samples from normal mice and mice with CI-induced vertigo were selected for circRNA sequencing. Although the reliability of the sequencing results was verified by subsequent qRT-PCR, the small size of sequencing samples might affect the reliability of the results to a certain extent. Therefore, future studies with larger sample sizes are also recommended.

## Materials and methods

### Ethics statement

Animal experiments were approved by the Animal Care and Use Committee of Shengjing Hospital of China Medical University and performed in accordance with *Guide for the Care and Use of Laboratory Animals* published by the National Institutes of Health.

### Establishment of a mouse model of CI-induced vertigo

A total of 76 healthy male C57BL/6J mice (aged 6 weeks, weighing 18 g) were housed with free access to food and water under specific pathogen-free (SPF) conditions. The mice were fed under 12-h light/dark cycles with 60–65% humidity at temperature of 22–25 °C. The health status of the mice was examined before the experiment. After acclimatization, the mice were randomly divided into the following six groups: sham-operated mice (*n* = 14), mice with experimental CI-induced vertigo without further treatment (*n* = 14), or mice with CI-induced vertigo treated with oe-negative control (NC) (intracerebroventricular injection of lentiviral vectors carrying NC, *n* = 12), oe-circ_0000811 (intracerebroventricular injection of lentivirus carrying circ_0000811 overexpression plasmids, *n* = 12), oe-circ_0000811 + AG490 (circ_0000811 overexpression vectors + AG490, a JAK2/STAT1 pathway inhibitor, *n* = 12), or oe-circ_0000811 + DMSO (circ_0000811 overexpression vectors + DMSO, *n* = 12). The dose of lentivirus injection was 1 × 10^8^ TU, and the volume of intraventricular injection was no more than 2 μL.

For surgery, the mice were anesthetized and fixed in supine position. Next, a cut was made on the neck skin to expose and separate the right CCA, which was then threaded and ligated. Specifically, 7-0 silk suture was used to ligate the distal end of the CCA 2 mm away from the bifurcation, and another 7-0 silk suture was inserted from the external carotid artery (ECA) and a slipknot was tied near the CCA bifurcation. Subsequently, the right SCA was identified by tracing the right CCA into the thoracic cavity and then hooked out with curved forceps for threading and ligating. A small opening was cut in the ECA 1.5 mm away from the CCA bifurcation, and a 0.18-mm diameter nylon suture was then inserted through the opening into the internal carotid artery and inwardly into the middle cerebral artery (the insertion depth was about 9 ± 1 mm from the CCA bifurcation) [40].

Next, 60 min after the inducement of ischemia, sutures were removed, the proximal end of the ECA was ligated with 7-0 silk suture, the neck wound was sutured with 5-0 silk thread, and the wound was disinfected with vital iodine. Mouse models were then placed on a heating pad under a constant temperature after being awake. Next, 24 h after the operation, the mice were scored for neurological function and subsequently euthanized to collect brain tissues. Six mice were selected from each group for TTC staining (Supplementary Fig. 4). Notably, the CCA and SCA of sham-operated mice were stripped without ligating. After the surgery, escape latency was measured and changes in the blood flow of the medial vestibular nucleus (MVN) was detected to identify whether the mouse model of experimental cerebral ischemic vertigo was successfully established.

### TCC staining

The brain tissues of mice in each group were cut into 2-mm sections. The sections were stained with TTC (2%) at 37 °C for 0.5 h, and then fixed with 4% paraformaldehyde at 4 °C overnight. Finally, the sections were photographed to observe the size of cerebral infarction.

### Measurement of escape latency

To validate the occurrence of CI-induced vertigo in the mouse models, we measured the escape latency [25], namely the time required for the mice to regain balance (and keep balance for at least 30 s) on a platform following a combined stimulation by rotation (500 r/min, 30 s) and electricity. Before the test, the mice were placed on a platform for 3-min adaption and then continuously given electrical stimulation (30 V, 50 Hz) for 5 min, and mice that jumped on the platform and stayed for more than 30 s were considered successfully trained. The training was performed twice a day for 3 consecutive days to establish a strong conditioned reflex.

### Sequencing of circRNA

Cerebral tissues from the aforementioned mice with CI-induced vertigo (*n* = 2) and normal mouse cerebral tissues (*n* = 2) were collected for circRNA sequencing. Total RNA was extracted from the tissues and reversely transcribed into cDNA after removal of ribosomal RNA (rRNA) and linear RNA. Then, NextSeq CN500 sequencer was used for circRNA sequencing, vcl2fastq2 software was applied for base recognition, and the data were converted into original reads. After that, FastQC, cutadapt, bbmap and other software were employed for data quality control. The reference genome and annotation files were retrieved from the UCSC database, comparison with the reference genome was conducted with bwa software, and CIRI software was used for circRNA identification. Moreover, circBase (http://circbase.org/) was utilized for sequence annotation.

### Bioinformatics analysis

The original count of circRNA identified in each sample was normalized by the Voom method in the Limma package in R language, with |logFC | >3 and *p* < 0.01 as the threshold for screening of differentially expressed circRNAs related to cerebral ischemic vertigo. Then, the Starbase database was used to predict the downstream miRNAs of the candidate circRNAs, which was combined with qRT-PCR analysis to identify candidate miRNAs. The Cytoscape software was then utilized to draw the regulation network of circ_0000811, followed by prediction of the downstream gene of the obtained miRNAs utilizing Starbase, miRDB, and miRWalk databases. Next, a cerebral ischemia injury-related mRNA expression dataset GSE23319, containing 4 cerebral tissue samples with ischemic injury (striatum-HI-24h) and 4 control samples (striatum-Sham-24h), was retrieved from the GEO database, based on which differentially expressed mRNAs were identified with |logFC | >0.7 and *p* < 0.05 as the screening threshold. Identified miRNAs were then intersected with the aforementioned candidate genes predicted by databases.

### Blood flow detection

After the surgery, the mice were fixed on a three-dimensional brain stereotaxic device. The MVN on the right side was identified at the position 10.2 mm from the anterior bregma, 1.1 mm to 2.5 mm from the right side of the suture, and 4.5 mm to 5.5 mm in depth. Then, the blood flow was detected using a laser Doppler blood flow meter (the fiberoptic probe went deeper into 0.5-1 mm below for non-contact detection) with the cerebral blood flow curve drawn. Upon the blood becoming stable, the right CCA and SCA were ligated, followed by another 30-min supervision of the blood flow. The average blood flow value (of 1 min) at time points of 5, 10, 15, 20, 25, 30 min before or after the ligation was recorded.

### Hematoxylin and eosin (H&E) staining

H&E staining was conducted as previously described to observe the pathological changes of mouse MVN [41]. Briefly, paraffin-embedded slides of MVN were dewaxed and dehydrated, and subsequently stained by hematoxylin for 3-5 min and eosin for 5 min, followed by microscopy observation. Photographed images were analyzed using the Image J software.

### Development of in vitro oxygen and glucose deprivation (OGD) cell model

OGD model was developed using mouse hippocampus neuron HT22 cells (iCell-m020, iCell Bioscience, Shanghai, China) to stimulate in vitro cerebral ischemic and hypoxic microenvironment. Glucose-free Dulbecco’s modified Eagle medium (DMEM) was incubated in an OGD-specific incubator containing 5% CO_2_, 94% N_2_, and 1% O_2_ (Thermo Fisher Scientific, Waltham, MA, USA) for 4 h. Subsequently, the cells that had been cultured under normoxic conditions were further incubated with the glucose-free DMEM in the OGD-specific incubator (5% CO_2_, 94% N_2_, and 1% O_2_) for 12 h.

### Cell grouping and treatment

The cells were randomly divided into 11 groups, including a control group and 10 groups for OGD models. A group of the OGD-exposed cells underwent no further treatment (signed as the OGD group), while other 9 groups of OGD-exposed cells were then transfected with circ_0000811 overexpression plasmids (oe-circ_0000811) alone or in combination with plasmids containing miR-15b mimic or corresponding NC, signed as oe-circ_0000811, oe-NC, oe-circ_0000811 + miR-15b mimic, oe-circ_0000811 + NC mimic, and oe-NC + NC mimic groups; or transfected with Prkar2a overexpression plasmids followed by treatment of AG490 (an inhibitor against the JAK2/STAT1 signaling pathway) or ML228 (a JAK2/STAT1 signaling pathway activator) or DMSO as the control, signed as oe-Prkar2a + AG490, oe-Prkar2a + ML228, oe-Prkar2a + DMSO, and oe-NC + DMSO groups.

The circRNA overexpression vector was constructed by cloning circ_0000811 into the EcoRI/EcoRV and EcoRV/XhoI sites of pcDNA3. Firstly, the vector was digested with multiple enzymes, where the upstream digestion used EcoRI/EcoRV enzymes and the downstream digestion used EcoRV/XhoI enzymes. Afterwards, the target fragments were amplified by designing Divergent primers, which were connected to the target vector using T4 ligase. The successfully ligated plasmids were transformed and amplified using *Escherichia coli*, and the circ_0000811 overexpression plasmid was extracted.

Further, cell transfection was performed utilizing Lipofectamine 2000 reagent (Invitrogen, Carlsbad, CA, USA) following the manufacturer’s protocols. The miRNA mimic plasmid was purchased from GenePharma Co., Ltd. (Shanghai, China), and 4 μg of the target plasmid along with 10 μL of Lipofectamine 2000 reagent was diluted with 250 μL of serum-free Opti-MEM medium (Gibco, Gaithersburg, MD, USA) and allowed to stand at room temperature for 5 min. The mixture was then cultured for 6 h in plates in a 5% CO_2_ incubator at 37 °C, followed by 48-h incubation with the complete medium. After that, the cells were collected to measure the transfection efficiency for subsequent experiments.

### Lentivirus packaging and transduction

The lentiviral backbone vector pLenti was subjected to double-enzyme digestion, and the target gene fragment was, at the same time, added with a double-enzyme digestion site. In this way, the target gene was inserted to the pLenti vector, and the constructed vector was then transferred to the competent DH5α cells for amplification in *E. coli*. After sequencing, the lentiviral vectors carrying the plasmid expressing the target gene were extracted. Moreover, well-grown 293 T cells (Procell, Wuhan, China) were spread on a 10 cm dish, followed by addition of lentiviral target gene (3 μg), pCMV-VSV-G (1 μg), and pCMV-Delta8.9 (3 μg) to each well, with the Lipofectamine 3000 reagent (Invitrogen) utilized to enhance the transduction efficiency. Twenty-four hours later, the medium was renewed with 12 ml of fresh medium containing 5% fetal bovine serum for another 48-h incubation. Afterwards, the cell supernatant containing lentivirus was collected, filtered with a 0.45 um, and stored at −80 °C.

### Nissl staining

The tissues were fixed with 4% paraformaldehyde, prepared into paraffin sections, dewaxed in xylene, rehydrated with graded ethanol (95, 85, and 75%), and stained with Nissl staining solution (C0117, Beyotime, Shanghai, China) for 3–10 min at 37–50 °C. The sections were dehydrated with 95% ethanol and permeabilized with xylene. The sections were mounted with neutral gum or another mounting medium, observed under a microscope, and photographed.

### Circular structure assay

Trizol (Invitrogen, Carlsbad, CA, USA) was adopted to extract total RNA from MVN, and the concentration and purity of the extracted RNA were detected by a nanodrop2000 micro ultraviolet spectrophotometer (1011U, nanodrop, USA). Next, 2 μg total RNA was added with 3 U/μg RNase R (Epicenter Technologies, Madison, WI, USA) and incubated at 37 °C for 30 min. RNeasy MinElute Cleaning kits (Qiagen GmbH, Hilden, Germany) were used for purification. The expression of circ_0000811 was detected after reverse transcription to obtain cDNA and observed by agarose gel electrophoresis. Genomic DNA (gDNA) of MVN was extracted with MiniBEST Universal Genomic DNA Extraction Kit Ver.5.0 (Takara, Japan). Based on the designed convergent polymer primers and divergent primers, circ_0000811 was detected in cDNA and gDNA and observed by agarose gel electrophoresis.

### RNA extraction and quantitative reverse-transcription polymerase chain reaction (qRT-PCR)

Total RNA was extracted from MVN or cells utilizing TRIzol reagent (Invitrogen, Carlsbad, CA, USA), followed by determination of RNA concentration and purity with a Nanodrop2000 micro ultraviolet spectrophotometer (1011U, NanoDrop Technologies, Wilmington, DE, USA). Then, RNA was reversely transcribed to cDNA according to the protocols of TaqMan MicroRNA Assays Reverse Transcription primer (4427975, Applied Biosystems, Foster City, CA, USA)/PrimeScript RT reagent Kit (RR047A, Takara, Shiga, Japan). Afterwards, ABI7500 PCR system (ABI, Foster City, CA, USA) was employed for qRT-PCR assay. Primers for circ_0000811 and miR-15b were designed and synthesized by TaKaRa, as listed in Supplementary Table 3. Additionally, GAPDH served as the housekeeper gene for circ_0000811 and U6 for miR-15b. The relative quantification method (2^−△△CT^ method) was used to calculate the relative transcription level (normalized to GAPDH or U6) of target genes.

### Western blot assay

Total protein in MVN or cells was extracted with PMSF-containing RIPA lysis buffer (P0013C, Beyotime), incubated on ice for 30 min, and centrifuged (4 °C, 8000 × *g*) for 10 min, followed by determination of protein concentration utilizing BCA detection kits. A total of 50 μg protein dissolved in 2× SDS loading buffer was boiled (100 °C, 5 min), separated by 10% sodium dodecyl sulfate polyacrylamide gel electrophoresis (SDS-PAGE), electro-transferred to polyvinylidene fluoride (PVDF) membrane, and blocked with 5% skim milk powder for 1 h to suppress non-specific binding. Subsequently, the membrane was incubated overnight at 4 °C with diluted rabbit primary antibodies, including anti-Prkar2a (1:1000, PA5-90367, Invitrogen, Waltham, MA, USA), anti-JAK2 (1:5000, ab108596, Abcam, Cambridge, UK), anti-p-JAK2 (1:4000, Ab32101, Abcam), anti-STAT1 (1:1500, ab92506, Abcam) and anti-p-STAT1 (1:800, #9167, Cell Signaling Technologies, Danvers, MA, USA), and anti-GAPDH (ab9485, 1:2500, Abcam). The membrane was then incubated for 1 h with horseradish peroxidase (HRP)-labeled goat anti-rabbit IgG secondary antibody (ab97051, 1:2000, Abcam), followed by visualization of the protein bands utilizing enhanced chemiluminescence (ECL) fluorescence detection kit (BB-3501, Amersham, Little Chalfont, Buckinghamshire, UK). The Bio-Rad image analysis system (BIO-RAD, Hercules, CA, USA) was applied for photography and the images were analyzed with the Quantity One v4.6.2 software. Further, the gray level of protein bands was quantified and normalized to GAPDH.

### Dual-luciferase reporter gene assay

The potential miR-15b binding site sequence in circ_0000811 promoter (or Prkar2a binding site sequence in miR-15b) and corresponding mutated sequences were cloned into the pGLO vector (Promega, Madison, WI, USA) to construct pGLO-circ_0000811-wildtype (WT) and PGLO-circ_0000811-mutant (MUT) reporter plasmids (or PGLO-miR-15b-WT and PGLO-miR-15b-MUT plasmids). The two reporter plasmids were co-transfected into 293T cells. Besides, the Renilla luciferase plasmid was transfected as a control. The luciferase activity was measured using the dual luciferase reporter gene detection system (Promega). The relative luciferase activity was normalized by calculating the ratio of the firefly luciferase activity to that of Renilla luciferase.

### RNA pull-down

Treated with 50 nM biotin-labeled Bio-miR-15b-probe and Bio-NC-probe (GeneCreate Biological Engineering, Wuhan, Hubei, China) for 48 h, the cells were then collected and incubated with Pierce IP Lysis Buffer (Thermo Scientific) on ice for 30 min. The cell lysate was collected after centrifugation and incubated overnight at 4 °C with an equal amount of streptavidin magnetic beads (Thermo Scientific) under rotation. The precipitate was collected by centrifugation the next day, eluted with high-salt buffer, and centrifuged to collect the supernatant. The RNA that bound to miR-15b was isolated and purified by Trizol method, and the enrichment of circ_0000811 was detected by qRT-PCR.

### RNA immunoprecipitation (RIP) assay

Cells washed with pre-cooled PBS were lysed with an equal volume of RIP lysate (P0013B, Beyotime) on ice for 30 min, followed by 10-min centrifugation at 14,000 rpm, 4 °C to collect the supernatant. Then, RIP kits (Millipore, Billerica, MA, USA) were used to detect the binding of circ_0000811/miR-15b to Ago2 protein. Briefly, the magnetic beads from co-precipitation reaction system were resuspended in RIP Wash Buffer and incubated at 4 °C with rabbit anti-Ago2 antibody (ab186733, 1:50, Abcam, Shanghai) for 6 h. After washing, the magnetic bead-antibody complex was resuspended in 900 μL RIP Wash Buffer and incubated with 100 μL of cell supernatant at 4 °C overnight. Following digestion with proteinase K, the RNA was extracted for subsequent PCR detection. Rabbit anti-IgG antibody (ab172730, 1:100, Abcam) was utilized as a NC.

### Flow cytometry

Annexin V-FITC/PI apoptosis detection kits (C1062M, Beyotime) were used to detect cell apoptosis. Briefly, 1 × 10^6^ cells/ml were resuspended with 195 μL Annexin V-FITC binding solution and then incubated with 5 μL Annexin V-FITC and 10 μL PI in darkness at room temperature for 15 min. Finally, apoptosis was quantified using a flow cytometer (FACSVerse/Calibur/AriaIISORP, BD Biosciences, Franklin Lakes, NJ, USA).

### Immunofluorescence staining

MVN tissue sections of mice were baked at 60 °C for 20 min, soaked in xylene for 15 min, and dehydrated with graded ethanol (100, 95, 90, 85, and 80%). After that, each section was dripped with 3% H_2_O_2_ and soaked at room temperature for 10 min to block endogenous peroxidase, followed by antigen repair for 10 min. The samples were incubated with normal goat serum blocking solution (Sangon, Shanghai, China) at room temperature for 20 min and then with primary antibodies against NeuN (ab207279, 1:500, Abcam) and Annexin (ab108194, 1:50, Abcam) overnight at 4 °C. Cells were incubated with Alexa-Fluor488 or Alexa-Fluor594 coupled secondary antibodies (Invitrogen), and the nuclei were stained with 0.1 µg/ml DAPI. The cells were analyzed using an Olympus BX61 microscope equipped with an F-View II CCD camera. In each experiment, images were acquired using constant camera settings and analyzed via the Cell P (Ommpus Soft Imaging Solutions) program. At least 15 visual fields were randomly selected for each sample.

### Statistical analysis

Data in this study were processed utilizing the SPSS v.24.0 (IBM, Armonk, NY, USA) software. Measurement data were summarized as mean ± standard deviation. Independent sample *t* test was applied for comparison between data of two groups and one-way analysis of variance (ANOVA) with Tukey’s post hoc test was performed for comparison among data of multiple groups. Moreover, *p* < 0.05 indicated statistically significant difference.

## Supplementary information

Supplementary Figures

Supplementary Table 1

Supplementary Table 2

Supplementary Table 3

Original gel blot images

## Data Availability

The data that supports the findings of this study are available on request from the corresponding author upon reasonable request.
