# Supplementary material for: Circ_0000811 acts as a miR-15b sponge and inhibits Prkar2a-mediated JAK2/STAT1 pathway to attenuate cerebral ischemic vertigo
Source: Cell Death Discov. 2022 May 4;8:247. doi: 10.1038/s41420-022-01016-2 (PMC9068921; doi:10.1038/s41420-022-01016-2)
Supplement: Supplementary file 1 — Supplementary Figures [file 41420_2022_1016_MOESM1_ESM.docx]

**
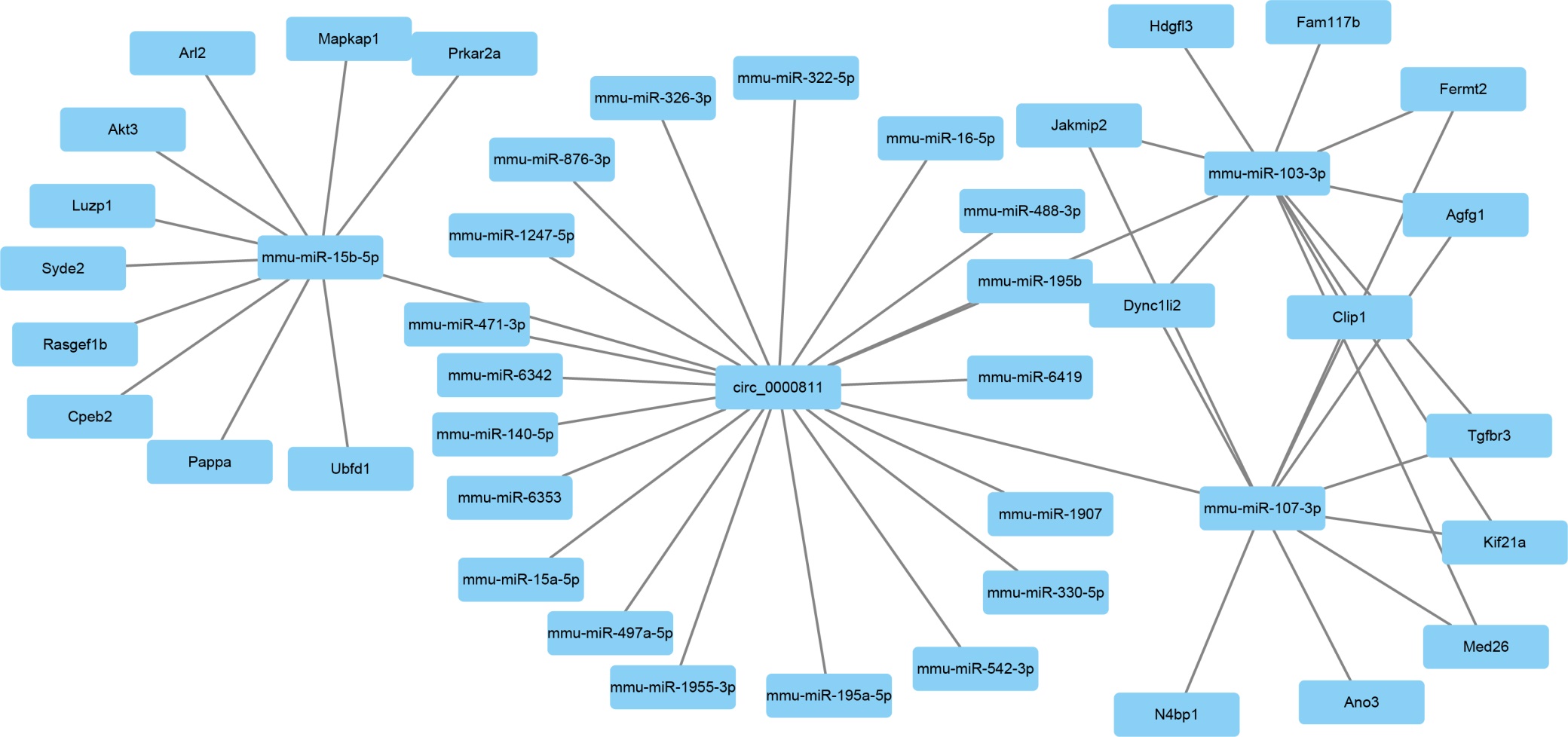
**

**Supplementary Fig. 1** The regulation network of circ_0000811 constructed through the Cytoscape software.

**
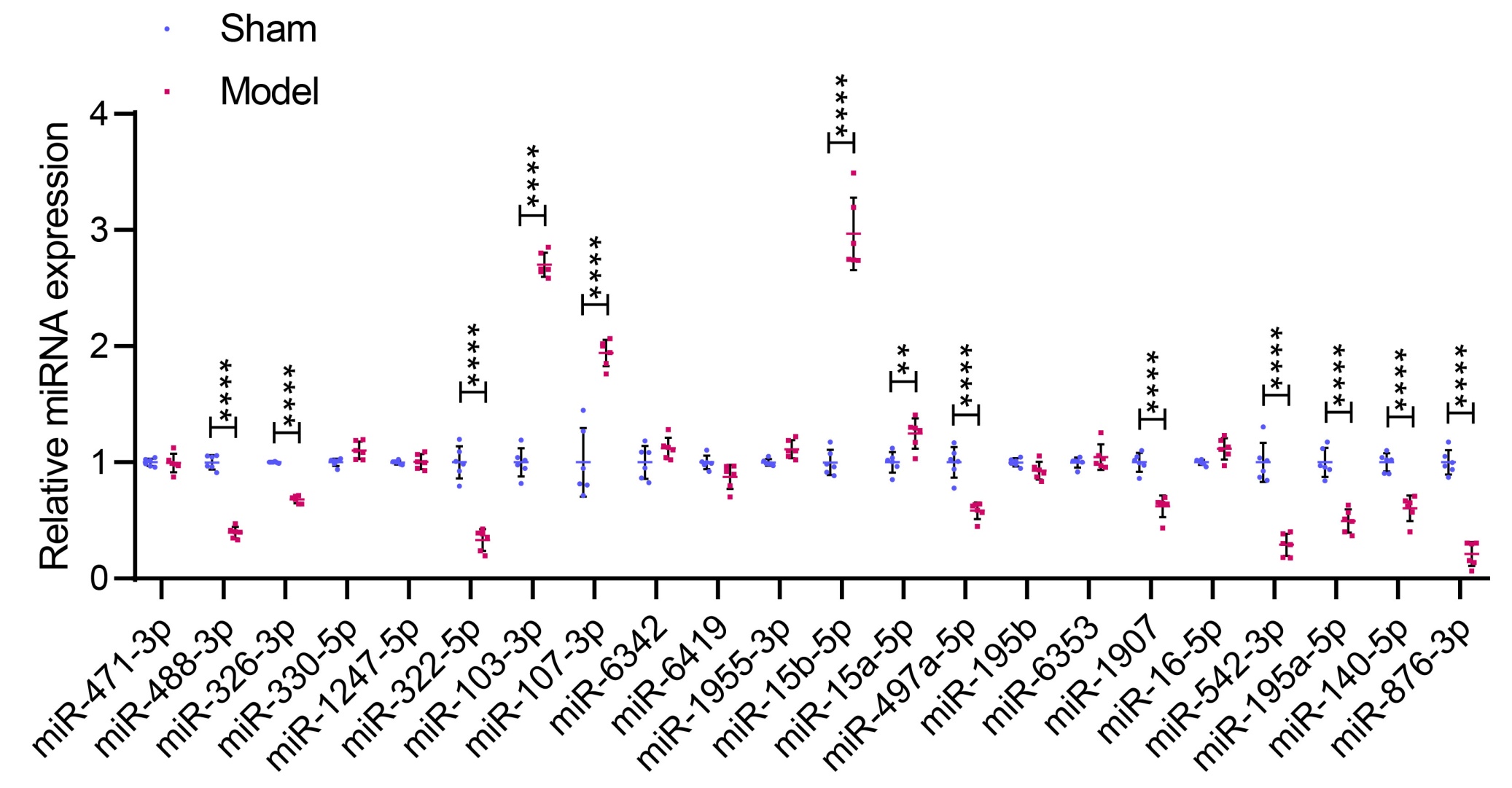
**

**Supplementary Fig. 2** qRT-PCR analysis of 22 miRNAs that can be sponged by circ_0000811 in the MVN of mice with CI-induced vertigo and sham-operated mice. * *p* < 0.05. The independent sample *t* test was applied for the comparison between two groups.

**
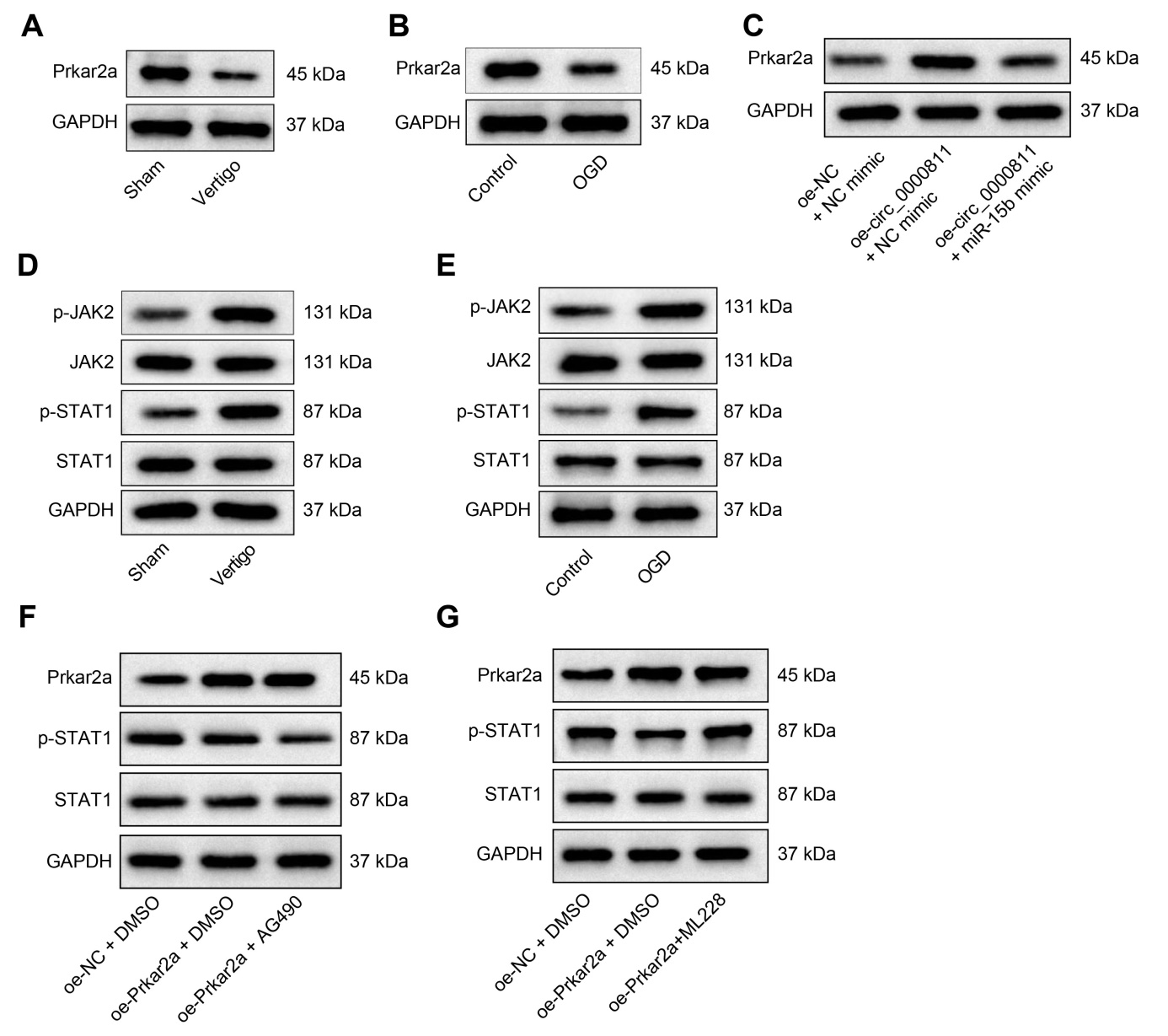
**

**Supplementary Fig. 3** Representative immunoblots for protein expression quantification in Figure 4E (**A**), 4F (**B**), 4I (**C**), 5A (**D**), 5B (**E**), 5C (**F**), and 5E (**G**).

**
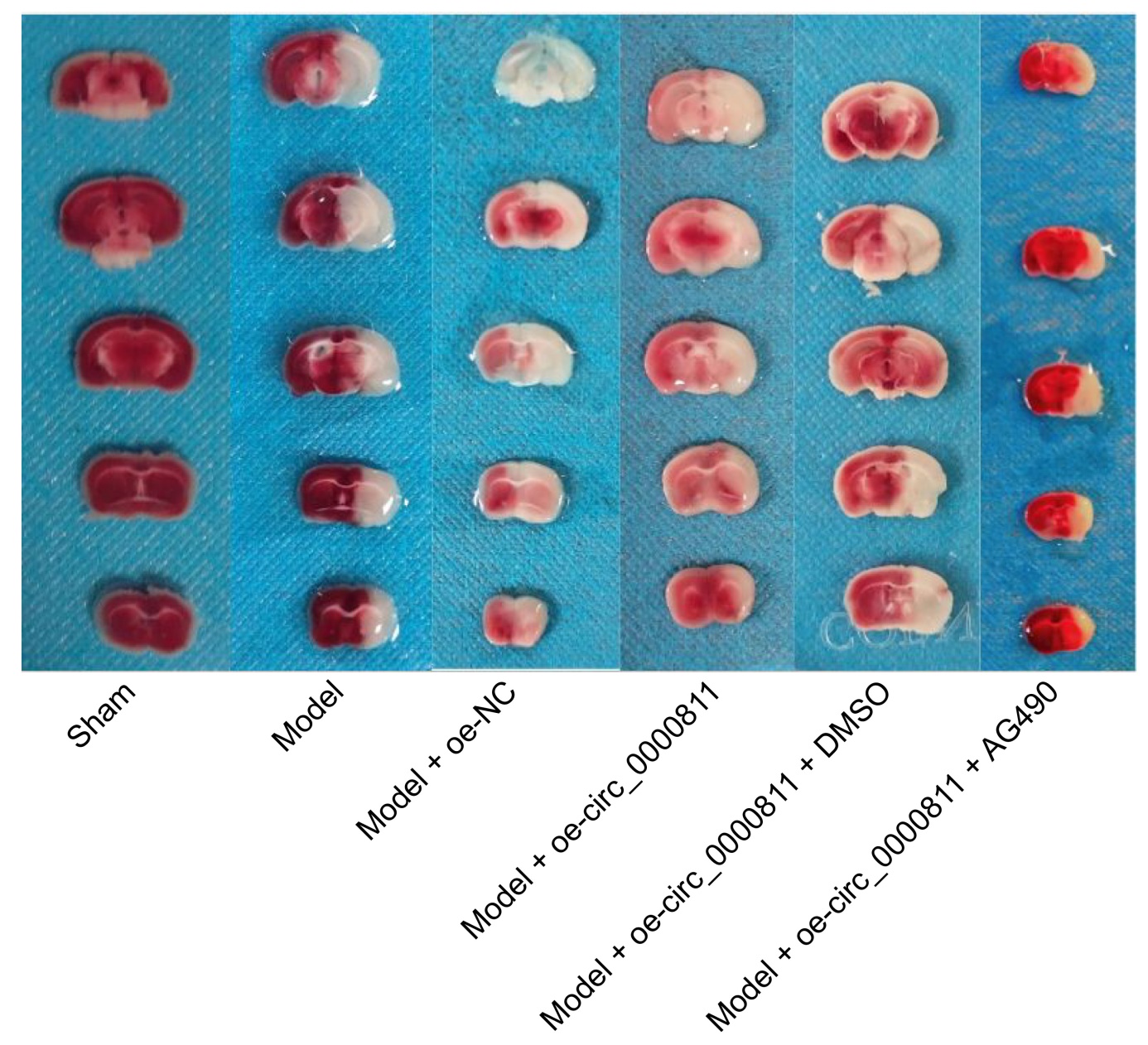
**

**Supplementary Fig. 4** TTC staining of the brain tissues of mice with CI-induced vertigo. TTC staining was conducted to detect the hypoxic-ischemic brain injury in mouse models of CI-induced vertigo in the presence of circ_0000811 overexpression alone or in combination with AG490.
