# Supplementary material for: Circ_0000811 acts as a miR-15b sponge and inhibits Prkar2a-mediated JAK2/STAT1 pathway to attenuate cerebral ischemic vertigo
Source: Cell Death Discov. 2022 May 4;8:247. doi: 10.1038/s41420-022-01016-2 (PMC9068921; doi:10.1038/s41420-022-01016-2)
Supplement: Supplementary file 2 — Supplementary Table 1 [file 41420_2022_1016_MOESM2_ESM.docx]

**Supplementary Table 1 Analysis of circ_0000811 based on circBase website**

>mm9_hub_77_memczak_circRNAs range=chr17:71110415-71136549 5'pad=0 3'pad=0 strand=+ repeatMasking=none

TGTCATCTTGCATTACAACATATAAGAAGACACCACCTCCAGTCCCACCC

AGAACTACCACGAAACCTTTCATTTCTATCACAGCCCAGAGTAGCACAGA

GTCTGCGCAGGATGCCTACATGGACGGGCAAGGCCAGCGCGGAGACATGA

TCAGCCAGTCTGGCCTCAGCAACTCCACCGAAAGTCTGGACAGTATGAAG

GCTCTCACGGCTGCCATCGAGGCTGCAAACGCCCAGATTCACGGCCCAGC

AAGTCAACATATGGGCAGCAATGCTGCTGCTGTCACCACCACCACCACCA

TAGCCACTGTCACCACCGAGGATAGGAAGAAAGACTTTAAGAAAAACCGA

TGTCTGTCTATTGGGATACAGGTAACTGCTTCCTATACCTTGAAATAGAA

CCCACTGAGAAGTGTGCCTCTTCGTGTGTGTGTGTGTGTGTGTGTGTGTG

TGTGTGTGTGTGTTTCCAGGAAGTCATTTTGCCACTGGGGCAGGTGGTGC

ATGCCTGTCATCTCAGCACACAAAAACCTGAGGCAACAAAAGTTGCAAAT

TGAGGCCAGTTGTAGTGGCAGAGAGAGACCTTTAACTCCCAGCATAGGCA

AAAAGCTCTGCGAGTCCAAAGCCAGCCTGGGTAACATAGCAAGGTTCTGT

TAAAACAAATAAAGAAAGGAGGGTTGAGAGTTCAAGGTCAACTTAGGACA

GTTTGGACTATATATGCCTTTCTTAAAATGAATCAAAATACCAAAAAAAA

AAAAAGAAAAAAAGAAAAAACAAAATTATATCTGCCATTATTTTGTATGT

ATATCAAAGTACCCTTATGTGTCTTGTGTACATTTTGATTCTCAAACAGA

GGTCAGGAGCTATGGCTGTGGCTAAGCGCAGTTGCTGCTTTTTGGGGGGG

GGGGGGCGGGGGGGGGGGAGAGGCGTTGAGACACGGTTTCTCTGTGTAGC

CCTGGCTGTCCTGGAACTCAGAAATCTGCCTGCCTCTGCCTCCCAAGTGC

TGGGATTAAAGGCGTGCACCACTACGCCCAGTGGATTTTTCATTTTTAAT

TTAGTGTGAGTTCGCAAACTGAGTAGGAGGAGCTTGTGGCAGAGAAGTGG

GAGGAGCTTGTGTGCTGTGCGTACAGGTCAGGAGAGTACTCTCAGGAAGC

TACTCTCCTTTACTGTGTTAGCACCAGGATTGAATTCAGCTTGATAGACT

TGGCGGGATGCCTTTTCTCAATGAGTCATTTTGCTCATCCAAGAATAGCA

TTTTTTTTGTTTTGTTTTGTTTTTGTTGTTGTTGTTTTTTGGTTTGTTTG

TTTGTTTCGAGACAGGGTTTCTCTGTGTAGCCCTGGCTGTCCTGGAACTC

ACTCTATAGACCAGGCTGGCCTCGAACTCAGAAATCCGCCTGCCTCTGCC

TCCCAAGTGCTGGGATTAAAGGCGTGCGCCACCACCGCTCAGCCAAGAAT

AGCATTTTTAAAGCTCAAAATTCCAGGCCTGTAAGATGGCTCAGCGGGAA

AAGGCCCTTGCTGTACAGCCACTCAGCTGAGTTTGAACCCAAAACCCAGT

GTAGGAGGAGTCACACAACGCCTGAAAGTTATCCTCTGCGACACAGGAGC

TCTGTGGCAGGCACCCTGCCCGGTGCCACAGATTTAGGCAGTCGGGATTC

CTGTGAGAGTTACTGTTTAGTGGACACAGGTCTCTCTTTGGGATTCGGGT

CAGTTCTACCAAGGCTGTTTGTGGTCGCTGAATGTGCTCAGTGCTTCTCA

ACCATGCATGTCACCATGGTTACCGTGGGAAAGTTTAGGTTACACACTTT

CGCCATGGTGATTACAGTATCTGACTTTCTATCTGACACGGTGTTAGCTC

CATGGGGTGCTAGCTTGCTTCATTTCCTCAAGATTTCATTTTTTTTTTTC

TTTTTCTTTTCTTTTCTTCTTTTTGGTTTTTCGAGACAGGGTTTCTCTGT

GTAGCCCTGACTGTCCTGGAACTCACTTTGTAGACCAGGTTGGCCTCGAA

CTCAGAAATCTGCCTGCCTCTGCCTCCCGAGTGCTGGGATTAAAGGTGTG

CGCCACCACGCCAAGTTACTTCATTCTTTTTTCAAACGTCTTTATTGTAT

GTCATTTTCAATAGCTAATGCATCTTCGTCATCTAAAACTCAAAACATGG

TTTAGGTTTTATTTACATATTCTCTGTGTGTGTATATGTGACTGTACCTG

GATGCATATCTGGGTACCATGTTCATGCAGGAGCCTGCCTTGGAACTGGA

GTTAAAGGCAGTTGTGAGCCACTATGTACTAGAGACTGTACTTTATACTC

AGCAAGAGCAGTGAATGCTTCTAACCTCGGAGCTATCTCTCTAGCCCCCA

AAACTTATAGATGTATAATGCTAATACTCCCTGACCAGTTATGATCAATA

TTCCATTCAATTGAGGGAAATCAATGCACTCAATATCTCTACACACTGAT

ATTTTGTCAATATTCTAGCCAATATATATTTAACTTAATCCTTATTTTTG

CCCTGGTGTTAGCATGTACCTTACAATATTCTCTACTCTGTGTGTGTGTG

TGTGTGTGCATATGGGCTTATTTATGCCATGGCTTGCTTGTACAAATTAG

AGGACAAACTATAGGAGTTGGTTCTCTCTTTCCAGCATGTGGGTTCTAGG

GATTGAACCCCCATCCATGATCAGGCTTAGGAGCAAGGGCTTTTATTGAT

AGAACCACCTCTACAGCCCTTTTTTTTTTTTTTTTTTTTTTTTTTTTGGT

TTTTCGAGACAGGGTTTCTCTGTATAGCCCTGGCTGTCCTGGAACTCACT

TTGTAGACCAGGCTGGCCTCGAACTCAGAAATCCACCTGCCTCTGCCTCC

CAAGTGCTGGGTTTAAAGGCGTGCGCCACCACGCCCGGCTACAGCCCTTT

TTTTTTTTTCTCTCTCCTTTTTTAGACAGGGTTGGTTTCTCTGTGTATCC

CCTGGCTGTCCTGGAACTTATTCTGTAGACCAGGCTAGCCTCGAACTCAC

AAAGATCCAACTGCCTCTGGCTCCTGAGGCTGGGATTACTCTGGTGCACC

ACCACCCTGGGATTCTGTGGTGCTAGGGATGCAGCCCAGGGCTACTTGAG

TGCTAGGCCAGCCACCTGACAGCTGAGGTATGTCTCCATTTCCCCTTTTG

CATCTTCACTGATAATTTAGAACCTCCAAGAGCACATTTGTTTGTGGCAT

TCTCCTTAATAGAATACACTCATAAGAAATTACATATAGTTACCCATCAA

TATGTTTGGTATATACAGACTCAATCAACCACAGCTCCAAAATTATTAAT

ATTAATTATTTTTTTTTTCAAGACAGGGTTGCTTTGTGCAGTCCCGGCTG

TCCTAGAACTCACTCTGTAGACCAGGCTGGCCTCGAACTCAGAAACCCAC

CTGCCTCTGCTTCGCAAGTACTGGGACTAAAAGCATGTGCCACCATGGCC

CGGCCCAAAATTATTTTTAAATGTAATTATTTTTAAATGTACCTCCTTGG

GGTAGGAGAGGTGGCTCAGTTAAGAGAACCTGTTCGTCTTCCAGAGGAAC

TGAGTTTGGTTCCCAGCACTCACAGCTGTCCTCTAACACAAGCTGCAGTG

GATGGTTATCTCCTGATTCCACAAGTAGCCCCGTGCATGTGGCATGTACA

CACAGAATCACATAAACAAATAGGAGCCAGGCAGTGGTGGTGCCTTTAAT

CCCAACACTTGAGAGGCAGAGGCAGGTGGATTTCTGAGTTTGAAGCCAGC

CTGGTCTACAGAGTGAGTTCTAGGACAGCCAGGGCTACAGAGAGAAACCC

TGTCTCGGCAAAAAAAAAAAAAAAAAAAAAAAGTATGAATCTTTAGAAAA

GTAAAGTGCATCTGACTGGGTGTGTTGGTATATGCCTTTAATCTCAGAAT

GCAGGAGGCAGTCTCTGAGTTCGAGGCCAGCCTAGTCTACAGAACAAGTT

CCAGGACATCCAAGGCTACACGGTGAAATCCTGTCTTAAAAACCCAAAAC

TAAAACCAAAAAAAAAGAGAAAAGTGTGTCTATATTTCACTTGTACAGTT

TTAGTTCACTCTTTTCTTAGCTGTACAGTATCAACTCTGCATATCGTATA

AGTACTGTTAATTGGAAATGCTCTAGAGGCGGTCATGTACAAGGGAGGAT

GGATGTAGTAACAGGTAGGCACAATGCCATCCATGTAAGAATCTGAGCTT

TCTTGAATGTGGAGATCTATGGTGATCCTAGAACCAGTTCCTGTGGAGAC

CGAGGAACCACTATGCTTATAAATCTGTGGAGGAATCACAGACATTGGTC

ATCTTTGGAAGTAAATAAAACTGTGTTTTATTAAAGCCAGCAGAGTCAGG

GGCTAGAGAGATGTCTCTGTGGTTAAGGGCACTTAACTTCTGCTGTGAGA

GAGGACCGAGGTTCAGCATCAGCATAGTGGCCCACAACTGCCTGGAACTC

CAGCTTCAGGGGATCTGTTGCCCTCTTCTGGCCTCGCCAGGTATTTGCAC

ACATGTGGTGTGCATATATATATACACACTCAGGTAAACACACATACACA

TTAAAAAATTTCCTAGGAAATATTAGATGAATCCGAGAACACACTAAGAA

TGTAGTAATCGCAGCCGGGCGAGGTGGCGCACGTCTTTAATCCCAGTACT

CCGGAGGCAGGCGGATTTCTGAGTTCGAGGCCAGCCTGGTCTACAAAGTG

AGTTCTAGGACAGCCAGGGCAATACAGAGAAATCCTGTCTCGAGAAAAAA

CAAAAACAAAAAATAATAAAAAGAATGTAGTAATCGCTGCTATCAGATGC

ACAAATGTGGATCAAAGAGGTTGTAAGTCACCGAACAGTCCCGTCATATC

CCAGAGGTGAAAGCCTGTCTGCTTCTCCCGTGAGACAATGGAAACAACTC

TGTTTCCTTGATGATTCCTTCAGGTGGACGATGCCGAAGAGCCGGAGAAA

ATGGCAGAGAGTAAGACGTCCAATAAGTTCCAGTCCGTGGGAGTGCAAGT

AGAAGAAGAGAAGTGGTAAGTCCCGAGGTGGAGGCTGGAGGCGGCAGCAC

TTCCTGTCTGGGCGGTAGGAGGGTGGCACAGTATCTTCCCGTGTAAATAC

TTCCCGTGTAAATACAGCGTGGCGGCTGCACTCATCTGTGGCTCTGCTCC

CGTCTGAGTGGCGAAGGGCCGCACAGTATTCATCCCGTGACAAGCCTGCA

AGCTCACTGGCCTTGCCACCTATGCTTGGGAGTGTTTAGAAACATCAGTC

GATGCTAGGTGTTCCCCCAGCCCCACCCCTACCATCCCCCACTCCCCCGC

CCCTTGCTGAAATCCTGGAGAGCCAAATATCTTTTGAGCCAACAGTAGCT

GAACTGTCCTCCCCTTCACACAAATACTGGATGCAAATGTGGAACTGCCA

AGAAACTTTATGAAACTTTGCAGCCATTTTTATCTTTAACTTCATTAGTA

GTACTAGTAGTAGTGTTTTGTTGCTGTCAAGACAAGAATAATGCACTAAG

TAGGCTGGCTGACCTAGAACTTAAAACCAAGCTGGCCTCGGACTGCCTCT

CCCTGCCCAGTGCTGGGGCTAAAGGTGTGTACTACCATGCCCAACTTGTG

CTATTATTTTTTTAAATTGTGCGTGTCTGCGTGTGGGTATGTGCTTGGGA

GCACAGGTACACTTGCGTGCATGCGTGCACACACATACAATAAGTAAATG

TTAAAAGGTTTTGAAATGGGGCTGGTGAGATGGCTCAGCAGGTAAGAGCA

CCGACTGTGCTTCCGAGAGTCATGAGTTCAAATCCCAGCAACCATATGGT

GGTTCACAACCATCTGTAACAAGATCTGACTCCCTTTTCTGGAATGTCTG

AAGATAGTGTACTTATAGATAATAAATAAATCTTTTTTTAAAAAAAAAGG

TTTTAAGTACACAAGGCCCATTTTTACACTGATGTTTAAAGTGAACTTAC

TGCAGAATCCTAGTACCCAGAAGCAGGAAATTTAGAATTCCATAATATCC

CAGAACCCCAGTGCTGTAACAGATATCTGGTGGCAATATTTTTCATTTTA

TGAATGAGATTCTATAATCCTATGGACTCCCGATGTCCTGATTATAGATT

CAACAAAATGAGGGAGGGCTGGGGAAGAGACAACACCTGGACTATGAGGA

CAGGTGTGTGCGCATCTCTCTGTCTCTCATACACACACACACAATTTAAA

AAAAAATCAATCTTTAACAACAGAGCTATATGCCTTCTTTCACCCCATGT

ATCTAACCCCTGCCTAGTGCTAGCCTAAGGGACCCTGGCCAGATCCCTGC

ATGTCTATGTGCTTGACTTCTTTAGCATAAAGGGGGTTTTCCAGGATTTC

CTGCATTTCAGGACCTGGGAAGTCACCACTGTACCCTTCTAATAAATACA

AATCTGAACAAGCCAAAAAAAAAGCATATTCCCACATCTACTAATGAAGT

GAAGTCATGTGGCAAGGCAGACCCTGCCCCCTGCTCCCCACCACACACAC

TCGAGACTAAAGAACAGAAGACAGAACCCAGATACAAACTATCAGGACCG

GGGAAGGGAAGACAAGCCCAAACTATCCCCAAATTTCTGGCACCTCAGAC

AAAACTGTCCAGTGGGAGCTTAGAGTGCCATACTTGATAAATGTTACTCC

AGGGACTCTCCTCGTCCTGCACAGTGAACAGCAAAGGAAAGGGAACCATT

AAAAAAAAAAAAAAAAAAGATTTATTTATTTCATGTATATGAGTACACTG

TTGCACTCTTCAGAAACACCAGAAGAGGGTGTCAGATCTCATTACAGATG

GTTGTGAGCCACCATGTGGTTGCTGGGAATTGAACGTAGGTCCTCTGGAA

GAGCAGTCAATGCCCTTAACTGCTAAGCCATCTCTCCAGCCCCCTGAATC

ATTTTAAAACTAGGCCCAAACTCAGCAGAAACAGTATCATGTGCATCCTG

CCATTTCATCCTACCTGGGGAAGAAATATTCTCTTCTCTTCTCAGTTTTC

AAGTGTGGGGGAAGGGATATGCCCAGCTCCCTACCTTACCCCACCCCGCT

TAGCCATCTCATCCCAAGTAAGAGAGAGAAATAACTTGAGAAATCCCCAT

AAAATTACAGTCCAGGGGACAGGGTCACCAAGAGGATAAGACAACCCCAG

GGATGTGGGAGCAAACCTCTGCATAATTACTGGCAAGGAGGATGCTCTGT

CACTTCTGCCAGTATATAAGTGCCTGCCATGTAGAGAAACTGTAAGAATT

AAAGTTGTAAGAAAGCAGAGCTGGCGAGAAGGCTCTGTGCCTAAAGGCAG

TCACACGAGGACATGATGGAAAGAAACAGCTGACTCCAAACGTTGTCCTC

TGACCTCCACACATGGACTACAGGATACACACACGTGAGTGTGCAGACAC

TAAGTAAGTACAATGAAAAAACATTTAAAGCCATATTAGGTGGGTGGTGC

CACATACCTTTAATCCCAGCATTAAAGGGCAGAGGCAGGAGGATCTCTAT

GAGCCTGAGGTTAGCCTGGTCTACAAAACTAGTTCCAGGATAGCCAGGGC

TACACAGAGAAACCCTGTCTGTTATGAAACACAATATGGAGAGGGGAAGG

GGGTACCTTTGGAGGGCCCATGCCAAGGAGTGCCCCTGTAATGACGCCAT

GCAACAGACCTGGTGTGTATATAAGAGGTTCACTGGGGGACGGGAGAGGA

AAAAGGGCCTGGGAAGGGATAGAGACAGATAAGTGGACATGTAGAGACAG

ATGGGAGCAGAGTCATGAAGGCAGGGGTGGGGAGGCAGAGAGAAGTGGAG

GTACCTGCAGAGAACACATAGATGGAACGCTGGGGACAAACAAAGCTGTG

GAATGGCTGGCGGCTCCTTTTCTCTGGAGCACTCACCAGTCCCTGGCACT

GTCAGGTCATGAAGTAAGCAGTCACTAGGTCCCTGAGGGCAGGCCAGGAG

AATGCATGGATACAAACACCATCTTGAAAAACAAACAGGGGGCTGGTGAG

ATGGCTCAGTGGGTAAGAGCACCCGACTGCTCTTCCAAAGGTCCGGAGTT

CAAATCCCAACAACCACATGGTGGCTCACAACCATCCATAATGAGATCTG

ACGCCCTCTTCTGGTGTGTCTGAAGATAGCTACAGTGTACTTACATATAA

TAAATAAATCTTTAAAAAAAGAAATAAGAAAGAAATATTATAACCTGTTC

TTCGAAAACAAGAAAAAGGGCTGGAGAGATAGATGGCTCAGAGATCTTCC

AGAGGACGCAGGTTCAATTGCCAGCACCCACATGGCAGCTTACAGCTGCC

TGTGACCAGTTAGTTCCCGAGGGACTAATACTAATCTGATGCTCTTCTCT

GGCCTTCATGAGTTCCAGGGACACATGTGCTACACAGACATCCATGTAGG

CAGAACACCCAAACACAGAAAATAATAATTTACAAAGAGATCATCTCAAG

TCTTACACTTTTATGCAAAAGCGTCCCCACAGTCAAATAACCTGCCGACA

CTTGTCCCTCTATAAGAATGCTGGGTATGGAAGGGCCATGGGATGCCCTG

ATAGAAGGAGGCATTTGCCACACAAACCTGATTACCTGAGCTCAGTGCCT

GAAACCAACCTAAAAGTAGAAAGTTGGACTCATCTGATCAACAAGCCTAC

ACACATACATACATATGCACACATATATACCATTAGTAATTTTTTTTTCT

CTGAGGGGACACATTGCTACAGCATGATTTTCTAGACTCCTCTCCCCCAC

TTACTTAATTAGTGAGGAAGACACCATGAAATGGATCTGTAACTCAAGTG

AGTGGGGTGGGTGGGACACCGGACAGAGTAACCGCTGTGTATTTGAACTA

TTAAATTTTGGGGCTTTTTAGTTGCCACTGTTAAATGGGGGTGAAGATAA

AGGCTGAAGGAGTCGATTTGTATTGTCTTAGGTCAGCTAACCTTGGAGCA

GACATGGTATTTGAAATTCTCTCTACTCGGAGCTGTATTGTAAATGGTAC

ATAAACTTTTGTGCATTTTCCTGTATGGAATGCTAGTGTGCACAATAAAC

CCACACCTTTGGGTTTTTTTGTTTTGTTTTTAATACTAAGAAGCAATAAT

AATTAAAAAAAAAAGACAATGTGAACAAAAAAGGAATTTTTGGAATAGGA

AAAATACGAATGCCAGGTATTTGAAAAACTATATGACCATGGTCAACACT

TTAATATACTTGAGAAATGTTTTACATGTTAATGAGGTGAGTCTGTTTTT

TGTTTTTTGTCTTTTTTGAATAATGAATAGAAATGCAGTCCCTCCCTGGC

TGTGAGGCTAGGGACCCCAATGTAGTAGATTTATTATTTGTTGTAATAGA

TGTATTATAGATGTATTATTTGCGCTGCACCTTTGAGCCTGCTTCTAGCA

CAGCCTATGCCAAGGAAACAGTACGAGGTGAGCATCTCAGCCATTGACCA

AACTAGCTAGAGTAACACTTCCGGGTGAACCCCCGCCCTGTGGCTGGGAG

GAGAGTGAATAGAGGATGGCCTTTGAGGATCTTAAAAAAAAATGGTTTTT

AGTTGTGTATGTGCATCTGTATGGGGATTTGCACATGTAAGTGCCCACAG

AGACCAGAGCCATTGTATCTCCTAGAGCTGGGGTTACAGGACATTGTGAA

CTATCTAACTTGGGTACTGGGAACTGGACTCAGGTTGAGACTGAATTCAG

CTTATAATGGGAGAACATTATAAGCGTTCAACTGCTGAGCCTTCATAATA

CTTTTTTTGAAAAGTATTTAAAATGCTGGCTTGGCTGGTGTACTGGCTAC

TTTTGTGTCAACTTGACACAGCTGGAGTTACCACAGAGAAAGGAGCTTCA

GTTGAGGAAATGCCTCCATGAGATCCAACTGTAAGGCATTTTCTCAATTA

GTGATCAAGGGGGAAAGGCCCCTTGTGGGTGGTGCCATCTCTGGGCCGGT

AGTCTTGGTTCTATAAGAGAGCAGGCTGAGCAAGCCAGTAAAGAACATCT

CTCCATGGCCTCTGAACCAGCTCCTGCTCCCTGACCTGCTTGAGTTCCAG

TCCTGACTTCCTTTGGTGATGAACAGCAACATGGAAGTGTAAGCCAAATA

AACCCTTTCCTCCCCAACTTGTTTCTTGGTCACAAAGTTTGTGCAGGAAT

AGAAACCCTGACTAAGACAGCTGGTCAGTGATGCACGCCTTCAATCCCAT

CACTTGGGAGGTGGAGACAGGTGGATCTCTGGGTTCAAGGCCAGCCTGGT

CTACAGAGTGAGTTCCAGGGCTACACAGAGAGACCCTTCTCAAAATAGAT

AGATAGATAGATAGATAGATAGATAGATAGATAGATAGATGATAGACAGA

CAAACAAATAAATGCTGGGCATGATAATTCCAGCACTCTAGAAATGAAAT

GAGTGGATCTCTGTGAATTGAGGCCAGCCAAGGCTATGTACTGAGACCCT

GTCTCAAAAACCCAAACAACTAAGTAAATAAACAAATTAATCTCCTAAGA

AGAGCTGACAGTAATTTTAGGGCTTAACCTGCTAGAATGTCCCTCCACAG

GTCACTTTGTAGTTCACCTTTCTGTGAATAAATAAGCATATCTATGAATG

CCTGTTTCTGAAAGAAAACACATTGTCAGACATAGAGGGCTTGCTTTCAT

CTTCTGTCTCTTATAATTCTTTTTTATGATCCTGCCGGTGGCGGTTCACT

TAGATACCATATCACAAAGCAGCACTGGGCAGTTGAAATTCCTTCCTGAT

GACTGGGGGAACCCCCCCCCCCCCAATTATCTAGTGTTAATTAGGAGCCA

GCTGTTTTCACACTGAATACTAGATAACAATCCGGGGCTTCCTTACCCAA

ACAGACTGGACTAAGAAACCCCAACCTCACAGAGAGCGAGAATAGAAAGA

AAACAGAGCCCGGAGAAAGAGATGAATACATAAGAAGCCAGGAGACCCCC

AGGGTTTAGAATGGTCCCAGTTTAGGTAAACAACAAGCCTGAAAAACCTA

CCTGACTGGTTATTTAACTGAGCAGGAGACTTCACAGAGTTCTGAATTAA

AATAAATCAGTTCCTAAGTTATTTGTAAAGAAGCACAGAAGCAAGATGTG

TTGGGCAGCCCTCTGCTTCAAGAAGAGGCATAGACGCGGAACAGCACAGA

GTTTGTGGGCAGCTAGGACATCCTCGGAACGTCATGGGTAGCAGATATAC

CGTGTCCCCTTTCCTGGGCAGGCTCACAGTAGGGCGCATGCACAAAGGAA

TGCTTTAAAGGCCCAGAGTCGTTTTGTAAGGAGGCGGTTCTCTTCAGCTT

AACTCACTACCTTTGTCAAAAACATCCCCCAGTTCCTACTCTGTTCGCAG

GCCAGCTAGTAACACTGATCCACAGGGTACATATTTGCCCACCTGAAGGG

TGGATTCTTAGCAAAGCAGCTCACACCCACAGTGCCTCCTCTGCATCCCT

GGGTGCAAGCCCTCCTTCATCTGCTCCATCCTCTCTATGGCTTTTGACTG

CTCCCAGGAATCCCTATGAAGTCTTCTAGGCTTTCAAGCTGATAATTAAC

AACTCTAGGAAAGTAATCCTTTCTGTTGACTAAGGTGAGAAAGCCTGAAG

CTCCCAGGGGGCTCCCCTTTCCCTAAGTGACATTGTATTAGCTCAGTGAC

TCTAAACTGCCCTTTGTTACATGAGCCCACTGGTAATTCTGACCCCAGCA

AGAGACACAAAAAGGGAGGTGATTACTGTCTACATGATAAAAGGACTATG

CAGATTTACTCAGAGGCAGAGATATAGTGACAAGGTAACAATGTATAACC

CGTAAGATTCAGAGTCAAGGGGAAAACAAAAGCCACCTGTGTCCGTGTGT

GCCTGTATCTCTTCTCTTTACAATGTTGTTTTTTAAATAAGGATTGTGTA

CTACTGAATAATTCAATTAATGCTAAGAAAAGTAAGGGCTGGAGAGATGG

CTCAATGGGAAAGAGTACTGGCTGCTCTGGCATTTGACCTGGGTTTGATT

CCCAGCACCCACATCACCATCACCCACATCACGATTCACAACTGCTGTAA

TTACAGTCCCAGGGGATCTGACACCCTCTTCTGGTCTCTGAAGACACCAG

GAACATATGTAGTACACACACACATACATGCAGACAAGTCAGCCATACAC

ATAAAATAATAATTTTTTTAAAATCACTACATAAGTGTTCTCTTTGTTAT

TAAATACTCATGCCAGATAGCTTAGCACTGGCTGCTGAGCCTGAAGACCT

GAGTTTGATCCTTGGGACCTTAAATTGTCCTCTGACACACAGCCTCCCCC

CCCTCAACACACACACATACAAAAAGAAAAATAAATGTTTTTAAAAATTT

TTAAAACAACAACTTGCTACCTTTAATGATTGCAGCACTCCAGAGACATA

GGCAAGAGGACCTCTGTGAGTTTGAGGGAGGCCATCCTGGTCTATATAGT

TCGTCCAAAGATAGCCAGGAGTTAACAGACTGTGACTCAAAACAGGGAAA

AGAAAACATTTTCTGTGTTTAGTTGAGGAAGCATTGTTATTGACTTCTGT

TTTTTCCTTCTACTGTGTCATCTTGGTGGATGTACAGTGGTATCTATCTC

ATTAGCGCTTTGGTTTGAATTTCTCTAATATTGAGTTATGTTTCCTGTGT

CTCCTGGGCAATTGTATAGAGAGTGTTTATTCACGTTATTTGCCCTTTTT

ATAGAAATTGGACTTTTTGTTCTTTGGTTATTCAATATTTTAAGAATTCT

TTGTATATTCTGTACACCAGATCTTTATCAGATTTAAATATTATTTCCCT

ACATTCTATGAGCTGGCTTCCCACTCTCTGTATAGGGTGTTTTGAGATAA

CAGTTTTAAATTTTGAAAAAGTCCAACTCATCCCTTTGTTACTTATATTT

TTAGTATCCCACTTTAAAAGTTATTACCAAGGGGTTAGAGAAATGGCTTG

GAGGTTAAGAACATTGGCTCAGTGGTTAAGAGCACCGACTGCTCTTCTGA

AGGTCCAGAGTTCAAATCCCAGCAACCACATGGGGGCTCACAACCATCCG

TAATGAGATCTGATGCCCTCTTCTGGTGCGTCTGAAAACAGCTACAGTGT

ACTTAGATATAATAATAAATAAATCTTAAAAAAAAAACAAAAAAAAAAAA

AAAAACACTGGCTGCTCTTCCAGAGAATCCAGGTTCAATTCCTAGCACCC

ACATAGCAGCTTATAGCCATTTATAATGGCAGTCCCAGGGGTCTAACACC

CTTTTCTGACTTCCCTTGGCTTCAGATATAAATATGGTGCACAGACATAC

ATACAGACAGAGCACTCATACACATAAAAATAAATTAATTAGGCCGGGTG

TGGTGGCGCACGCCTTTAATCCCAGCATTTGGGAGGCAGAGGCAGGCGGA

TTTCTGAGTTCGAGGCCAGCCTGGTCTACAAAGTGAGTTCCAGGACAGCC

AGGGCTATACAGAGAAACCCTGTCTCAAAAAAAAAAAAAATTAATTAATT

AATTAATTAGGCCGGGCATGGTGGCACACGCCTTTAATCCCAGCACTTGG

GATGGAAGAGGCAGGAGGATTTCTGAGTTCGAGGCCAGTCTGGTCTACAG

AGTGAGTTCCAGGACAGGCAGGGCTGCAGAGAAACCCTGTCTCAAAAAAG

CCAAATAAATAAATAAATAAATAAAATTACTTTTTGCTTTTTTTGAGACA

GGTATTTTGGTGTAGCCTTGGCTATCCTGGAACTCATTTTGTAGACCAGG

CTGGCCGCAAACTCGCAGAGATCCACCTCCCTCTGCCTCCAAATGCTGAG

ATTAAAGTCATGTGCCATCATTGCCTGGCTAATTAATTATTTAAAAAAGA

AATCACTACCAAACCCAAGGTCAATATTTGTCTGTGTATATTCTTCCAAA

ATTATCACAGCCTTAGGGTTAGATCTAAATTTTACCTTTGATTCATTTTG

AACTATTTTTTTATGTGAAATAAGGTAAAAAAAGATACAAAATTATTCTT

CTCATGCGGCTGTTCAGTTAGCTGACATTCACTGACTTATTAATTTATTA

ATTTATTTACTTACTGGAGGTGAGCAGAGAGTGACGTCAGTGGTGCAGAG

CACTTGCTGTTCTCGCAGAGGGCTCCAGCACCCAGATGGCAGCGTTACCA

GTGACTACAGATCCAGGAGATCGGACATCCTCTTCCAGTATCTGAGGGCA

CTAGGCACAAATGTGGTGCATATGCATGCAGGCCAGCACACACATATATA

CAGAGAATAAAAGTAAATAAATGTTTTAAAGTAAAATAAATAAATAAATT

TGGTTGGTTGGTTGGGGGGTGGGGGTGGGGGGACAGGGTCTCCCTCCGTA

TGTAGTCTTGAAACTTATTCTGTAGACCAGGGTAGCCTTGAATTCACAGA

CCCACCTGGTCCTGTCTCCTAATTGAATATACCACCACACCGTTTGATTT

TTCTGTTATGTTAGCTTGACCTTGTATTTCCGTTTTATCATCCTGTGTCT

TTCCCACATAGTATTGGATTTGACTGACATTTTTCTATATTTATTTTCAA

TCTACATTCATGATGGATAGTGTTCTGAGGTTTTTGGGGGGGTTGTTTGT

TTGTTTGCTTGTTTGTTTTTGGAAAGGTCTTTGTGTCTTTGGTATCTGAA

TAATGAATGACATCACTGAGTTAGGAAATAGTTTTTCTGTTTTGGTTTTC

TGTGTAGGAGTCTGTATAAAATGTACATGTTTCATTATGTTTTCTTGAAG

TCAACTGTTGTATCAAGCATTTAATCTGAGTGGGTTTTCTTTTTTACCCT

AGATAAAAGACATAAAACTTTTATATTTATAATAACCCTTTAAAGCAGTA

GAGCTGGGCAGAGATCAACCCTCTAAGCTATTATATCTACTTCCCACAAC

CCCGAACTATGACTTGCCATGTTCCACTTGGGCTGCTCTTAACTTCTACT

GGACAGCCCTCATGGTCATGTTTTCATGACATGCTTTCAAGACCCCATGT

CATCTTCTCCTCTCTCCACCTTCTTTGCTCGTCTCTCCTCATGGTCCTCC

TCAGACCCCAAGCCCTGGAACTGAAACCTCTCTTCTGCCCAGCTCTAGGC

TGCCCTTCCTTATTCAGCCCGTAGTTTGAAATCAAGGAGCAAGGCTACAT

AGCATCACTTGGTGTAGGTGAGGATTTCCACTTCCCTGGGAGCAACCAGA

CTTTGGGGCCAGGATTTAGCACTAGAATACATAGCAACAGACCAAACCTC

AACAGTCAAGATGATGTAATTTACATATTTATTTTATTACTGTTATTTTG

AGATAGAGTCTCATTATGTAACCCTGGCTGCCCAGCTCTATAAACCATGC

TAATCTTGAATATAGACCATGCTAATCTTGAACTTAAAGCATTCCTTTTG

CTCAATTACTTGAATATTGGGATTTCAGATGGACACTACGATGCTTGTTT

TCTCTCTCTCTCTCTTTCTCTCTCTCTCTCTCTCTCTCTCTCTCTCTCTC

TCTCTCTGTGTGTGTGTATCCTCTGGCACATGAGTAAAACCTGTAGAATT

TACAAATCAGGTTACATGCTTCCAACGTATAATGTCCTTAGGAAAGTACA

GAAAAGCGGGATGAAATCACAGCAGAACCCAAGAGGACAGACAGAACTAA

ATCCTAGAGTTCCAGGTCTGAAGCATGCTGATGTGAGGAAAGCCCCAGAA

GGCCTGAGCAGCCCCACCTCTGTGGCCTGTTCATTCACAGGCCACATGGC

CTCTCTCTGTGACCATGGCTACATTGGGCTTAGAGTACCTGGGTAACTAA

CTGTACATGGTAAAAAACTCCTTAAGAAATTATCGCCCTGCTGGGCATGG

TGGTGCACCCCTTTAATCCCAGCACTTGGGAGACAGAGGCAGGCGGATTT

CTGAGTTCGAGGCCAGCCTGGTCTACAGAGTGAGTTCTAGGACAGCCAGG

GCTACACAGAGAAACCCTGTCTCGAAAAAAGAAAAAAGAAAAAGAAAAGA

AAAGAAATCATGGCCCTGTGCAAACAGGGTATAATAGAGGCCCCCTCTTC

GCAAGGGAAAAACTTCACAAATGACTGCTGTCTTAGTTAGGGTTTTACTG

CTATGAATAGACACATGACCAAGGCAGCTAACTCTTATAAGGACAACATT

TAATTGGGGCTGGCTTACTGCTTCAGAGGATCAAGTCCATGATCATTAAG

GTTCTATATCTTTATCTGAAGGCTGCTAGTAGAATACTGGCTTCCAGGCA

GCTGGGATGAGGATCTTAAAGTCACACCAAACAGTGACACATACTCCAAT

AGGGCCACACCTTCTAATAGTACTACTCCCTAGGCCAAGCATCTACAAAC

CATCACAACTACTTTTCTTTCTACTCTTTTTAAAAGAGATTTATTTATTT

ATTTATTTATTTATTTATTTTACATATATGGGTACACTGTAGCTGAACAG

ATGGTTGTGAGCCTTCATGTGGTTGTTGAGAATCAAATTTTTAGGACCTC

TGCTTGCTCTCGTCAACCCCGCTATCTCCAGTTGGCGCCACTTGCTCTAT

CCCTGCTCTGCCCCAAAGATTTATTTATTATTATAAATGAGTACACTGTA

GCTGCTGACTTTAGATATACCAGAAGAGGGCATCAGACTCATTACTGGTA

GTTGTGAGCCACCATGTGGTTGCTGGGATTTGAACTCAGGACTTTCAGAA

GAGCAGTCAGTGCTCTTACCCACTGAGCCATCTTGCTAGCTCTTTTTTTC

TACTCTTGAATCTGCCATGAATGAGGTCTAGTAAGTTCCTGAAATGTCCC

CCAAAACATCTTTCTTGCTGTCCCAGTGCAAAGCCATGTGGCATCTCTTT

AATGACCCTAGACTCTTCAGGAACCATGGACTTTTAGTTTTTGTTGAGAC

AGGATCTTACTCTCTAGTCCTAAACTTGTGACTAATGAGAGACCTTGAAC

TTGTGAGAGTCATCCTGCCTCCTAAGTACTGGGATCACAGCATGAGCCTC

TGCAGCTACCCCAAAGGGTTAGAGAAAGACAAGATGTAGGCAAGTTCTTT

GCCAGAGTATAATATGAATCGCTTCTACTCTGTTTCTCAATGGAATCTTC

ATTCCCATCTGAAACATCACAACATAATCTTAACTTTACATTTCTGTCAA

CACTCCGGTCTTCTGAATTTTTACCAGAATTGCCAGTTTGACTCTACTCC

TGGTAATCTCTAGGCTGCAAATCCACACTCTTTCAAATTCCTCCTTCACA

CCAGTTAGTTTCAAAGGTTTAAGAACTGGGGCTGACGAGATGGCTCAGCG

GTTAAGAGCACTGACTGCTCTTCTGAAGGTCCTGAGTTCAAATCCCAGCA

ACCACATGGTGGCTCACAACCCCATAATGAGATCTGATGCCCTCTTCTGG

TGTGTCTGAAGACAGCTACAGTGTACTTATATATAACAATAAATAAATCT

TTAAAAAAAAAAAGGTTTAAGAACTACATGGTAAAAAAACACAAAAAACA

AAAAACTACATGGTTAGATTTTAGTCATAGCAGTAACCCCACGTCTGAAA

CCAATTTTCTTTCTTTTTTTTTAAAGATTTGTTTATTATATGTAAGTACA

CTGTAGCTGTCTTTAGACACACCAGAAGAGGAAGTCATATCTTGTTATGG

ATGGTTGTGAGCCATCATGTGGTTGCTGGGATTTGAACTCAGGACCTTCG

GAAGAGCAGTCAGTGCTCTTAACCACTGAGCCATCTCTCTAGCCCCCCCC

TTTTTTAAAGATTTGTTTATTTATTATATGTAAGTACATGAAACCAATTT

TCTGTATGACTTAAGTCTGCCTGCTTATTTATGTTGCTTTGATCAAAATA

TCTCAGAGATTGGCTTCAAGTAGCAAAGATTTGTTTTATGGTTTCAGGAG

AATCAGTTCATGGCTGCTTGGTCCCATGTGCTTGGGCAGAGCATCACTGG

ACTGGAAACACGTGATACAGTTCTTCACCTGAGGCCGACAAGAATCAGAG

GAATAAGGAATAGCAATTACCTTCAGTAGCCCCAGTGACCAAGCTCCCCA

GCCACGGCCCTGCTTCCCAGAGTTTTTCTCACTGCACAAGTACTTCCTCC

AGTACTTGTGCTAGTCAGACCATGAGCCTTGTTTCACAGTGAATGCTGTG

TGAATTCAGACCTGTGAGCACTCAACGGAAAAGAAAGTCAGAATCTTTTC

CTATATGTTTGTGGAAAGAGAATTGACACAGAGACACTTTGACTTACAAA

ACAAACAAACAAACAAAAAACTCCCCTGCTCTGTTTCACTTGCCACTTAG

AGAGCCCCTGCCCCTTCTCCTACTGCAGCCCTCCCATTGTGCTTGGTACT

TAGACCGTAAGAGTTTGCTGACTATAAACAAACATACAAAAAGATATCTT

GGCATTAAAAACTTACAGGAATCAGTTGGTTCTCTGCACTGTATGGTTCC

CAGGGATCCAACTTAGGTCATCAAGTGAGCCACCTCACCAGCCCCAAGTC

CCTATTTTTCAGAATAAAAGAAAGAGCTCACAGCAGGCTCATGACTGGGC

CGAACAAGCTCTAGAGAAGCCACACCACCGTTCTCTTTGACCACAATGAC

AGCCACTATAAGAGATTCCTCCTTTGATCACAAGTGACTTCATATTTACA

CAGGTGTAAATATTTGAGCGCTTAGTGATCTCAGCTAAACGAGATACTAA

AACAAAAAGTAAAAAGCAAGAGAGGGGCCTGAGAAACAGAGGCCCGAGAA

ACAGCCACTTTAAGTCTAAGGCTACAATCCCTGCTTTGCCCTCCTAGAAA

TTCTGGGTTCAACACACTCCATACCATCTCCTTAAACAACCAAACAACCA

GCACCCACCCTTCTCGGTTCTCTTTCCTTTGCTTAACAATGATGTAACCT

AATCAGATCGTTCCTAGCACCTGAGTTTTAAAGGCGCGCCCTTCACAGGT

TTCAAATTTCAAAAGCCTGAAGTCCAGCCTAAATCCAAGCTGACCTCAAA

TGTATGGCCCTTAATTCTACAGAGAGCCCCCCCCGCCCCCCGCCCACCCC

TTCCTTTTCCTTTTCCCCTCCTCTCTGCCTCTCTTCCTTTCTTCTCTCCC

TTCTATTTTCTCTCTTTCCATTTCTTCCTCTTTGCATGTATATTTTGTTT

TTTAAAGATCATTTAGTTCTCAGAAAGCAAAGAGACTATGACTTTCACTT

AAGCAAAGCTGTGCTAAAATATGTCAGTGAGACTGAAGTACTAGCTCAGC

AGTTAAGACCATGAGCTCCTGTAGAGGGCCTAGGTTGGTCTCTTGCACCC

ACTCTTCACTTGGAAGCTCTGACTGGAAAGAGTACATTTTTCAGGGAGGC

TTGACCTCAGTCTCCTGCCCCTTTCTTTTGTACTTTACTTTGTATAATGA

GTTATGTTGGGTCTTTATCAGAAATGCTGCCAGCTCACAAATCACCAAGA

CTGCTGTGGCAGAGGGGAATACATTTTCAGATCCCACAAGAACCTTGACA

GGAGCTTGGTGCAAAAGCCCCTAAGAAGAAAGAACGGCCTAAGAGTCACA

TTCTCAACTTAGTCGCCAGACTTAGTTCTGAAAGACAAGTGCTATGGAAG

CAAAGGACTAAAGAGGATGACCAGAAACTGGGGGGGGGGGGGGGGGCGGG

CGGACGACCTCTGCCCTGGTATGACTGAATTTGCCTCCCTAAAATGTCCA

CTTGGCTTGAACGTCACTGGACCGCCAGGCAGGTCCAGACAGCAAACAAA

TGTCAGCATAGAAAGGTAGCGCTGTAGGTGAGCACTTACCTAGTTCCTGC

TTAGGCCTGGATTCAAACTCTCCAGCCTTCACACACACACCACACCACAC

CACACCACACCACACTGAAGATGGCGATGCTTAGCTGTTGCTCCTTTGTG

TCGCCCCCCACCCCCTTTCTGCGCAAGAACCTGTAAGAGCATTTGAAAAC

TGTTACACATAGAAACATTGTGGCAACACTGTAGAAAGTCTTTTCATCAG

AAACACCCACTTGTTTCACAGGCACAAGGGCCCCATCCCTGCCTGCTAGT

CAGCAGCTTGTCACTCAGTCTTCAGAACACACAGGGCATTGTCCTCTGCA

CTGGTTAATCCAGTCTCTCTTCGCTGGCTTTCTGCATCCTAGGCCTTGTT

CAGAAGTGTAATCATTACACACTGGGCCTGCAGGGCGTGCAGGGAGAGCA

TCAAGACTGGGAAGTGGGGGGGGGGGGGTTTGCAAGCTATGAGATCAGGC

TCAGCTTTCAGGATTACCCCCTTCACAGAGTTTAATCTTGGTGACCCCTT

TCCTGTGTCCCTATCAGCAATTTAAAACAATCACAACTTGATCACGCCCT

CTGCCCACAAGGGACTCTGCACTTGAAAGAGACAGGGCAGTTTTGTGGTG

CAGAACAGCCTTTGAAAGTTCTGAGTGTAAGTAAGAAAGCCTTTGCTTAT

GGGACTGAAGGGGTAGGAAGTACAGCTTGTGCGTGTCGTCGTCGTCGTCC

CCCCCCTGCAGTTCAAAGGCGCCGCCTTTAATCCCCAACAGTTTTCATTT

TGCTTAACTTTTATTTCTTTATGGGTGGCTAAACTGCAAACGTCTATTAA

TCTAGTGCCAAGTAATTTTTCTTAAGAAGGTGGATCCATTACCGGATCCA

CAGCCGGGAGGGAGATGGCTCTCAAGAATGCTGGTAGGACAGTTTTCTGT

GGCTGAAACAGAGAGGAAAGTGGGCCACTCTTCTCTCTATGGAGCAGATT

CCTTCCCTGGCGAGTGTGCTCTGCTGGAACACAAGCGGACTAGTATATTT

GGAAATTCAATCCCCTGGCAATCAGAGATCTTTGGGGAGAGAAGACTAAA

TATTTCTGCAGTTGTGATTTTTTTGTGTGTTAGTAGAACAGCCAAAATGT

CAGCCGTGGTGATGTGAACACTGTCCAAGTCACACACGGGCAGCTGAAGA

GAAAGCTTAACGTCAGGAAGGGGCATTTAGCATTTAGGGACAAAGTTGAA

GGAACAGAGAAACAGCCAAGAAGTGAGTGGGACATTTTACTAGAATTTTC

CTGCCTCTCTTGTTAAACCAAAGAGACTTCTGAACATGACTTTCAAACTT

GCATACCACACACATGACTTTCAAACTTGCACAACTTACCCCACTCCCCC

CCCCCCACACACACACACACTGGCTCATTGGAGAATTAACAACTCACATA

AGTAACAAGAGCTGATCATATCAACAGCTTTTGATGTGTGTGTGTGTGTG

TGTGTTTGTGTGTTGAGAAAGTTTCTCACCATGTAGTCCTGACTGTTCTG

GAGCTCTCTATGAAGAGCAGGCTGGCCTTGAACTCACAGAGATCCACCTG

CCTCTGTCCCCCAAGCACTGGGCTAGATGTAGTCAGTGGTGAGACAGACT

AATGTGAATGGCCTGGATGGTGATATGTATGACTATCTTTATGTTGTTGT

TGTTGTTGTTGTTGTTATGTGTGTGGTTTTTTGAGACATGGCTTCTCTGT

GTGGCTCTGTCTGTCTGGGAACTTACTAGTAAACCAGGCTGGCATCAGAC

TCAAAGATATGCCTGCCTCTGTTTCTCAATCACTGGCATTAGAGGTGTGT

GTCACCACTGCCACGCCTTTTCCTTGACATACATTTTTCAAGAAGAAAAT

GTGGATTTTTTTTTTCATTTTTTGACGTAATGATCCTTTCTTTCTTTCTT

TCTTTCTTTCTCCTTTTTTTCTTCGAGACAGGGTTTCTCTGTACAGCCCT

GGTTGTTCTGGAACTCACTTTGTAGACCAGGCTGGCCTCGAACTCAGAAA

TCCGCCTGCCTCTGCCTCCTGAGTGTTGGGATTAAAGGTGTGCGCCACAG

CCCAGCTCCTTTCTTTCTTTCTTTCTTTCTTTTTTTAAAGATTTATTTAT

TTATTATATGTAAGTACACTGTAGCTGTCTTCAGACACTCCAGAAGAGGG

CATCAGATTTCGTTACGGATGGTTGTGAGCCACCATGTGGTTGCTGGGAT

TTGAACTCAGGACCTTCAGAAGAGCAGTCGGCGTTCTTAACCACTGAGCC

ATCTCGCCAGCCCCTCCTTTCTTTTTTTGATTACTTGACTTAACTTGTTC

ACATTACTTGGGTCTAAGAGTCATCCACAAACTACAGAAACAACAATCTC

CATTAGACAGGGATGGTTTATTGCACACACACACACACACACACACACAC

ACACACACACGCACGCACACACACACGCAAACACACACAAATACTGATCA

ATTAGGACCACAGCTCAGAATTTAGAGACTGAGCCACAGCATTTGAATGA

ACATCTTTCTTTATCAGCTTCTAAAGGAAAAAGACCAAAAAACACAAAAA

AACAAGCCACAGTGAGTTCATACACAGGTGCAGGAAGTGCCACCTGGTGG

TCAGCTCTGACTCAAGCCTTTTAGACATAATAGTTCATATTGACCTTGAA

CTTGATGGGTCCTACACATAAAGCTTTGCTTAAGATTAACAAACCTCAAA

ACATACAGAACACAATAGGATGTGTGATCAGCATGACCTTGCTCTGAGTA

AAGTCATTTTTCTGTGTCAATGACTGGCAGGCATGCTAGGGGGGCAGACC

TCAACAGTATGACTGCTTCCCCATGTTCTTCAGACCTTTCTATGTTTTGA

TGGACTGCAAACATTCGTTTTAATTGGCTTCCACAGGATAATCCAGGAGA

AAGAATTAAGTGAATGTCTGAGATTTTTAATGTTGCACAGACTTTGCTTA

ATCCACAACCCTTTAAAGTTGAATATGAAACATTCATTGGAAATCAACTG

GCAGGCCAACTCTCAAAGTGGCACAGTTATTAGCTACCCAAGGAAATGTA

CCATGCCATGCTTATTAGCGTGTCTTCAGTAAAACACCCAAAGGTGCTAG

AGATATGGCTCAATGGTTAAGAACACCAGCTGTCATTACAAAGGACTCAA

GTTCAAGGCCCAGCATTTACATGATGGCTCACAATCATCTGTAACTCTAG

TTCCAGGGGATCCAATACCCTCTCAGGATGCCAGACATGCACACAGTACA

CATACATACATAATACACACACACATATATATATATAGGCAAAACATTCA

AACATATAAAATAAAAAGAATTCTTTTTTTTTAAAGGTACATTGGGGGGG

GGGGGTTCGAGACAGGGTTTCTCGGTATAGTCCTGGCTGTCCTGGAACTC

ACTCTGTAGACCAGGCTGGCCTCGAACTCAGAAATCAGCCTGCCTCTACC

TCCCAAGTGCTGGAATTAAAGGCGTGTGCCACCACCGCCCAGCAAGGTAC

ATTATTTTTAAGATTTGTTTATTTTTTTATGTATATGACTGCTGTATTGG

CATGTACACCTGTATGCCAGAAGAAGGCCTCAGATTATATTAGAGATGGC

TGGGAGCCACCTTGTGGTGCTAGGAATTGAATTCAAGACTTTTGGAAGAG

CAGACAGTGCTCTTAACATGTGAGCCATTTTTCCAGCCCAATAAGTAATT

AAAAAAAAAAAAAAGCTGGGCAGTGATGGCGCACGCCTTTAATCCCAGCA

CTTGGGAGGCAGAGACAGGCAGATTTCTGAGGTCGAGGCCAGCCTGGTCT

ACAAAGTGAGTTCCAGGACAGCCAAGACTATACAGAGAAACCCTGTCTCG

AAAAACAAACAAAACAAAACAAAACAAAACAAAACAAAAAGTTTGGTGAA

TAAGCTTGAAGATTTGAGTTCAAATCCCCAACACCTAAGTGCCTGCCTGT

AACCACAGCAGGACAGAAGCAAGAGGGGCTCACTGGGTCCTCTTAGCCAC

CAGCCAAGCTCCAGGTTCAATGAAAGATCCTGTCTCAAGGGAATAAGGTA

GAGCGTGATAGATGAGGGTACCCAATGTTCTCTAGAACAGCGCTTCTTAA

CCTTCCTAATGCTGCAGGCCTTTAATATAGTCCCTCATGTTGTGGTGACC

TCCAACCACAAAATTATTTTTGTTGTTGTTTCATTACTGTATTTTTGCTA

CTGTTATGAATTGTAATATAAATATCTGATATGCAGGATATCTGATGTGT

GACCTCTGTAAAAGGGTCATTTGGCCCCCCAAAGGATCAAACCCACAGCT

TGAGAATCGCTGCTCTAGGCTCTTGGGTGCATGCACAGGTGTGTGCTCCT

AAACATATGCACATGTATCACACATGCACACACACACACACACACACACA

CACAGGAGAACAAGAGGTGTTGATGGGGAATTGTGAAGTTGGTATTAGAA

TCCATCACAGTCCACCTCCTCGCCTTTGCTATAGGATTAAGCAAGAGACT

CACAGAACTACTGGGTCAAAGAACATTGTTCCCAGCTGGTGTGGAGGTGC

ACACCTTTAATCCCAATATTCAGGAGGCAGAAGGAAGCAGATCTCTGTGA

CTTAGTGGCCAGCCTGGTCTACAAAGAATGTTTCAGGACAGCTAGTGCTA

CACAGAGAAGCCCTGTCTCAAAAAACCCAGATGGTGGTGGTGGTAATGAA

TGAAATTGTTCCTATGAATATCCATATAAAAATGCCCAAGCAGGCAGATC

TCTGAGTTTGAGGCCAGCCTAGTCTATGGAGCCAGCTCCAGGACAGCTAA

GGTTACATTAAGAAACCCTTACTCCAAAAACCAAAAGAGAAGCCGGGCGT

GGTGGCACATGCCTTTAATCCCAGCACTTGGGAGGCAGAGGCAGGTGGAT

TTCTGAGTTCGAGGCCAGCCTGGTCTACAAAGTGAGTTCCAGGACAGCCA

GGGCTGTATAGAGAAACCCTGTCTTGAAAGAAACAAAAAAACAAAAACAA

AAAAAAAACAAAAAACAAAAAAAACAAGAGAGAGAAAGGAGGAAAGTGTG

AGAGAAATACTCAGCCTGAACCTGTTAATAAGCTGTATTTTAAAATAATT

GTGGAAAAGCAGACCAAAGGCAAATGGAAGGCAGTCATGGAAAACTATAG

AACTCTAGGTTATCTTTATCCCAAAGATTTTGAACCGCATTACTACGTGG

ACCTACAGGACCAATGGATAAATGCTTACCAAGCAAAAAGTTTCCATTGT

AAGGGAAACCTAACAAGGATTTAAGAAACTTCTTTCCCTAGGAGGTGTCC

CTGGGAATGTAGGAATTATAATGCTCTTGGTCAAGCCTAGTTTGCCTTCT

TCTCCCTATGTGTAAAAATATACATAAAAATTTAACTGGAGTATTTTGCT

TCTCCAGTAGCTGTTCTGACAGCAGCTCCTGATCTGGGGAAGAAGTCAGA

CCCTGGACATGGCAGTGTCTTTTGTAGCTCTAATGTTTTGTTCCTCTCAT

ACCCTACATATGTTTTATGATTTCATATTTAGATTTAAATCCATGGCAAT

GGGCTGGTGAGATGGCTCAGTGGGTAAGAGCACCCGACTGCTCTTCCGAA

GGTCCAGAGTTCAAATCCCAGCAACCACATGGTGGCTCACAACCATCCGT

AAGGAGATCTGACTCCCTCTTCTGGAGTGTCTGAAGACAGCTACAATGTA

CTTCCATATAATAAATAAATAAATCTAAAAAAAAAAAAAAATCCATGGTG

AACAGCATATTCAATTCAAAGATCACCCCAACTGTTTCTCCAGACACCAA

GATGGAAAAGATGGGGGCGCAAAAACCACGTCACACCCATCAAAACAAAC

TGGGTTATAGTTAGGCCCCAATAAATACCCTGAAACTGAATCTCATTTCT

CTTCTCTTCTAAGCTTCCGCAGGTTCACTCGGTCCAACAGTGTGACAACT

GCAGTACAGGCCGACCTGGACTTCCATGATAATCTGGAAAATTCTCTGGA

GTCTATAGAGGACAATTCGTGTCCTGGCCCCATGGCCAGACAGTTCTCGC

GGGACGCCAGCACCTCTACCGTCAGTATCCAGGGTTCAGGAAACCACTAT

CATGCCTGTGCAGCCGATGACGACTTTGACACGGATTTTGACCCCTCTAT

CCTGCCTCCTCCGGACCCCTGGATTGACTCTATCACAGAAGACCCTCTGG

AGGCCGTTCAAAGGTCCGTGTGCCACCGGGACGGCCACTGGTTCCTGAAG

CTTCTCCAGGCAGAGCGGGACCGCATGGAAGGGTGGTGCAAGCTAATGGA

GAGAGAAGAGAGGGAGAACAACCTCCCAGAAGACA
