# Supplementary material for: Circ_0000811 acts as a miR-15b sponge and inhibits Prkar2a-mediated JAK2/STAT1 pathway to attenuate cerebral ischemic vertigo
Source: Cell Death Discov. 2022 May 4;8:247. doi: 10.1038/s41420-022-01016-2 (PMC9068921; doi:10.1038/s41420-022-01016-2)
Supplement: Supplementary file 3 — Supplementary Table 2 [file 41420_2022_1016_MOESM3_ESM.docx]

**Supplementary Table 2 Primer sequences for qRT-PCR of miRNAs that bind to circ_0000811**

| Genes | Primer sequences |
| --- | --- |
| miR-471-3p | Forward: 5'-GCCGACTGAAAGGTGCCATACT-3'  Reverse: 5'-CAGTGCGTGTCGTGGAGT-3' |
| miR-488-3p (mouse) | Forward: 5'-GCGCAGTTGAAAGGCTGTTT-3'  Reverse: 5'-AGTGCGTGTCGTGGAGTCG-3' |
| miR-326-3p (mouse) | Forward: 5'-CCTCTGGGCCCTTCCTCCAGTGT-3'  Reverse: 5'-GTGCAGGGTCCGAGGT-3' |
| miR-330-5p (mouse) | Forward: 5'-CTGATCTCTGGGCCTGTGTC-3'  Reverse: 5'-GTGCAGGGTCCGAGGT-3' |
| miR-1247-5p (mouse) | Forward: 5'-GGGACCCGTCCCGTTCGTCC-3'  Reverse: 5'-CAGTGCGTGTCGTGGAGT-3' |
| miR-322-5p (mouse) | Forward: 5'-CAGCAGCAATTCATGTTTTGGA-3'  Reverse: 5'-GTGCAGGGTCCGAGGT-3' |
| miR-103-3p (mouse) | Forward: 5'-ACACTCCAGCTGGGAGCAGCATTGTAC-3'  Reverse: 5'-TGGTGTCGTGGAGTCG-3' |
| miR-107-3p (mouse) | Forward: 5'-ATTGCGGAGCAGCATTGTACAGG-3'  Reverse: 5'-ATCCAGTGCAGGGTCCGAGG-3' |
| miR-6342 | Forward: 5'-GCCGACCAGCAGCAATCTGGTC-3'  Reverse: 5'-CAGTGCGTGTCGTGGAGT-3' |
| miR-6419 | Forward: 5'-GCCGACCAGCAGCAATCTGACA-3'  Reverse: 5'-CAGTGCGTGTCGTGGAGT-3' |
| miR-1955-3p | Forward: 5'-GCCGACGAGCATTGCATGCTG-3'  Reverse: 5'-CAGTGCGTGTCGTGGAGT-3' |
| miR-15b-5p (mouse) | Forward: 5'-TGAGATGAAGCACTGTAGCTC-3'  Reverse: 5'-GCTACAGTGCTTCATCTCATT-3' |
| miR-15a-5p | Forward: 5'-GCCGACTAGCAGCACATAATGG-3'  Reverse: 5'-CAGTGCGTGTCGTGGAGT-3' |
| miR-497a-5p (mouse) | Forward: 5'-CAGCAGCACACUGUGGUUUGUA-3'  Reverse: 5'-CAAACCACAGUGUGCUGCUGUU-3' |
| miR-195b | Forward: 5'-GCCGACTAGCAGCACAGAAATA-3'  Reverse: 5'-CAGTGCGTGTCGTGGAGT-3' |
| miR-6353 | Forward: 5'-GCCGACTAGCAGCACGTATTTAT-3'  Reverse: 5'-CAGTGCGTGTCGTGGAGT-3' |
| miR-1907 | Forward: 5'-GCCGACGAGCAGCAGAGGATCT-3'  Reverse: 5'-CAGTGCGTGTCGTGGAGT-3' |
| miR-16-5p (mouse) | Forward: 5'-TAGCAGCACGTAAATATTGGCG-3'  Reverse: 5'-TGCGTGTCGTGGAGTC-3' |
| miR-542-3p (mouse) | Forward: 5'-TCGGGGATCATCATGTCACG-3'  Reverse: 5'-GAGTGGCTCCCAGACCTTTC-3' |
| miR-195a-5p | Forward: 5'-GCCGACTAGCAGCACAGAAAT-3'  Reverse: 5'-CAGTGCGTGTCGTGGAGT-3' |
| miR-140-5p (mouse) | Forward: 5'-CGCATGGCAGTGGTTTTAC CCTA-3'  Reverse: 5'-ATCCAGTGCAGGGTCCGAGG-3' |
| miR-876-3p | Forward: 5'-GCCGACTAGTGGTTTACAAAGT-3'  Reverse: 5'-CAGTGCGTGTCGTGGAGT-3' |
| U6 (mouse) | Forward: 5'-CTCGCTTCGGCAGCACA-3'  Reverse: 5'-AACGCTTCACGAATTTGCGT-3' |
