# Supplementary material for: Circ_0000811 acts as a miR-15b sponge and inhibits Prkar2a-mediated JAK2/STAT1 pathway to attenuate cerebral ischemic vertigo
Source: Cell Death Discov. 2022 May 4;8:247. doi: 10.1038/s41420-022-01016-2 (PMC9068921; doi:10.1038/s41420-022-01016-2)
Supplement: Supplementary file 4 — Supplementary Table 3 [file 41420_2022_1016_MOESM4_ESM.docx]

**Supplementary Table 3** **Primer sequences for qRT-PCR**

| Genes | Primer sequence |
| --- | --- |
| circ_0000811 (mouse) | Forward: 5'-CTGGAGGCCGTTCAAAGGT-3' |
|  | Reverse: 5'-GGGAGGTTGTTCTCCCTCTCT-3' |
| GAPDH (mouse) | Forward: 5'-AAGCCCATCACCATCTTCCAGGAG-3' |
|  | Reverse: 5'-AGCCCTTCCACAATGCCAAAG-3' |
| miR-15b (mouse) | Forward: 5'-TGAGATGAAGCACTGTAGCTC-3' |
|  | Reverse: 5'-GCTACAGTGCTTCATCTCATT-3' |
| U6 (mouse) | Forward: 5'-CTCGCTTCGGCAGCACA-3' |
|  | Reverse: 5'-AACGCTTCACGAATTTGCGT-3' |

**Note**: miR-15b, microRNA-15b; GAPDH, glyceraldehyde-3-phosphate dehydrogenase.
