# Supplementary material for: Circ_0000811 acts as a miR-15b sponge and inhibits Prkar2a-mediated JAK2/STAT1 pathway to attenuate cerebral ischemic vertigo
Source: Cell Death Discov. 2022 May 4;8:247. doi: 10.1038/s41420-022-01016-2 (PMC9068921; doi:10.1038/s41420-022-01016-2)

Supplementary Figure 3A

Prkar2a

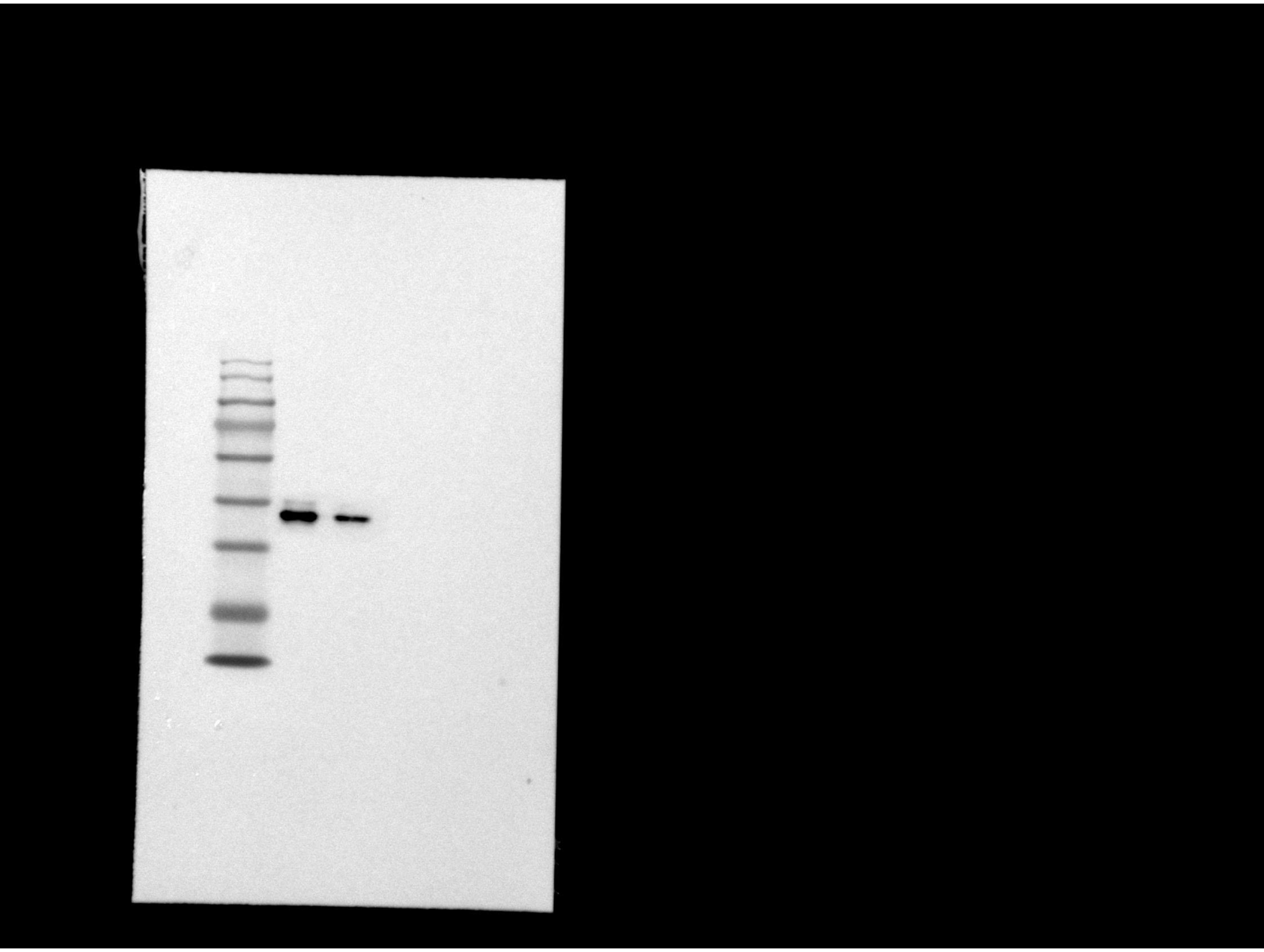

Supplementary Figure 3A

GAPDH

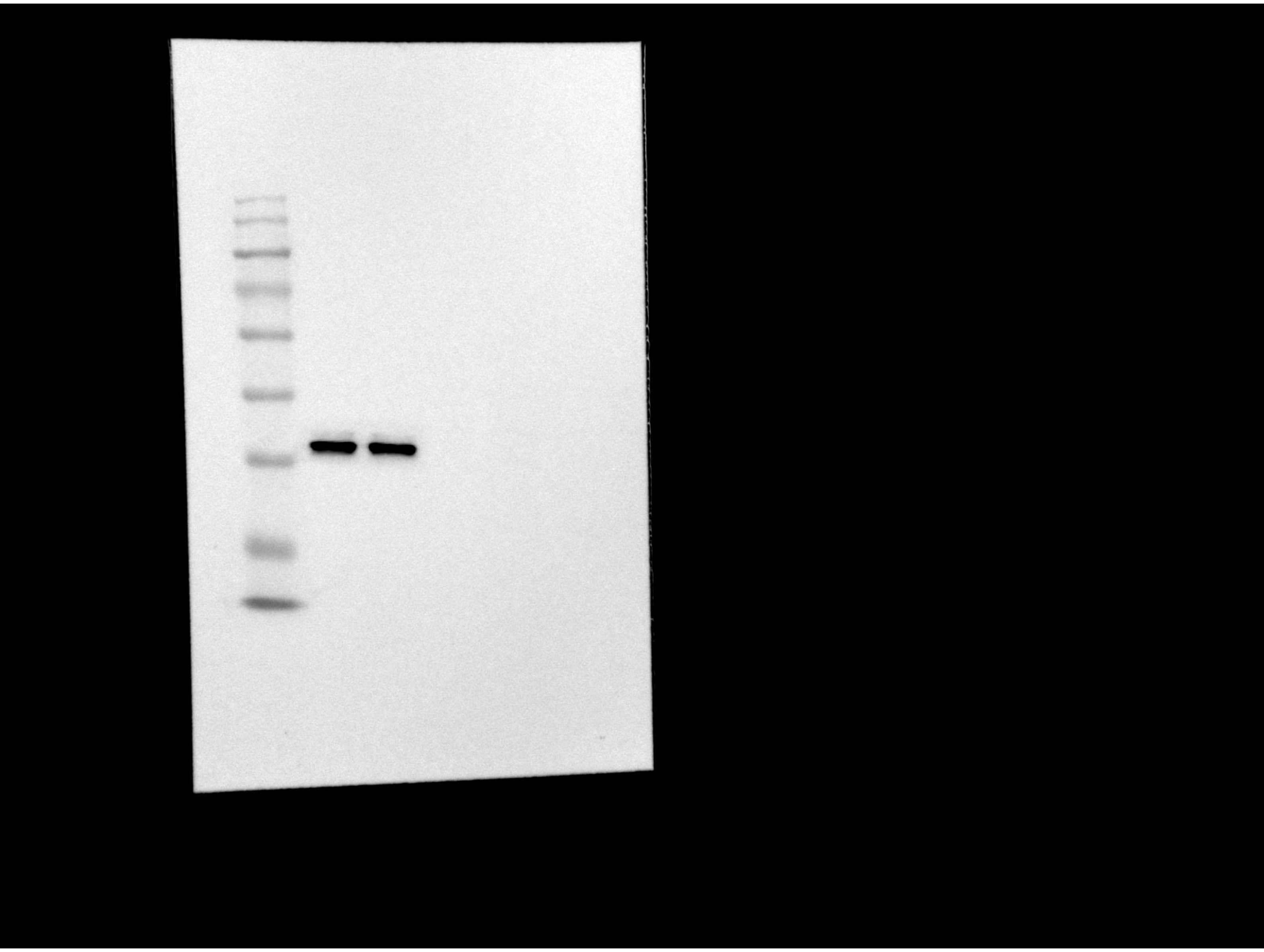

Supplementary Figure 3B

Prkar2a

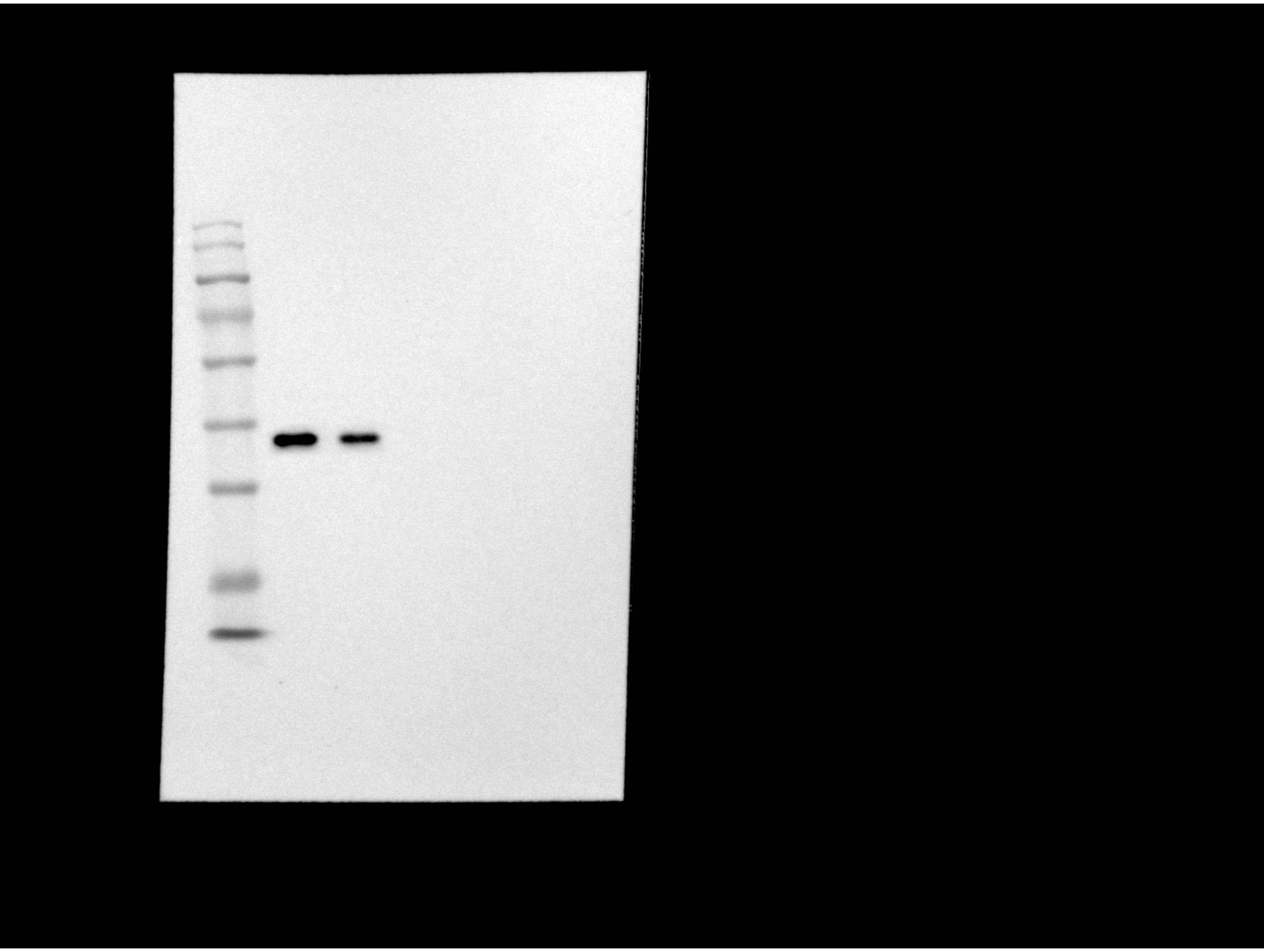

Supplementary Figure 3B

GAPDH

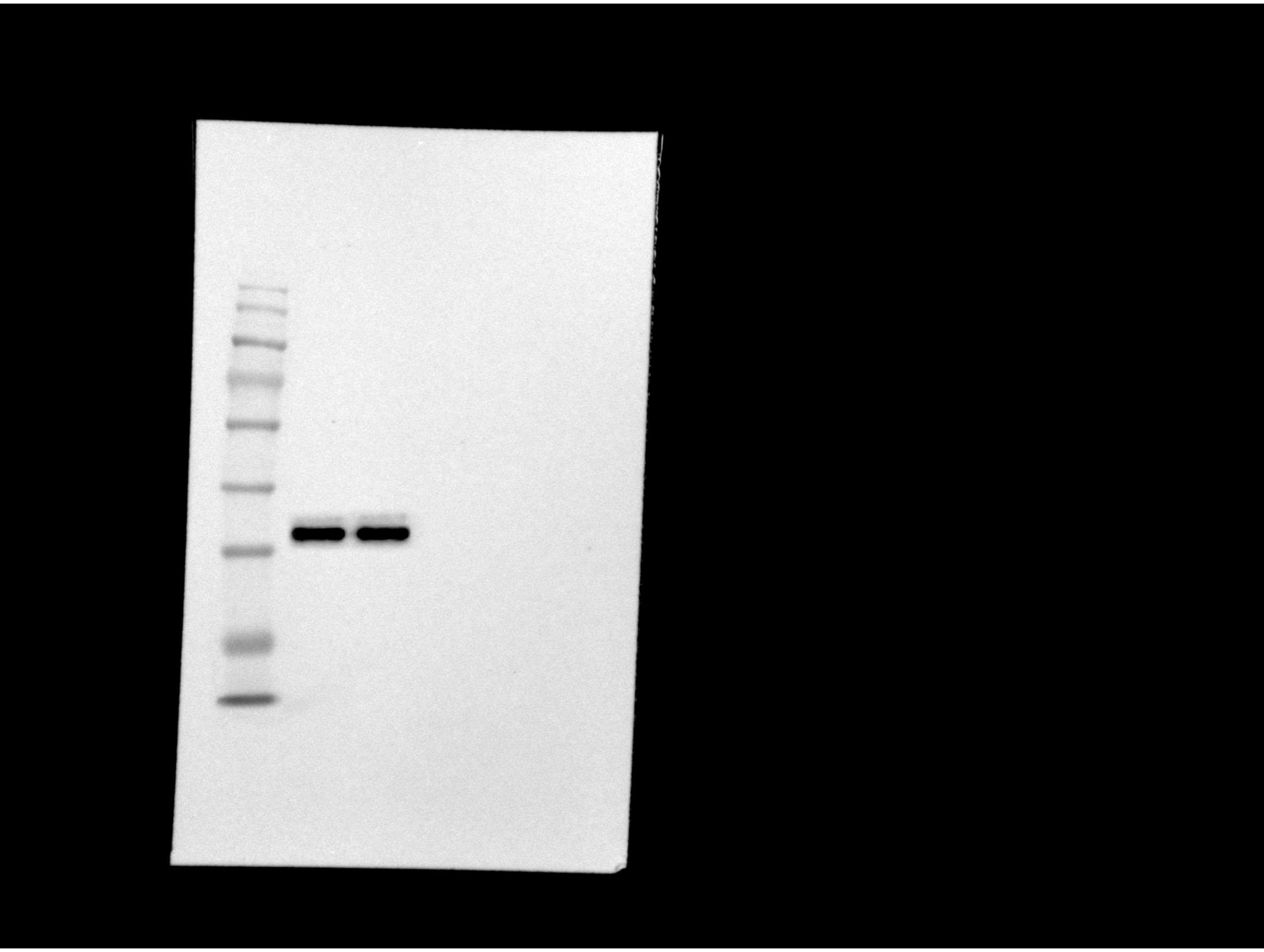

Supplementary Figure 3C

Prkar2a

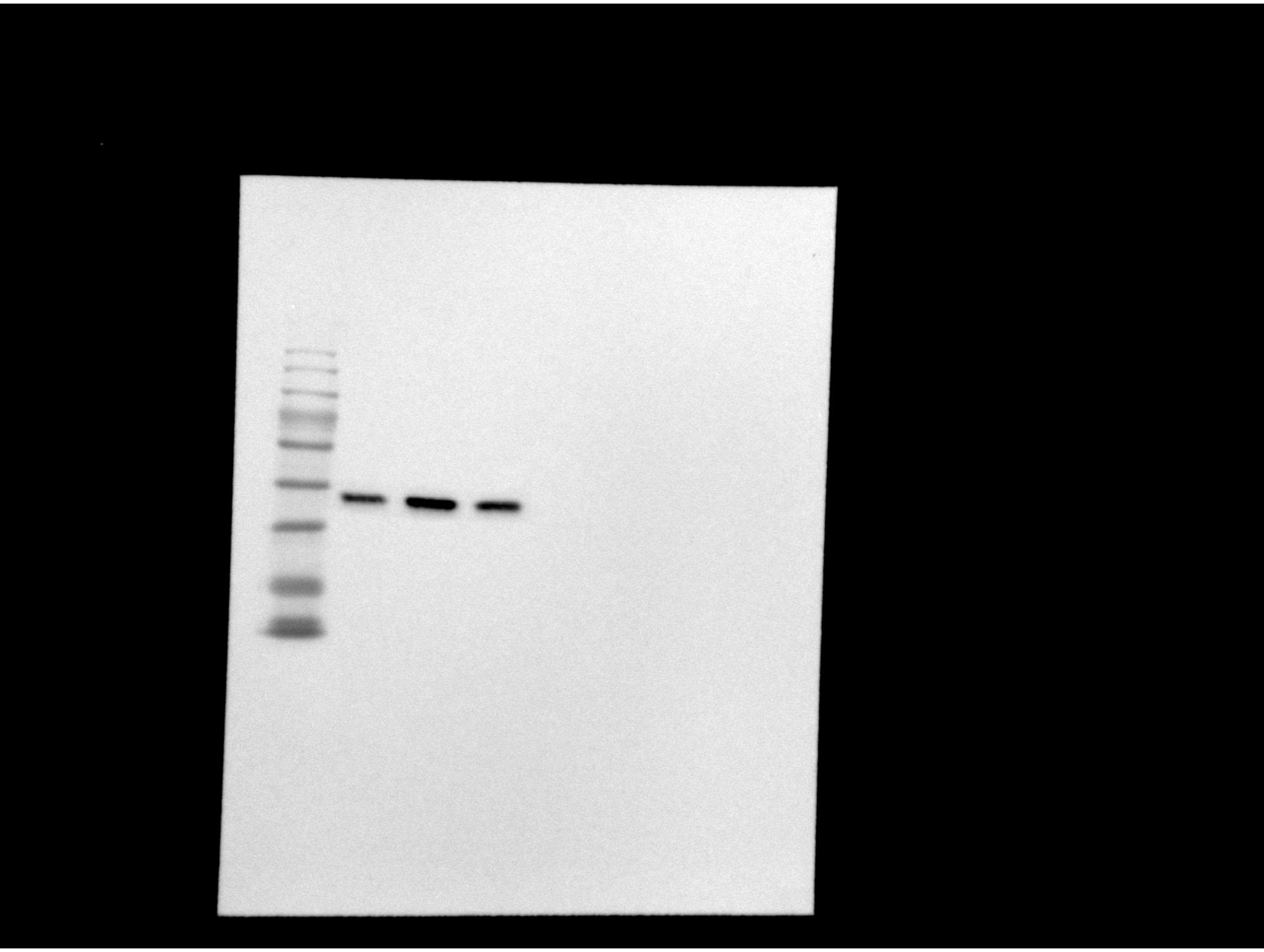

Supplementary Figure 3C

GAPDH

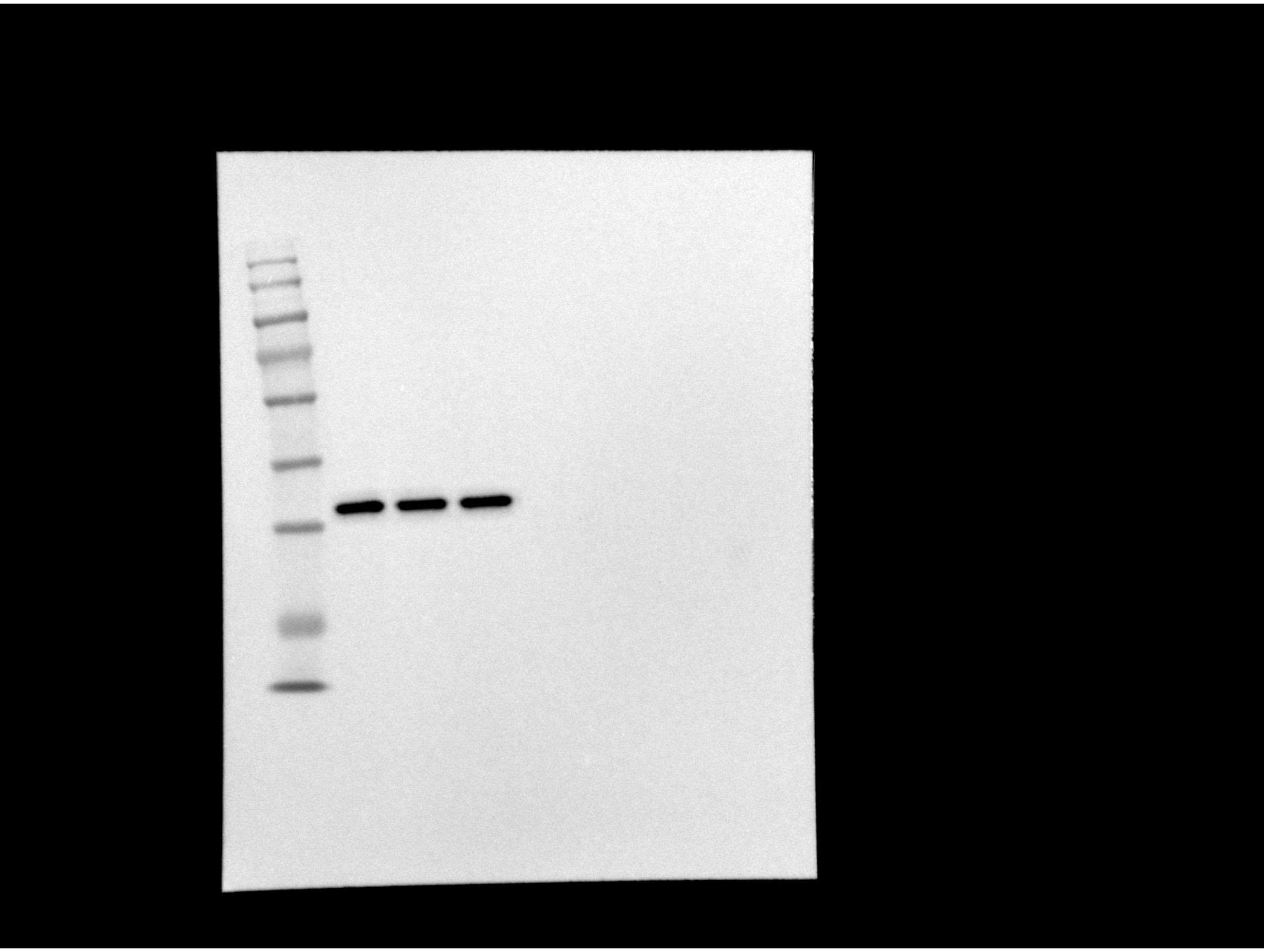

Supplementary Figure 3D

p-JAK2

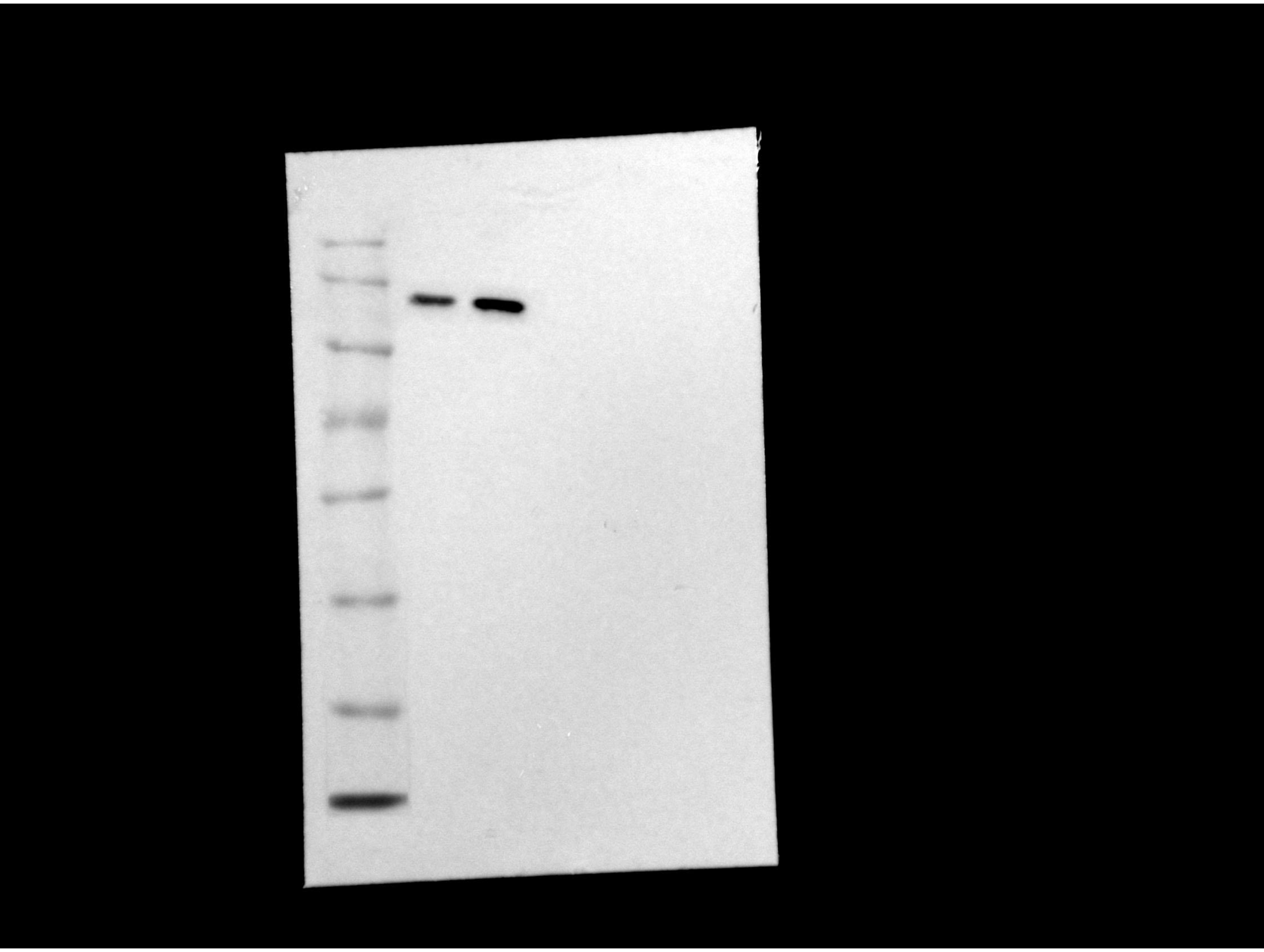

Supplementary Figure 3D

JAK2

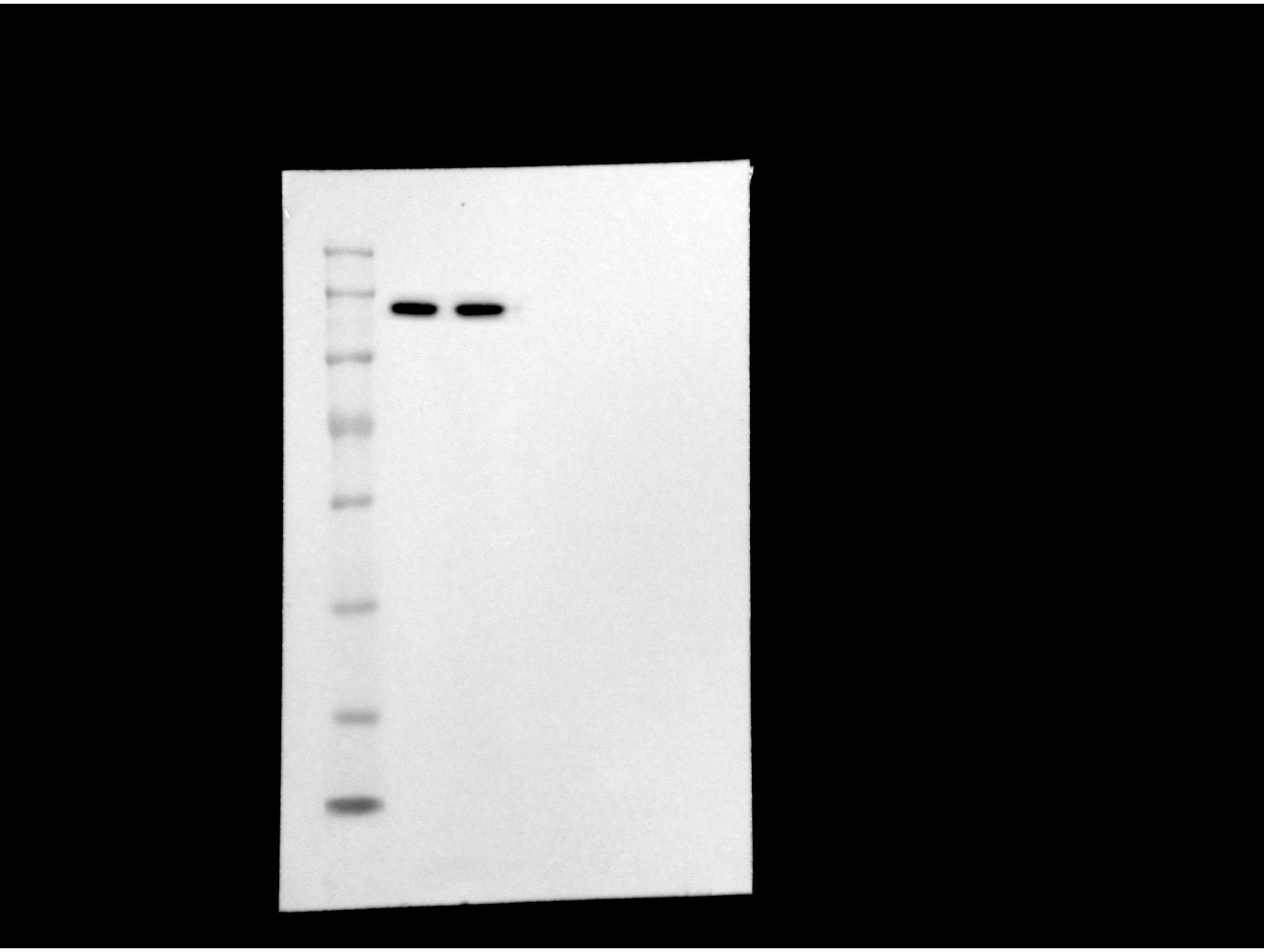

Supplementary Figure 3D

p-STAT1

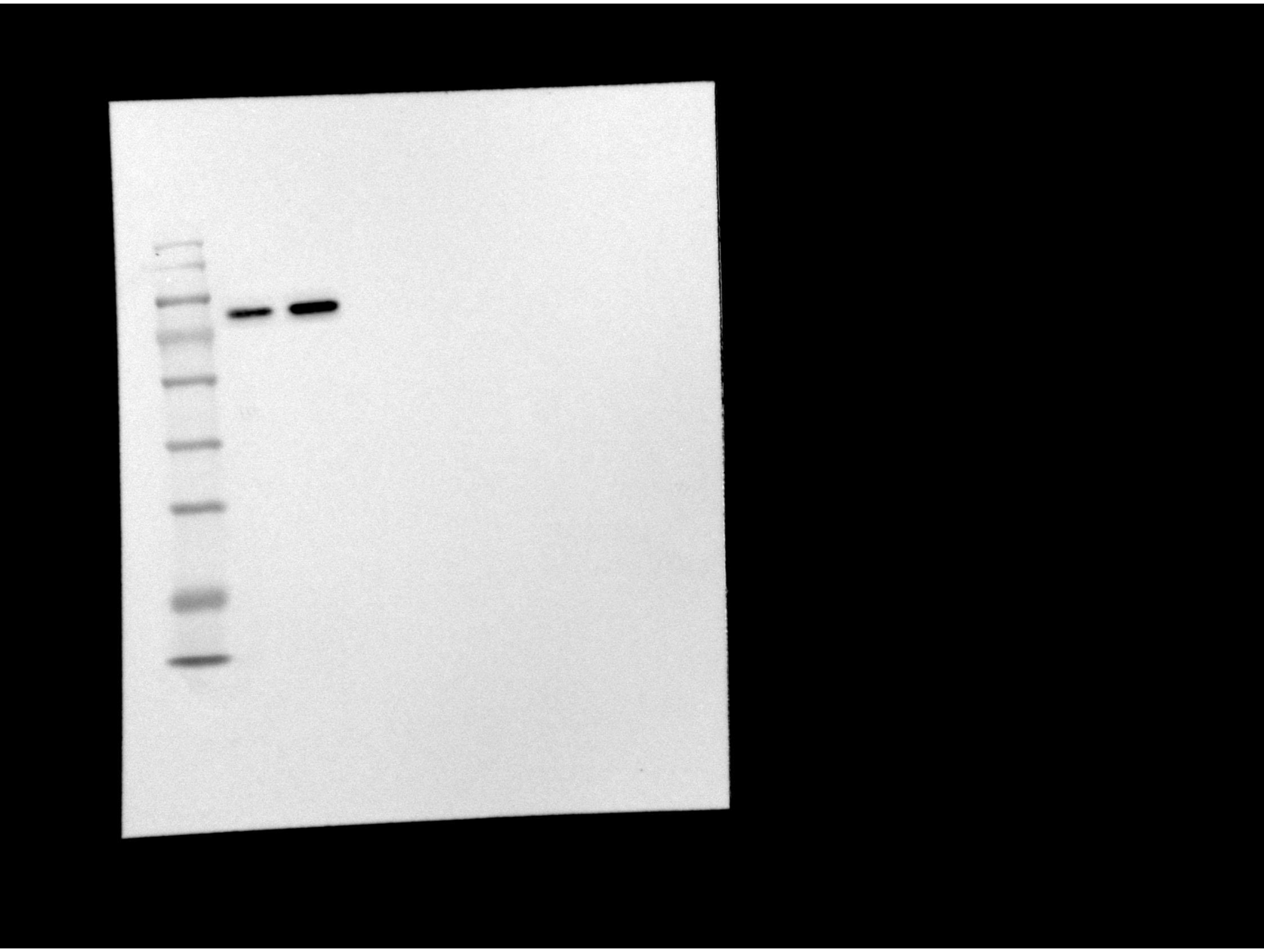

Supplementary Figure 3D

STAT1

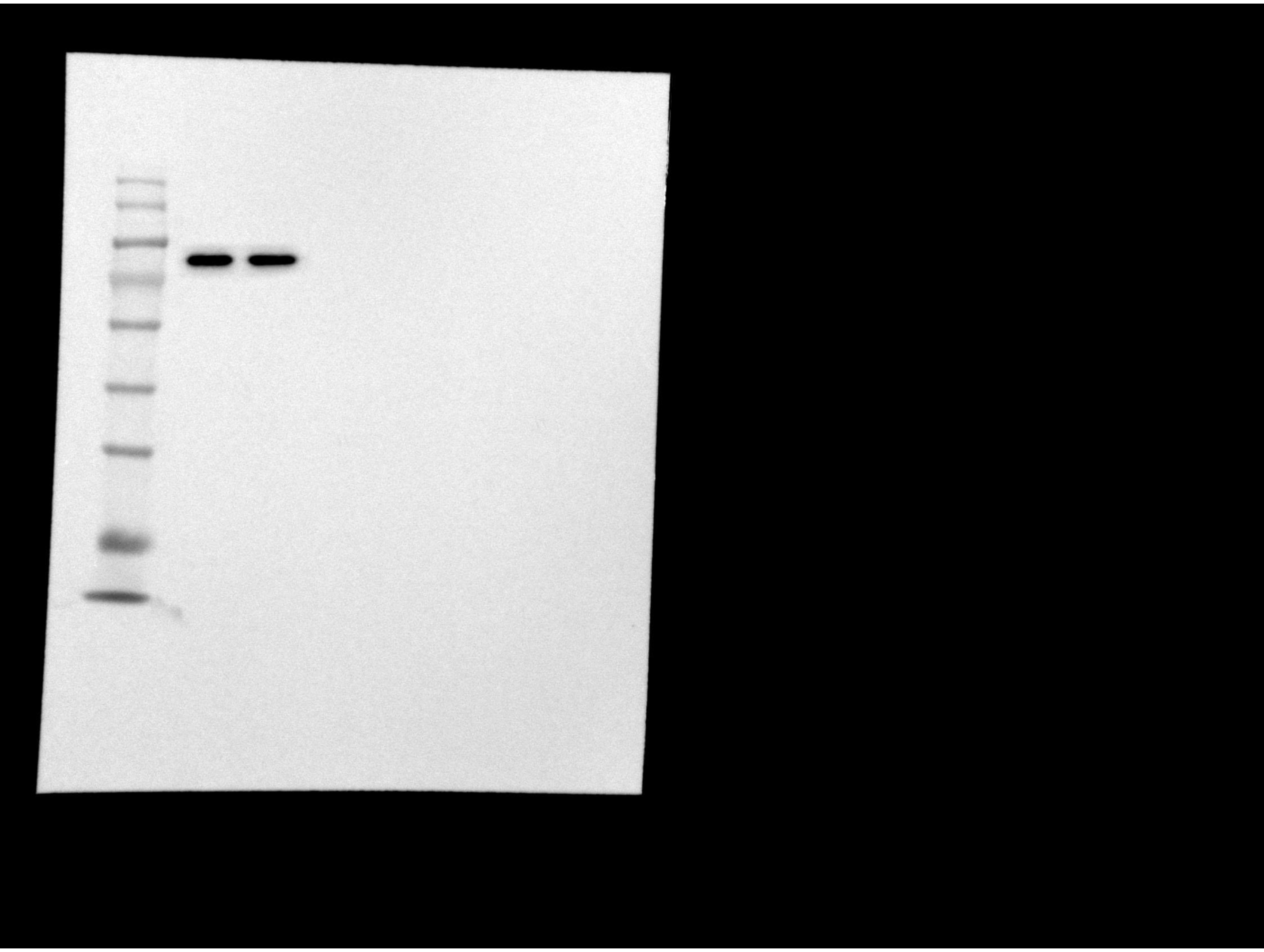

Supplementary Figure 3D

GAPDH

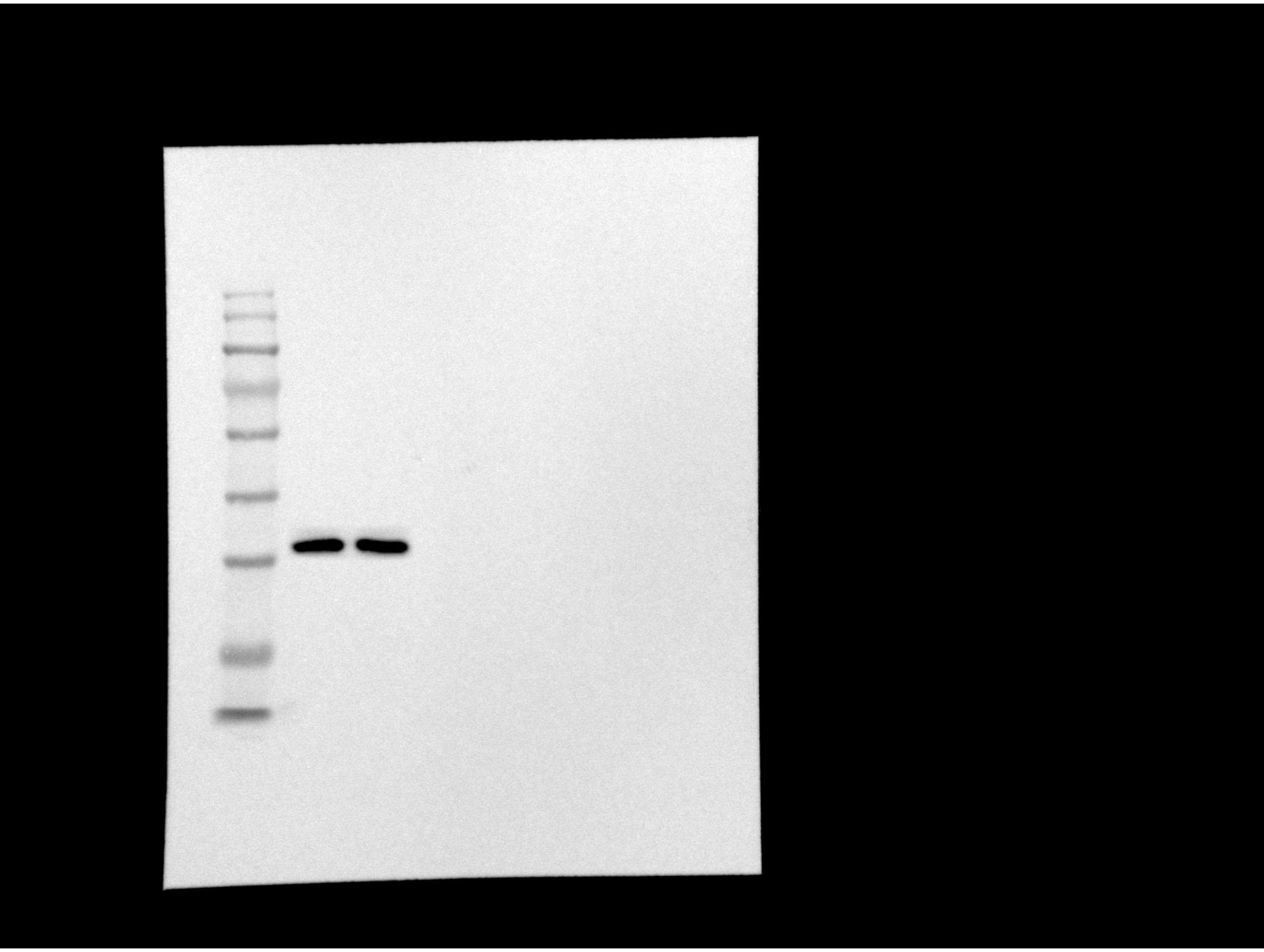

Supplementary Figure 3E

p-JAK2

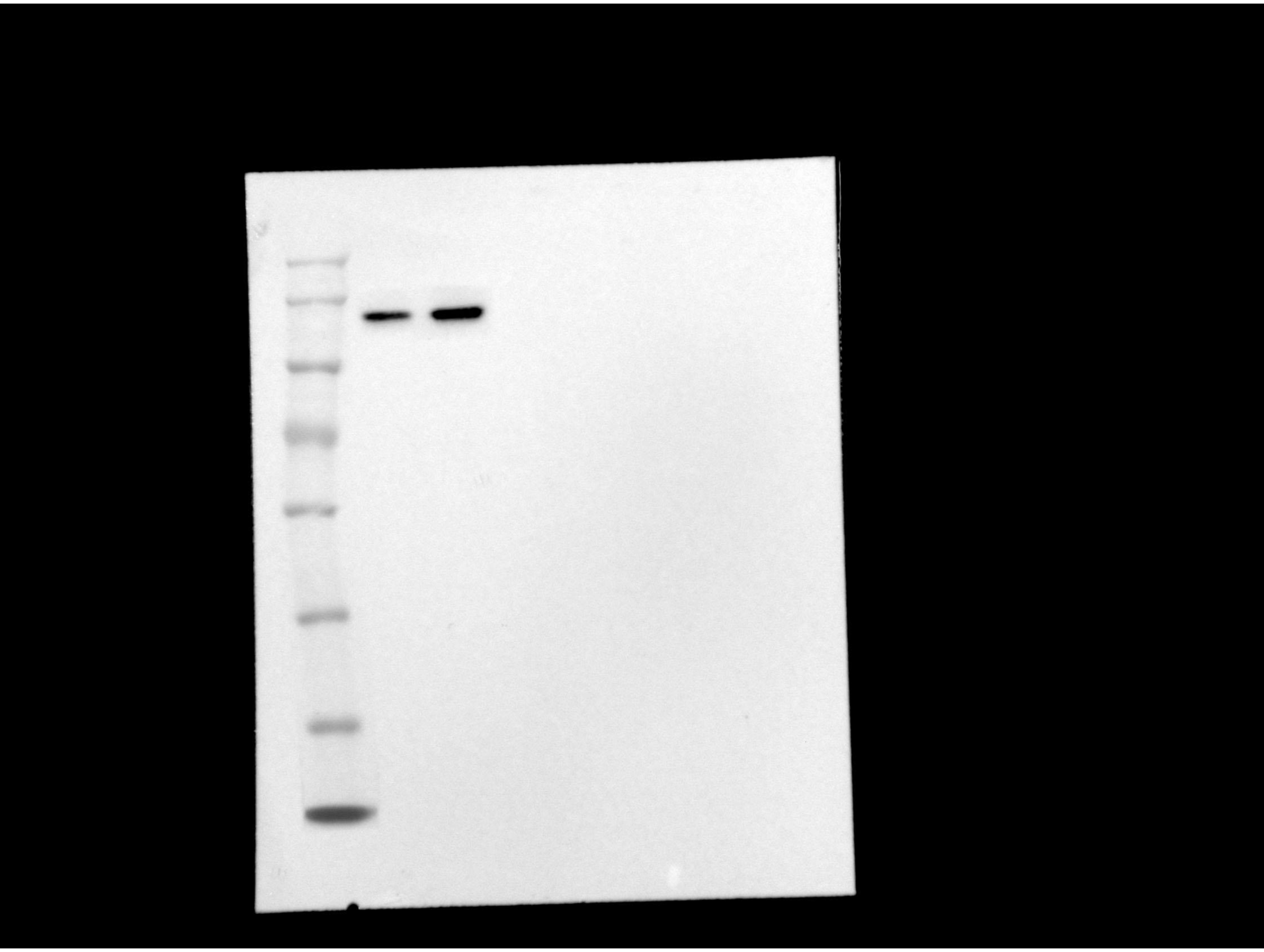

Supplementary Figure 3E

JAK2

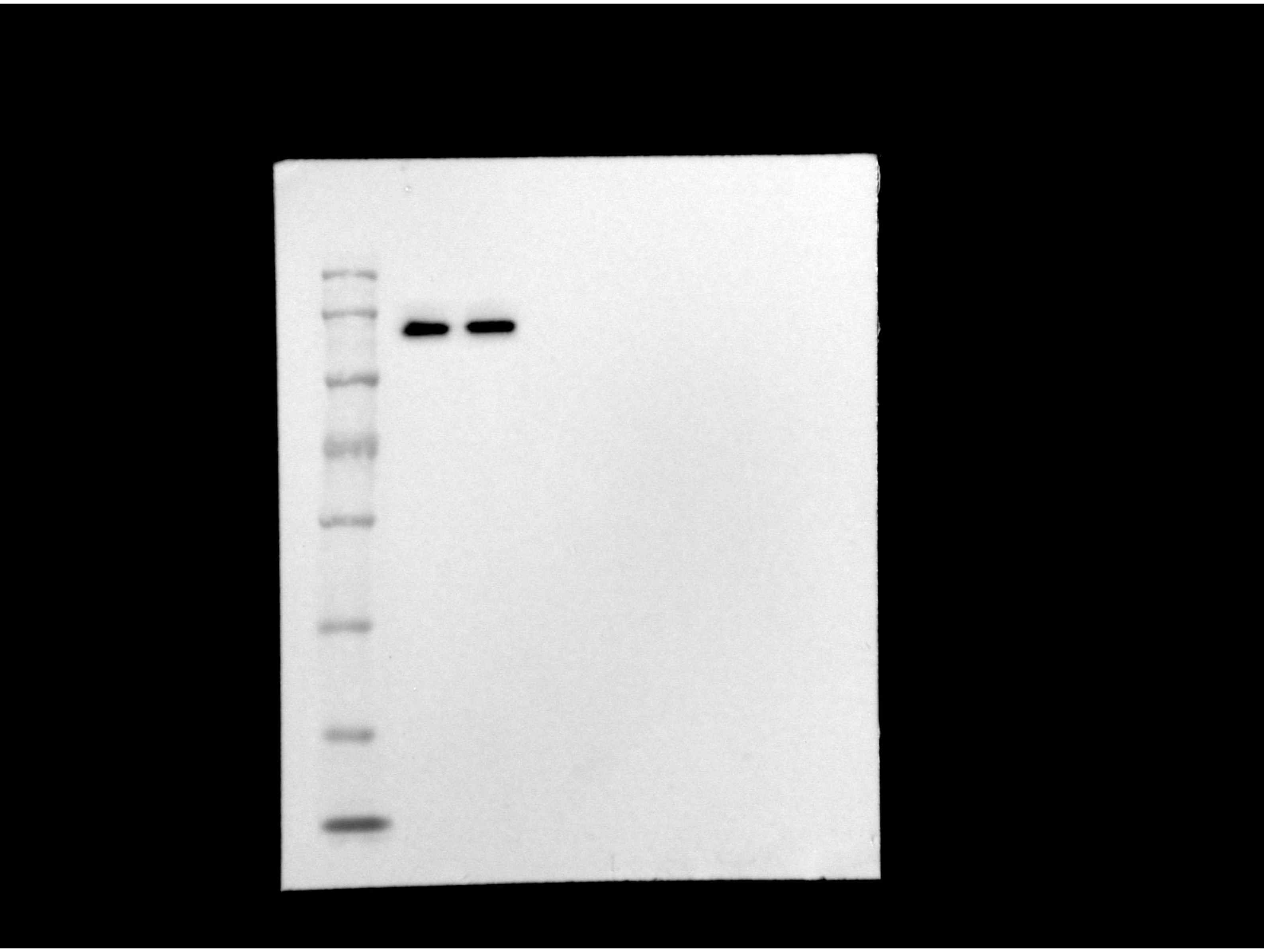

Supplementary Figure 3E

p-STAT1

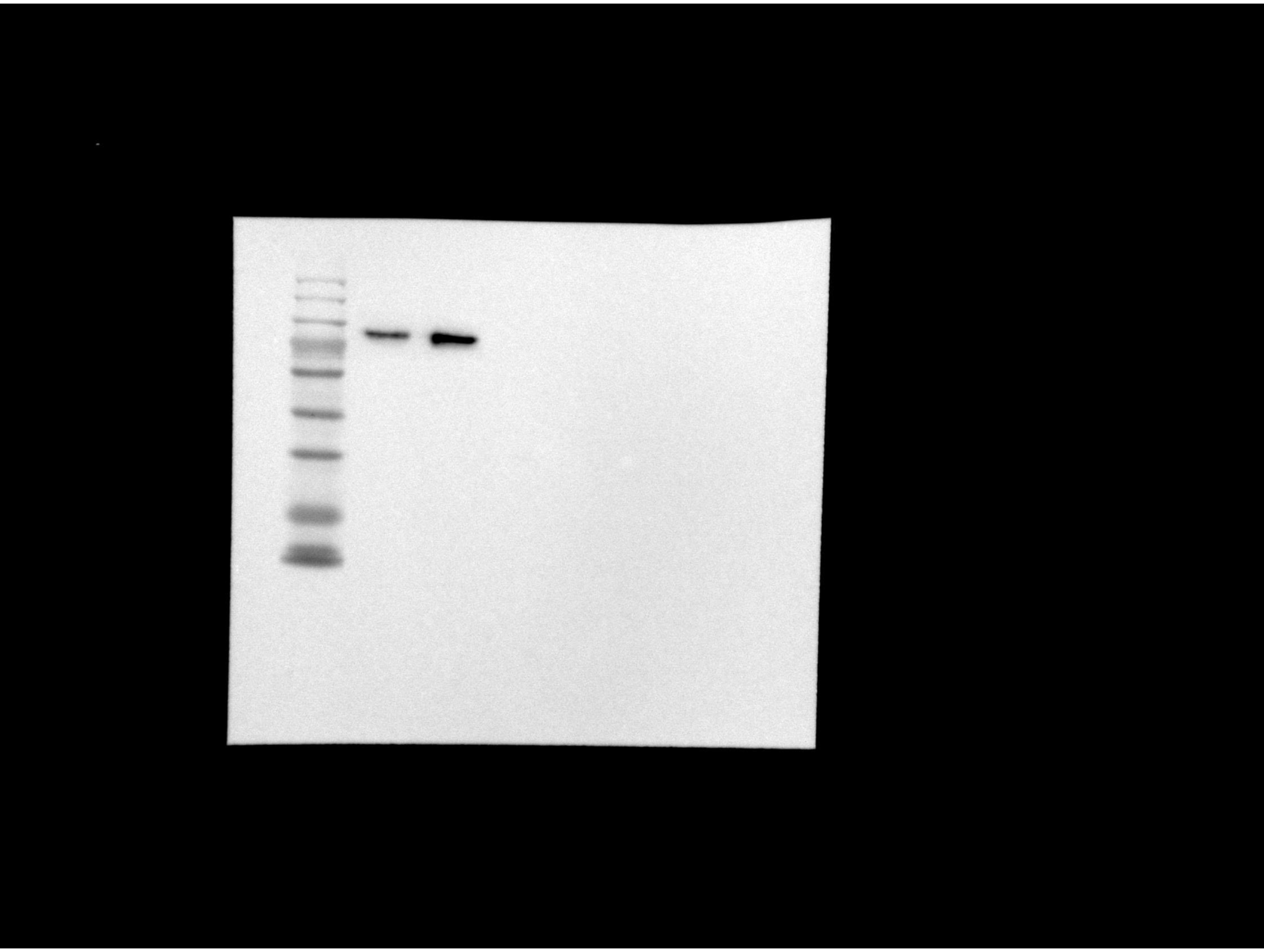

Supplementary Figure 3E

STAT1

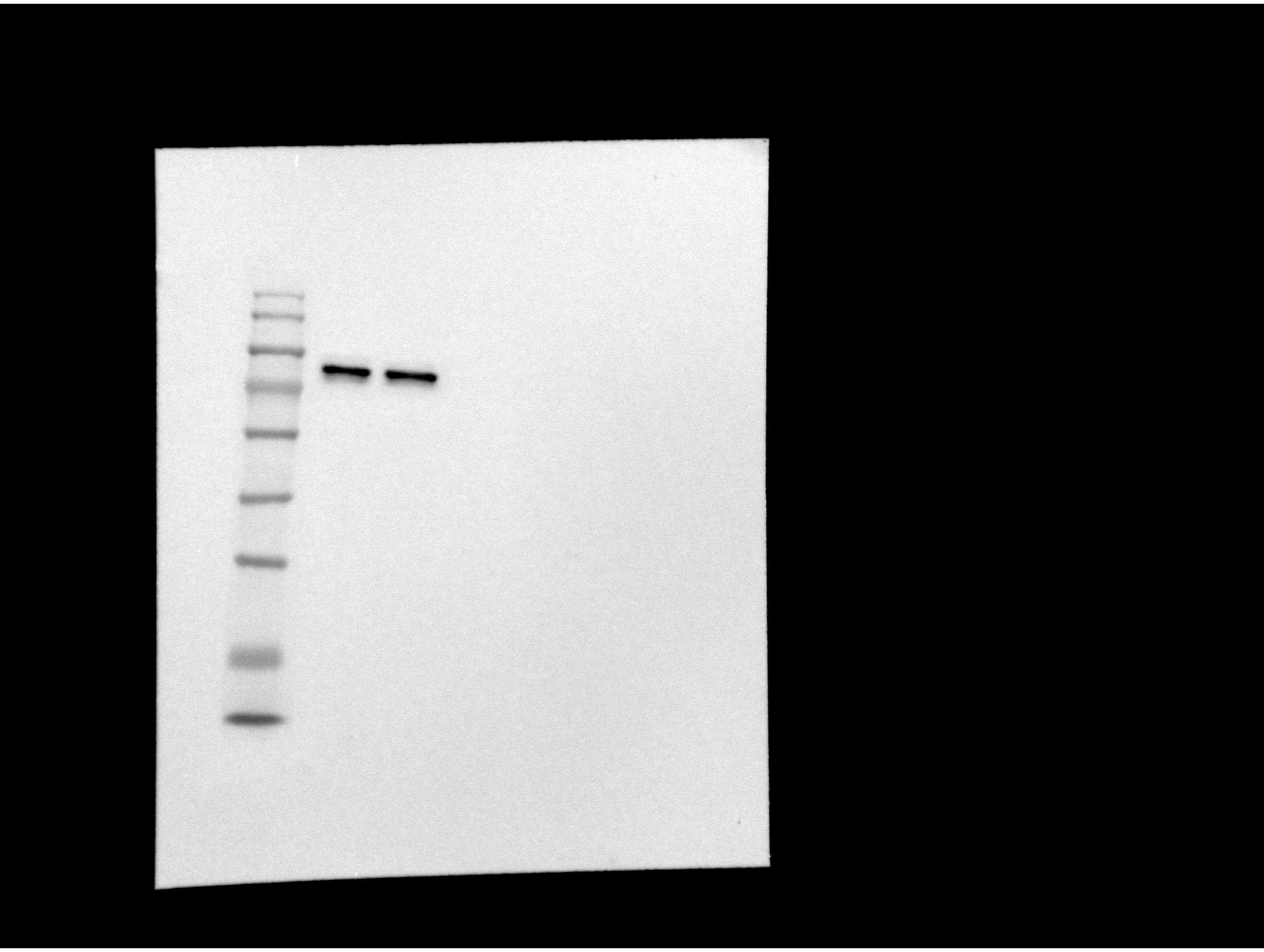

Supplementary Figure 3E

GAPDH

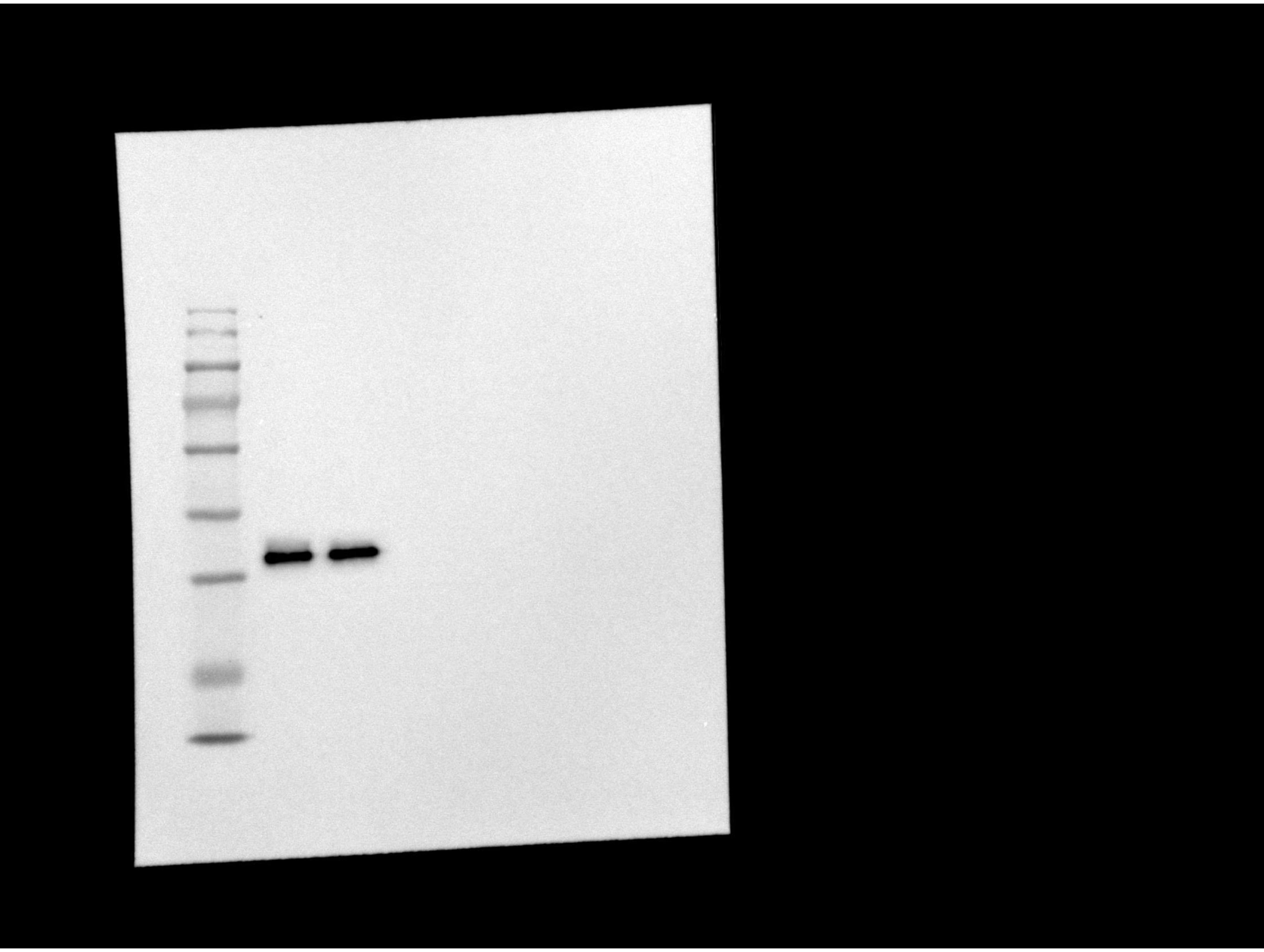

Supplementary Figure 3F

Prkar2a

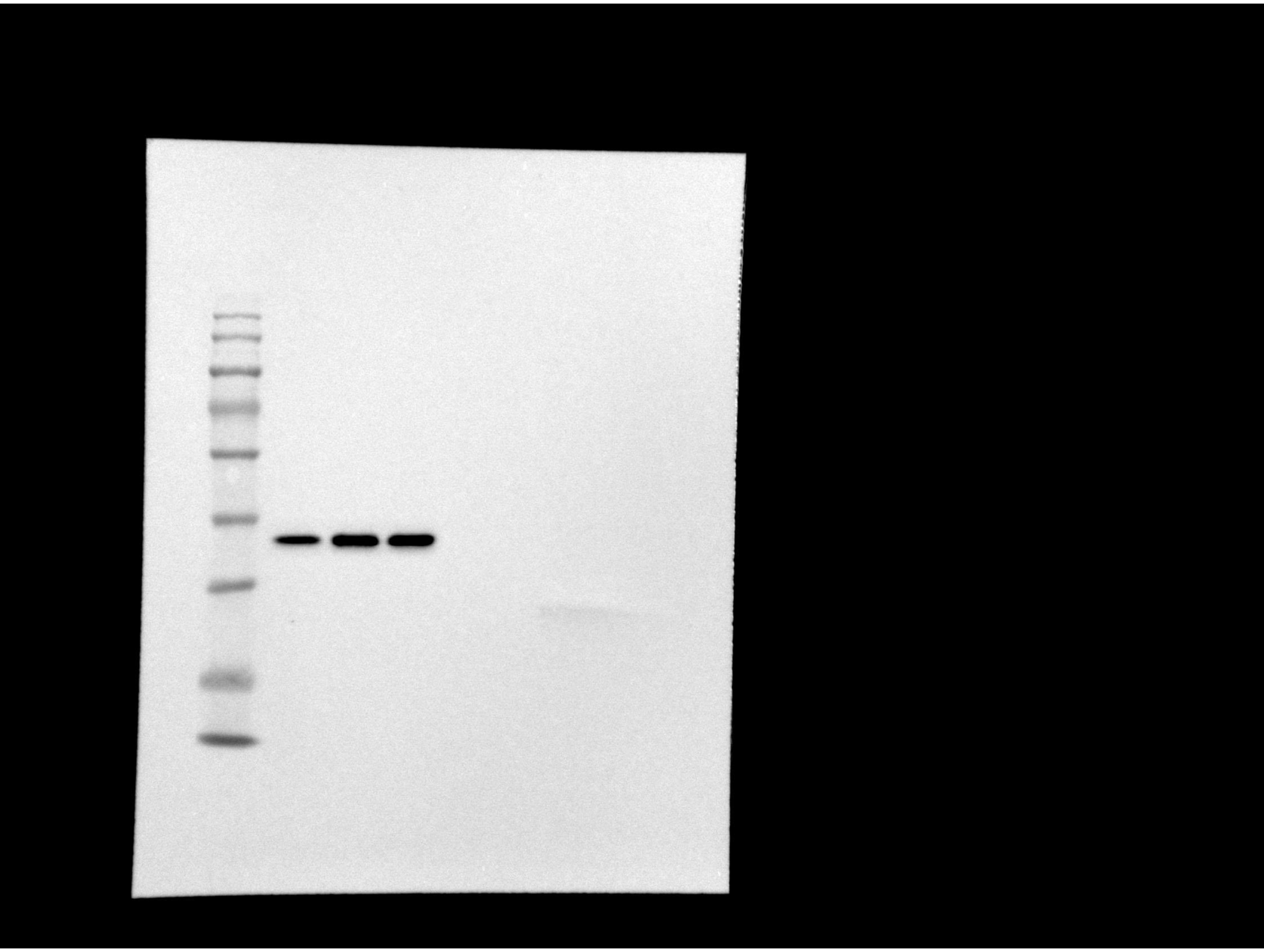

Supplementary Figure 3F

p-STAT1

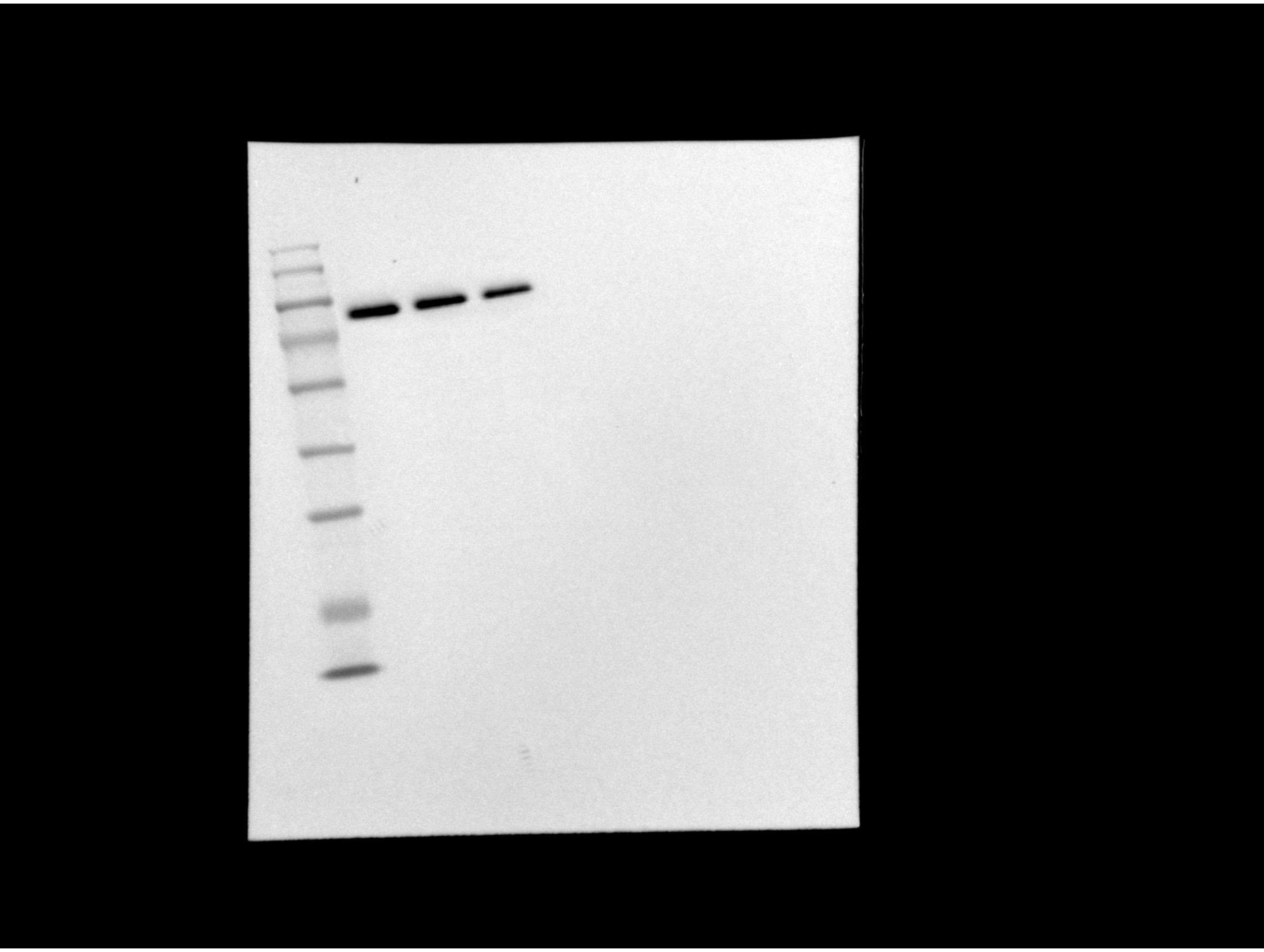

Supplementary Figure 3F

STAT1

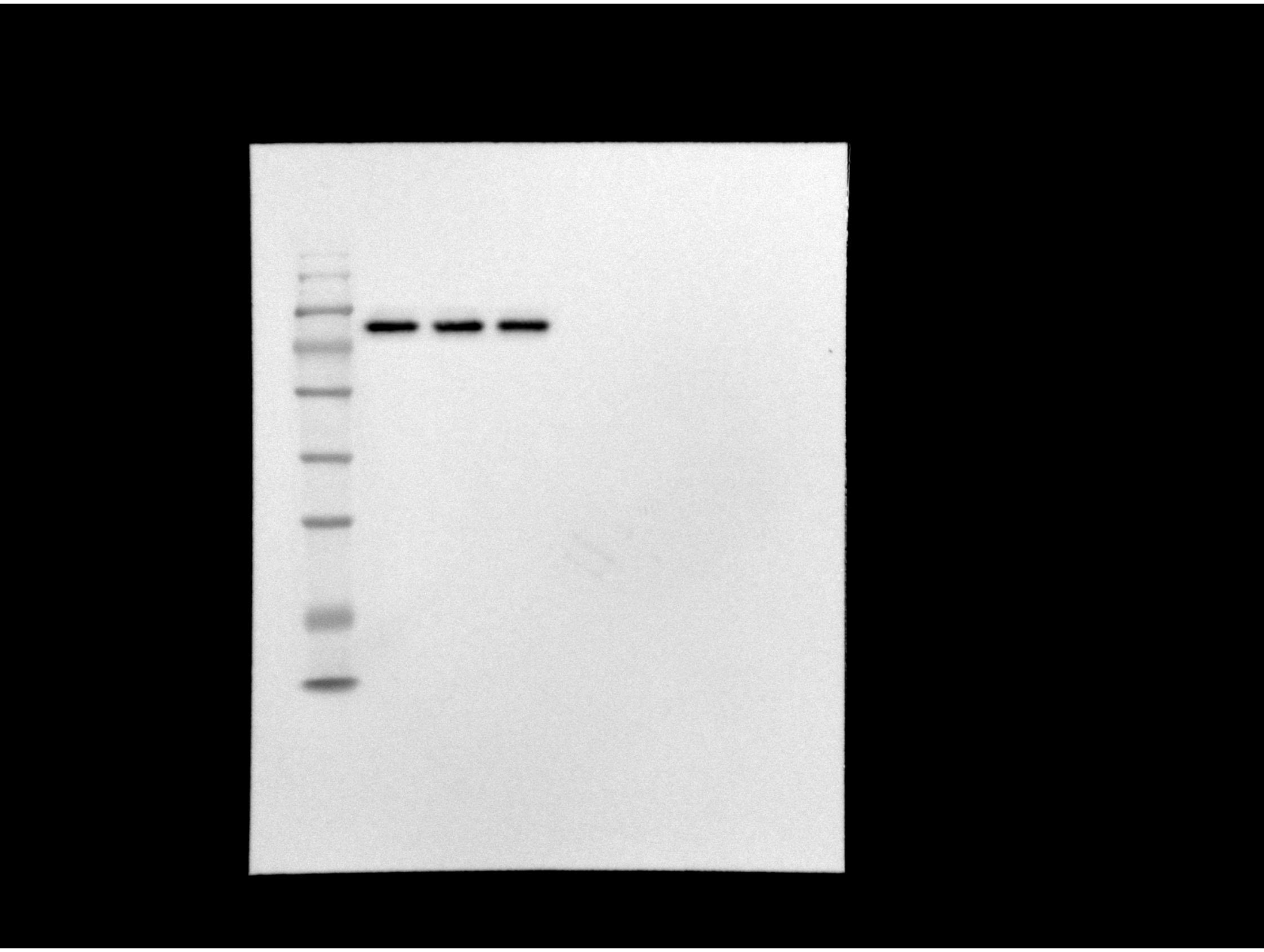

Supplementary Figure 3F

GAPDH

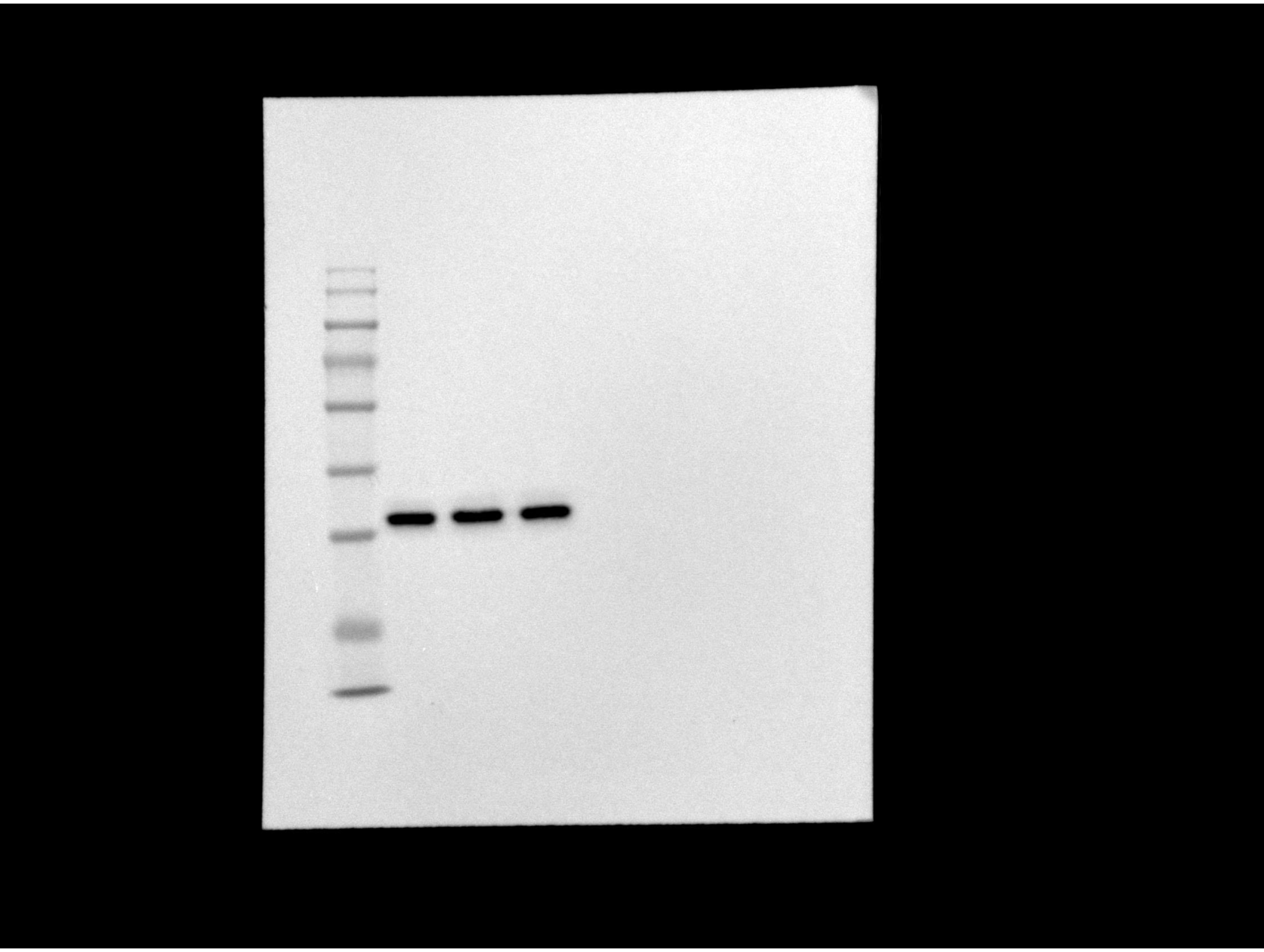

Supplementary Figure 3G

Prkar2a

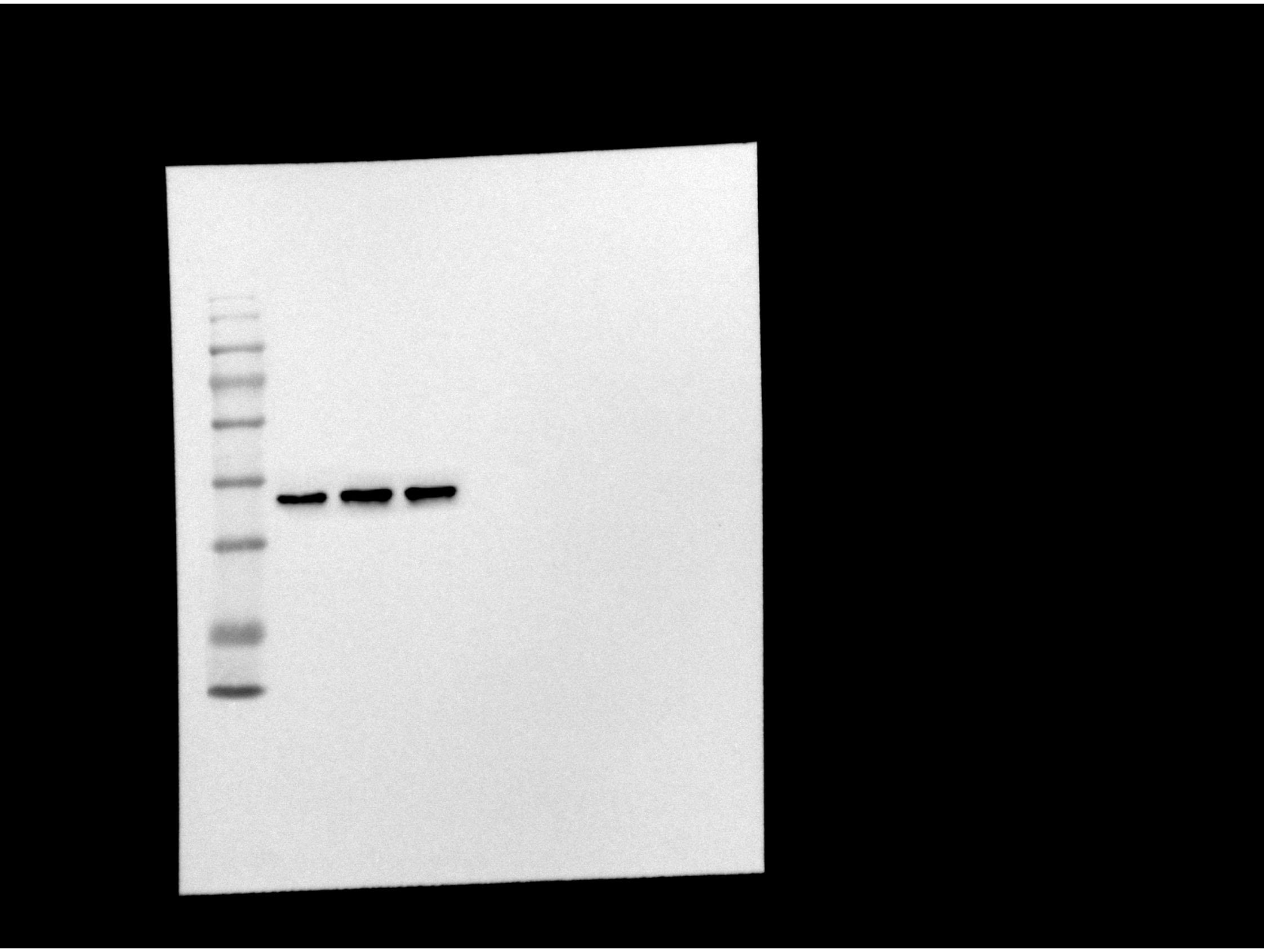

Supplementary Figure 3G

p-STAT1

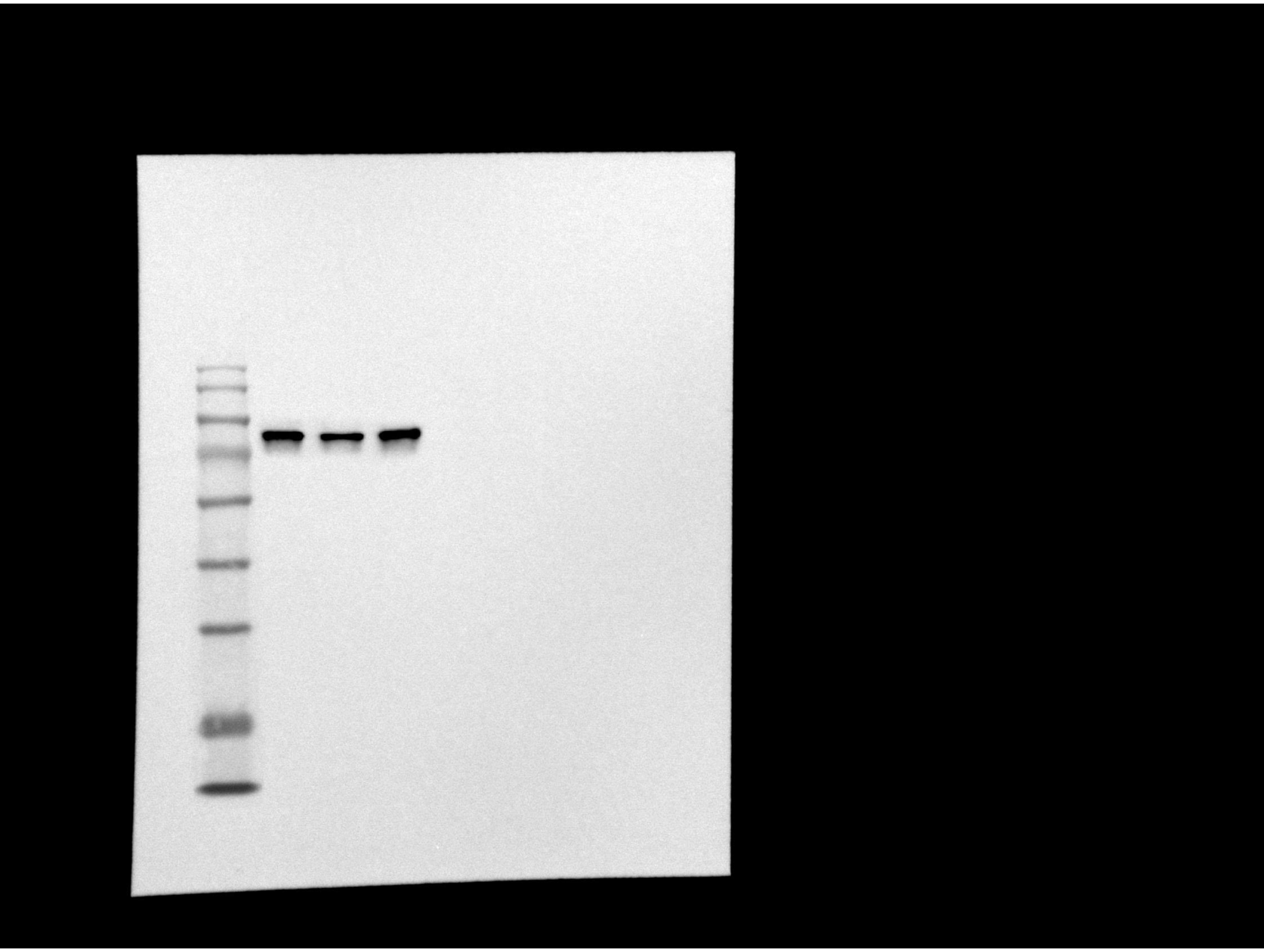

Supplementary Figure 3G

STAT1

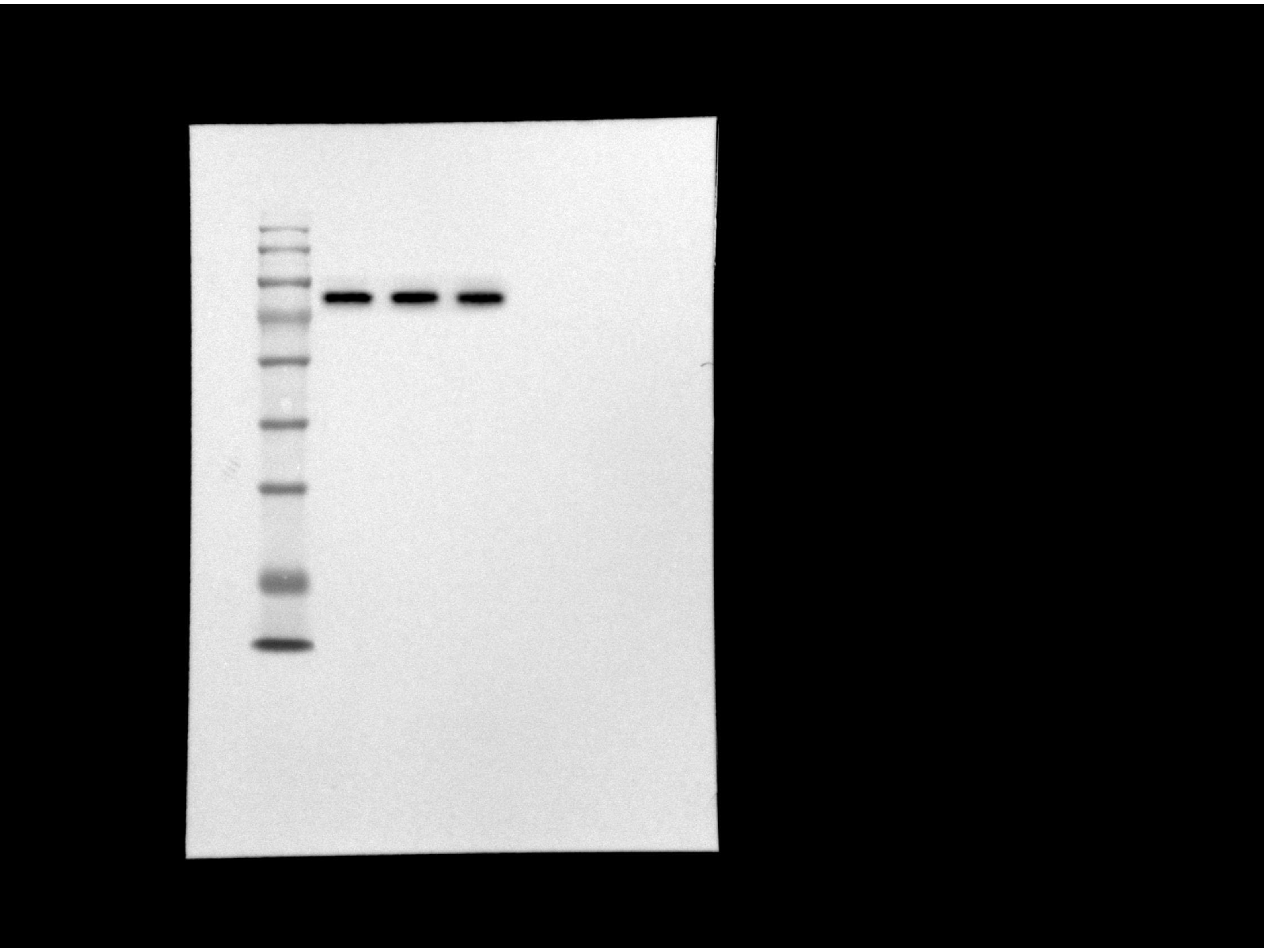

Supplementary Figure 3G

GAPDH

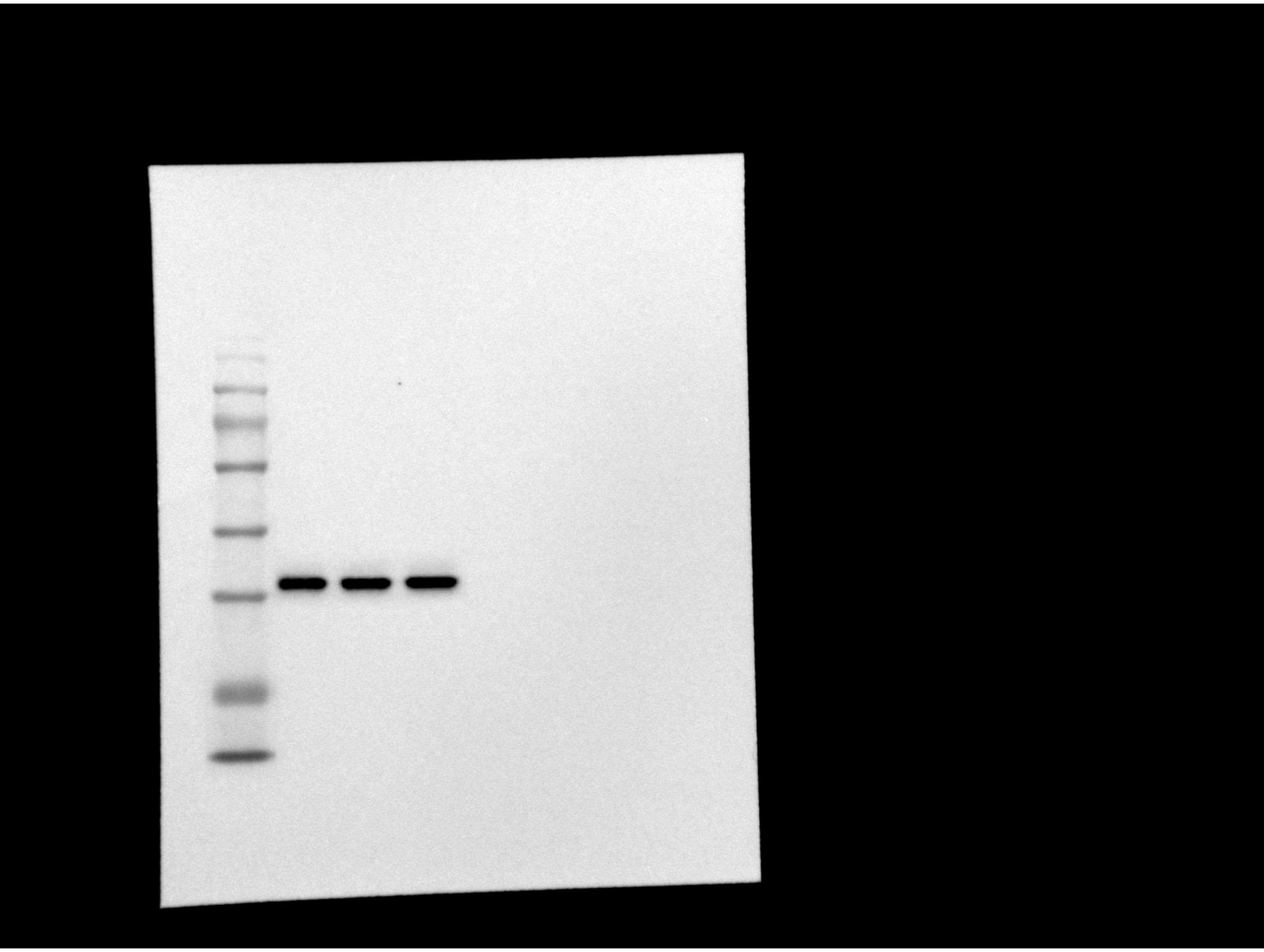

Supplement: Supplementary file 5 — Original gel blot images [file 41420_2022_1016_MOESM5_ESM.pdf]
